# Reduction of a dihydroboryl cation to a boryl anion and its air-stable, neutral hydroboryl radical through hydrogen shuttling†
†Electronic supplementary information (ESI) available: Synthetic procedures, NMR, EPR, UV-vis, IR, CV, X-ray crystallographic data and details of the computational analyses. CCDC 1956847–1956854. For ESI and crystallographic data in CIF or other electronic format see DOI: 10.1039/c9sc05026d


**DOI:** 10.1039/c9sc05026d

**Published:** 2019-11-25

**Authors:** Stephan Hagspiel, Merle Arrowsmith, Felipe Fantuzzi, Alexander Hermann, Valerie Paprocki, Regina Drescher, Ivo Krummenacher, Holger Braunschweig

**Affiliations:** a Institut für Anorganische Chemie , Julius-Maximilians-Universität Würzburg , Am Hubland , 97074 Würzburg , Germany . Email: h.braunschweig@uni-wuerzburg.de; b Institute for Sustainable Chemistry & Catalysis with Boron , Julius-Maximilians-Universität Würzburg , Am Hubland , 97074 Würzburg , Germany; c Institut für Physikalische und Theoretische Chemie , Julius-Maximilians-Universität Würzburg , Emil-Fischer-Straße 42 , 97074 Würzburg , Germany

## Abstract

The addition of Lewis bases to a cyclic (alkyl)(amino)carbene (CAAC)-supported dihydroboron triflate yields the mixed doubly base-stabilised dihydroboryl cations [(CAAC)BH_2_L]^+^. Of these, [(CAAC)_2_BH_2_]OTf (OTf = triflate) underwent facile two-electron reduction with KC_8_ owing to a 1,2-hydride migration from boron to the carbene carbon to yield a stable hydroboryl anion. One-electron oxidation of the latter yielded the first neutral hydroboryl radical, which is bench-stable in the solid state.

## Abstract 2

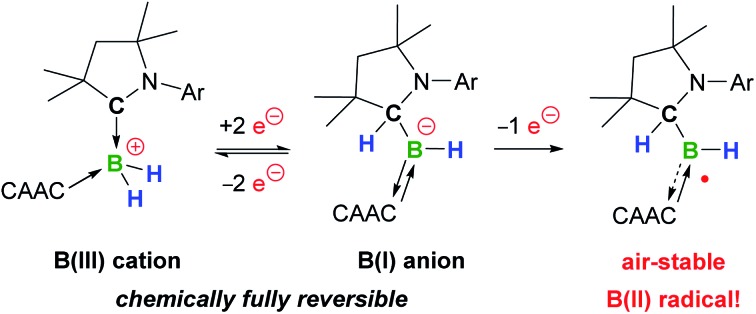
A doubly cyclic (alkyl)(amino)carbene-stabilised dihydroboronium cation undergoes fully reversible 2e^–^ reduction to a stable hydroboryl anion *via* B-to-C hydrogen migration. Subsequent 1e^–^ oxidation yields a bench-stable neutral hydroboryl radical.

## Introduction

**Fig. 1:**
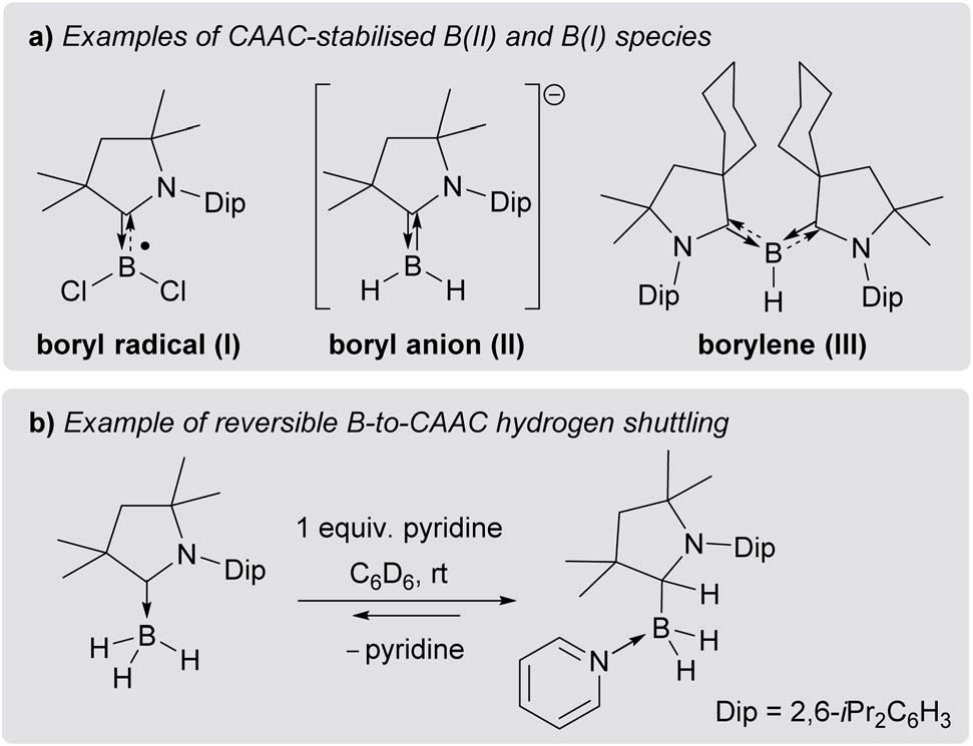
(a) Selected examples of CAAC-stabilised B(ii) and B(i) species; (b) example of reversible Lewis-base-induced B-to-CAAC hydrogen shuttling.

Cyclic (alkyl)(amino)carbenes (CAACs) have become the ligands of choice for the stabilisation of many main group compounds in low oxidation states owing to their excellent σ-donor and π-acceptor properties derived from a relatively high-lying HOMO and low-lying LUMO.^1^–^4^ In the field of low-valent mononuclear boron chemistry, they have been successfully employed to synthesise unusual boron(ii) species such as boryl radicals ([(CAAC)BXY]˙; X, Y = anionic ligands, *e.g.***I**, Fig. 1a),^5^–^10^ boryl radical cations ([(CAAC)LBY]˙^+^, L = Lewis donor)^10^–^13^ and boryl anions ([(CAAC)BXY]^–^, *e.g.***II**),^14^–^17^ as well as boron(i) species such as borylenes ((CAAC)LBX, *e.g.***III**, and (CAAC)BNR_2_).^6^–^8^,^11^–^13^,^16^,^18^–^20^ In all these compounds, the accumulation of negative charge on the low-valent boron centre is stabilised through π backbonding to the CAAC ligand(s) (Fig. 1a), making many of them surprisingly stable under inert conditions.^1^–^4^ Recently, transient dicoordinate (CAAC)-stabilised borylenes have drawn particular attention as compounds capable of activating and catenating N_2_,^21^–^25^ the latter reaction being unprecedented even in transition metal chemistry.

Furthermore, CAACs have been shown to activate element-hydrogen σ bonds, including H–H, N–H, P–H, Si–H and B–H by addition to their nucleophilic carbene carbon.^3^,^4^ In CAAC-supported hydroboron compounds, the B–H bond activation process can be reversible (Fig. 1b)^14^,^26^,^27^ and is favoured by electron-donating ligands at boron,^8^,^26^–^30^ thereby affording additional stabilisation for electron-rich lower oxidation state species through facile hydrogen shuttling. In this contribution we combine the excellent σ-donating/π-accepting and B–H bond activating properties of CAACs to synthesise and isolate a solvent-free alkyl(hydro)boryl anion, and selectively oxidise it to the corresponding radical, which is surprisingly air-stable in the solid state.

## Results and discussion‡The boron-bound hydrides of each structure were detected as residual electron density in the difference Fourier map and freely refined.


**Scheme 1:**
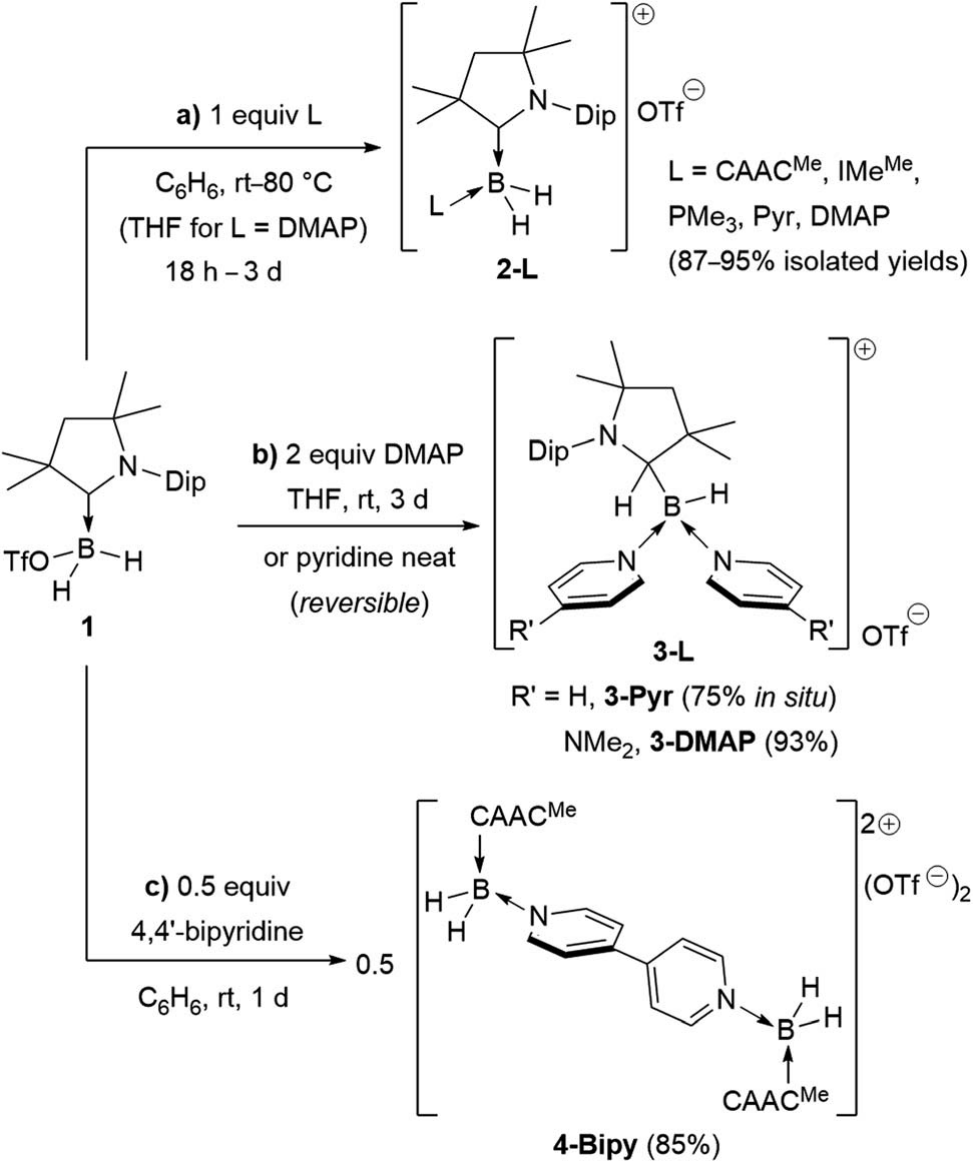
Syntheses of bis- and tris(base)-stabilised boronium cations (a) **2-L**, (b) **3-L** and (c) **4-L**. Isolated yields in brackets. IMe^Me^ = 1,3,4,5-tetramethylimidazol-2-ylidene, Pyr = pyridine, DMAP = 4-dimethylaminopyridine.

**Fig. 2:**
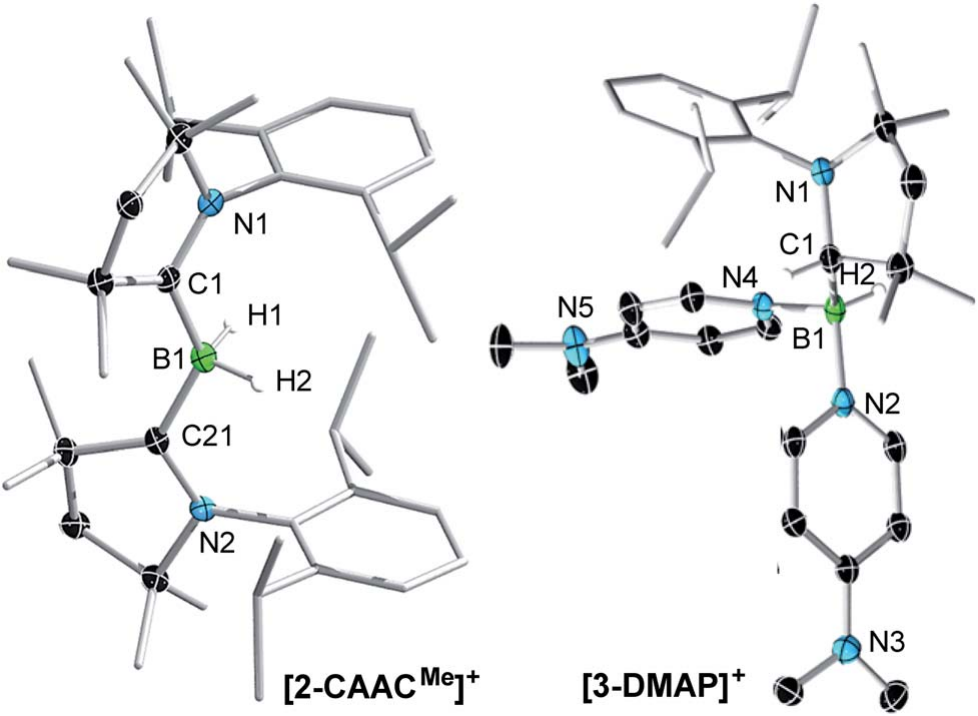
Crystallographically derived molecular structures of the **2-CAAC^Me^** (one of the two crystallographically distinct cations present in the asymmetric unit) and **3-DMAP** cations. Atomic displacement ellipsoids are set at 50% probability. Ellipsoids of CH_3_ and iPr groups, triflate counteranion and hydrogen atoms omitted for clarity except for boron-bound hydrides.‡ Selected bond lengths (Å) for **2-CAAC^Me^**: B1–C1 1.597(7), B1–C21 1.607(7), B1–H1 1.11(6), B1–H2 1.16(6), C1–N1 1.316(6), C21–N2 1.310(6); for **3-DMAP** B1–C1 1.619(4), B1–N2 1.585(3), B1–N4 1.597(3), B1–H2 1.10(2), C1–N1 1.490(3).

Following a procedure by Bertrand and co-workers,^12^ methyl trifluoromethanesulfonate (MeOTf) was employed to abstract a hydride from (CAAC^Me^)BH_3_ (CAAC^Me^ = 1-(2,6-diisopropylphenyl)-3,3,5,5-tetramethylpyrrolidin-2-ylidene). The resulting triflate derivative **1** was treated in a 1 : 1 ratio with a series of Lewis bases in benzene to generate the bis(base)-stabilised boronium cations [(CAAC^Me^)BH_2_L]OTf (**2-L**,L = CAAC^Me^, IMe^Me^ = 1,3-dimethylimidazol-2-ylidene, PMe_3_, Scheme 1a), all presenting a characteristic upfield ^11^B NMR *B*H_2_ triplet in the –22 to –30 ppm region.§
§The X-ray crystallographically-determined structures of **1**, **2-Pyr** and **2-DMAP** can be found in the ESI, Fig. S55–S57.†
 In the case of the 4-dimethylaminopyridine (DMAP) derivative, **2-DMAP** (*δ*_^11^B_ = –10.6 ppm, broad), the synthesis had to be carried out in THF as treatment of **1** with one equivalent of DMAP in benzene resulted in the formation of the bis(DMAP) adduct **3-DMAP** (*δ*_^11^B_ = 4.2 ppm, Scheme 1b), in which the second DMAP equivalent has promoted a typical 1,2-migration of one hydrogen atom from boron to the CAAC^Me^ ligand.^26^ The solid-state structure of **3-DMAP** (Fig. 2) evidences the binding of the DMAP residues and the migration of H1 to C1, which is now sp^3^-hybridised (B1–C1 1.619(4), C1–N1 1.490(3) Å). In contrast, the binding of a second equivalent of pyridine to **2-Pyr** (*δ*_^11^B_ = –9.3 ppm, broad) was found to be reversible: even in neat pyridine only *ca.* 75% conversion to **3-Pyr** (*δ*_^11^B_ = 6.9 ppm) was observed. The use of 4,4′-bipyridine as a base led to the formation of the 4,4′-bipyridine-bridged bis(boronium) species **4-Bipy** (*δ*_^11^B_ = –8.6 ppm, broad, Scheme 1c). Attempts to synthesise the derivative **2-thf** in THF resulted in ring-opening polymerisation of the solvent within two days at room temperature.

**Scheme 2:**
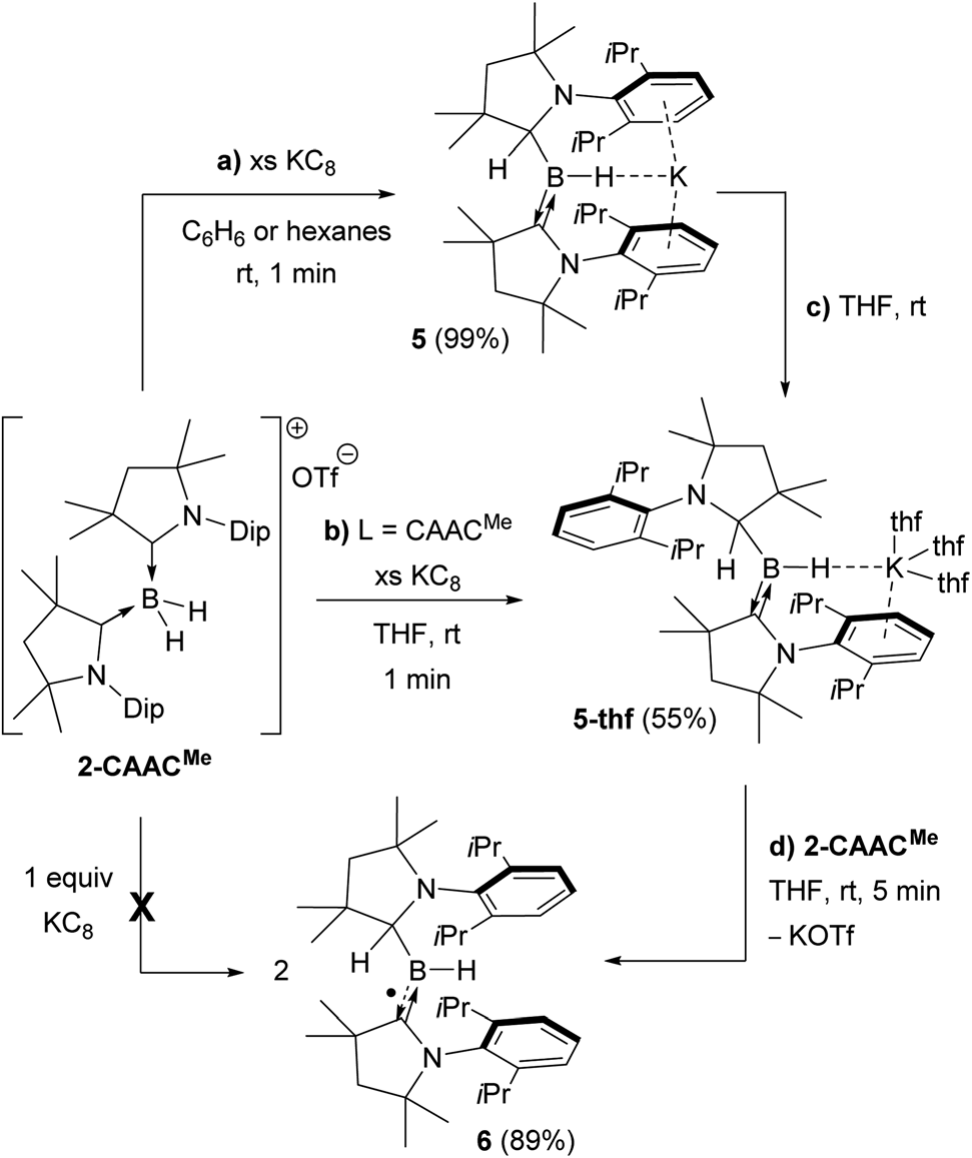
Reduction of **2-CAAC^Me^** to boryl anions (a) **5** and (b)–(c) **5-thf**, and subsequent comproportionation to (d) boryl radical **6**.

**Fig. 3:**
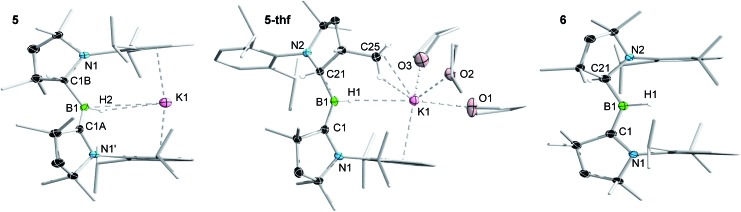
Crystallographically derived molecular structures of **5**, **5-thf** and **6**. Atomic displacement ellipsoids are set at 50% probability. Ellipsoids of CH_2_, CH_3_ and iPr groups and hydrogen atoms omitted for clarity except for boron-bound hydrides.‡ Selected bond lengths (Å) and angles (°) for **5**: B1–C1A 1.439(11), B1–C1B 1.633(9), B1–H2 1.14(3), C1A-N1′ 1.450(7), C1B–N1 1.520(8), K1···H1 2.53(3), K1···B1 3.141(4), K1···centroid 2.91, Σ∠B1 359.4(12), Σ∠C1A 359.7(5), B1–H2–K1 111.8(12); for **5-thf**: B1–C1 1.452(2), B1–C21 1.620(2), B1–H1 1.159(17), C1–N1 1.4601(18), C21–N2 1.5076(19), K1···H1 2.653(16), K1···B1 3.599(2), K1···centroid 2.95, K1···C25 3.2933(17), Σ∠B1 359.9(1), Σ∠C1 359.9(1), B1–H1–K1 138(1); for **6**: B1–C1 1.5174(18), B1–C21 1.5817(18), B1–H1 1.142(18), C1–N1 1.3777(15), C21–N2 1.4616(15), Σ∠B1 359.5(6), Σ∠C1 359.6(1).

**Fig. 4:**
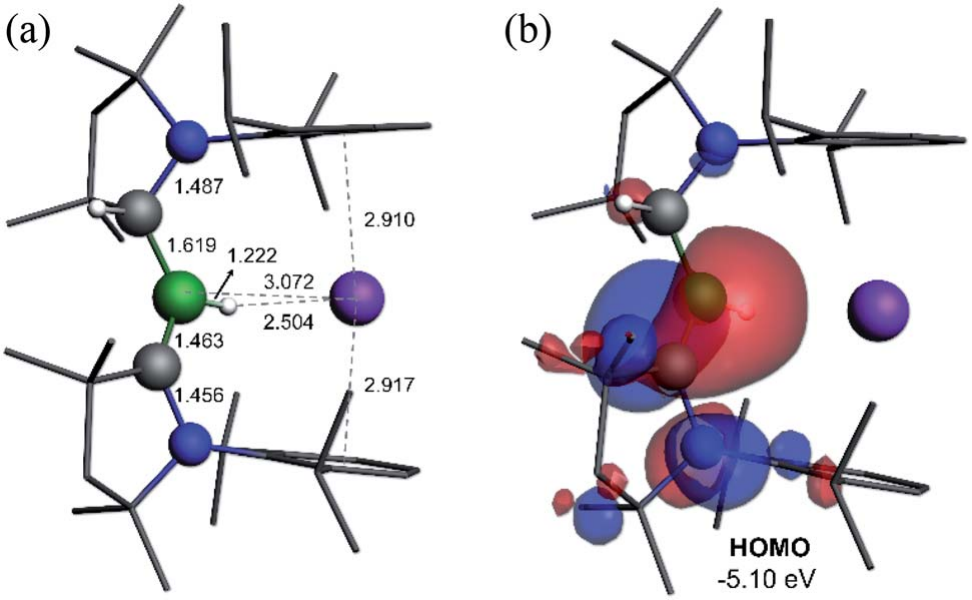
(a) Calculated structure of **5** at the ωB97XD/6-31+G* level of theory. (b) Plot of the HOMO of **5** (ωB97XD/6-311++G**).

Attempts to reduce **2-L**, **3-L** and **4-L** under various conditions all resulted in unselective reactions, except for **2-CAAC^Me^**, which was readily reduced with excess KC_8_ to the red-coloured (alkyl)hydroboryl anion **5** by 1,2-migration of one hydrogen atom from boron to CAAC^Me^ (Scheme 2a). The ^11^B NMR spectrum of **5** shows a single broad resonance at 16.7 ppm, significantly downfield-shifted from that of other CAAC-stabilised boryl anions, which range from *δ*_^11^B_ = –4.7 ppm for [(CAAC^Me^)BH_2_]^–^ to *δ*_^11^B_ = –17.9 ppm for [(CAAC^Cy^)B(CN)_2_]^–^,^14^–^17^ likely because of the electron-withdrawing nature of the aminoalkyl substituent CAAC^Me^H. The ^1^H{^11^B} NMR spectrum shows a B*H* doublet at 1.90 ppm (^3^*J* = 6.6 Hz), coupling to the BC*H* resonance of the CAAC^Me^H ligand at 4.38 ppm, as well as two sets of unsymmetrical CAAC^Me^ ligand resonances. An X-ray crystallographic analysis revealed a monomeric structure with a trigonal-planar boron atom (Σ∠B1 359(1)°), in which the potassium cation bound to the B*H* hydride (K1···H2 2.53(3) Å) is encapsulated by the ligand sphere through η^6^–π interactions with the Dip (=2,6-diisopropylphenyl) substituents of the CAAC^Me^ and CAAC^Me^H ligands (Fig. 3). The B1–C1A bond length of 1.439(11) Å is significantly shorter than in the **2-CAAC^Me^** precursor (B–C_avg._ 1.69 Å, Fig. 2) and typical of a B=C double bond. This is indicative of strong π backdonation from the lone pair of the boryl anion to the π-accepting CAAC^Me^ ligand, as found in all CAAC-stabilised boryl anions.^6^,^14^–^17^ According to DFT calculations carried out at the ωB97XD/6-31+G* level of theory, the HOMO of **5** possesses π-bonding character between B1 and C1A, with a nodal plane located at the C1A-N1′ bond region (Fig. 4). As in **3-DMAP**, a 1,2-hydride shift has occurred and C1B is now sp^3^-hybridised (B1–C1B 1.633(9), N1–C1B 1.520(8) Å). The presence of the hydrogen atom at boron was further confirmed by a solid-state infrared absorption at 2329 cm^–1^, corresponding to the B–H stretching mode. The computed B–H stretching mode of 2352 cm^–1^ at ωB97XD/6-31+G* agrees well with the experimental value.

The reduction of **2-CAAC^Me^** in THF or the dissolution of **5** in THF both yielded the analogue **5-thf** (Scheme 2b, c and Fig. 3), in which the hydride-bound potassium cation is η^6^–π-stabilised now only by the Dip substituent of the neutral CAAC^Me^ ligand, its coordination sphere being completed by three THF molecules and an agostic interaction with one of the vicinal methyl groups (C25) of the CAAC^Me^H ligand. The bond lengths and angles of the boryl anion core change little compared to those of solvent-free **5**, the major difference being the conformation of the pyrrolidine rings of CAAC^Me^H and CAAC^Me^, which flip so that the Dip substituents now point in opposite directions.

Cyclic voltammograms of **2-CAAC^Me^** and **5-thf** in THF (0.1 M [*n*Bu_4_N][PF_6_]) were essentially identical, showing a reversible redox event at *E*_1/2_ = –2.31 V and an irreversible oxidation around –0.90 V (relative to Fc/Fc^+^), suggesting that chemical oxidation of **5** to **6** should be possible. Indeed, the reaction of **5-thf** with **2-CAAC^Me^** led to quantitative comproportionation to the boryl radical **6** (Scheme 2d). Attempts to generate **6** by the direct one-electron reduction of **2-CAAC^Me^** failed, resulting instead in incomplete consumption of **2-CAAC^Me^** and generating a mixture of **5** and **6**. Radical **6** is deep purple in solution (*λ*_max_ = 523 nm in the UV-vis spectrum) and ^11^B NMR-silent. In the solid state, however, isolated crystals of **6** are deep orange. X-ray diffraction analysis showed a structure very similar to **5** bar the potassium cation, with a trigonal planar B1 centre (Σ∠B1 359.5(6)°) and the Dip groups of the CAAC^Me^H and CAAC^Me^ ligands both pointing in the same direction (Fig. 3). Unlike in **5** and **5-thf**, the B1–C1 and C1–N1 bonds at the neutral CAAC^Me^ ligand (1.5174(18) and 1.4601(18) Å, respectively) are within the range typical of partial double bonds, as is typical for CAAC-stabilised boryl radicals due to the delocalisation of the unpaired electron over the N1–C1–B1 π framework.^5^–^9^,^21^,^22^,^31^–^33^

**Fig. 5:**
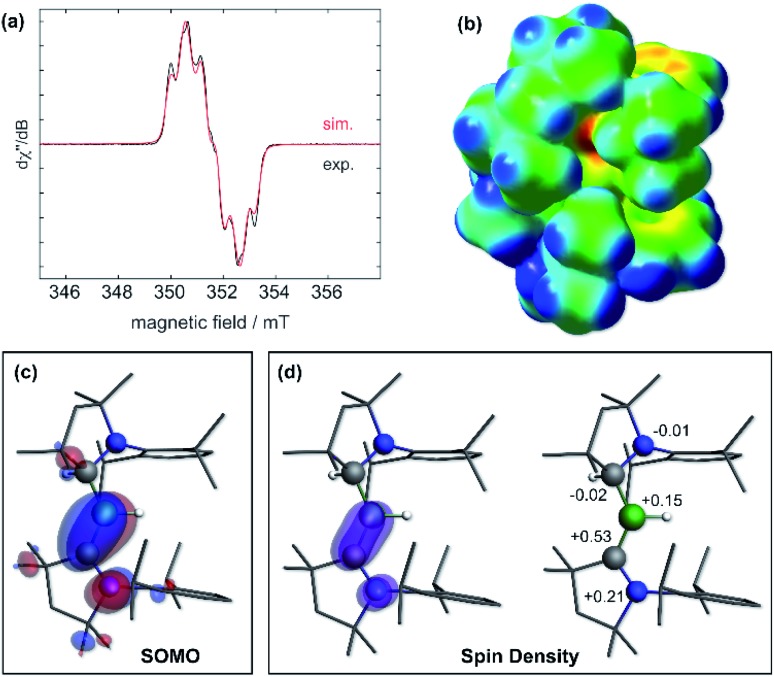
(a) Experimental (black solid line) and simulated (red line) continuous-wave X-band EPR spectra of **6** in hexane solution at rt. *Simulation parameters*: *g*_iso_ = 2.0027, *a*(^11^B) = 9.7 MHz, *a*(^14^N) = 18.5 MHz, *a*(^1^H_(H1)_) = 13.6 MHz and *a*(^1^H_(H21)_) = 4.8 MHz; (b) electrostatic potential (ESP) map of **6** at the ωB97XD/6-31+G* level of theory. ESP charges following the notation of Fig. 3: N2: –0.46, C21: –0.01, B1: +0.19, H1: –0.17, C1: –0.27, N1: –0.14. (c) Plot of the SOMO of **6** (surface isovalue: ± 0.03 [e a_0_^–3^]^1/2^). (d) Left: plot of the calculated spin density of **6** (surface isovalue: 0.005 [e a_0_^–3^]). Right: Mulliken atomic spin densities.

The IR spectrum of **6** shows a B–H stretching band at 2533 cm^–1^ (calc.: 2558 cm^–1^ at ωB97XD/6-31+G*), *ca.* 200 wavenumbers higher than that in **5**, and 100 higher than in Bertrand's hydroborylene **III** (Fig. 1a, *ν*(B–H) = 2455 cm^–1^), suggesting a significant strengthening of the B–H bond in radical **6**. The EPR spectrum of **6** displays a broad triplet from the hyperfine coupling to the ^14^N nucleus (*a*_^14^N_ = 18.5 MHz, Fig. 5a). The simulated spectrum further provides hyperfine coupling parameters to the quadrupolar ^11^B nucleus (*a*_^11^B_ = 9.7 MHz), which is responsible for the line-broadening, and the B*H* and CAAC^Me^*H*^1^H nuclei (*a*_^1^H_ = 13.6 and 4.8 MHz, respectively). The presence of two distinct couplings to these ^1^H nuclei suggests that the compound displays no fluxional B-to-CAAC hydrogen migration in solution.

Calculations show that the SOMO consists mainly of the B1–C1 π bond with some π-antibonding character on the C1–N1 bond (Fig. 5c). The calculated Mulliken atomic spin densities are 53% on C1, 21% on N1 and only 15% on B1, showing that the unpaired electron is mainly delocalised on the CAAC ligand (Fig. 5d), as already suggested by the much stronger EPR hyperfine coupling to N1 than B1 (*vide supra*). To our knowledge, **6** is the first example of a neutral, structurally characterised hydroboryl radical. Moreover, to our surprise, isolated crystals of **6** proved air-stable at room temperature over a period of one week, making this compound a rare example of an air-stable boron-centred radical. This is presumably owed to a combination of the high degree of spin delocalisation, the low spin density at boron and the very effective encapsulation of the B–H unit by the CAAC^Me^ and CAAC^Me^H ligands as seen in the electrostatic potential map in Fig. 5b. The only other air-stable boron-based radical reported is a permethylated icosahedral borane [*closo*-B_12_(CH_3_)_12_]˙^–^ radical anion, in which the unpaired electron is trapped and delocalised within the B_12_ cage.^34^

**Scheme 3:**
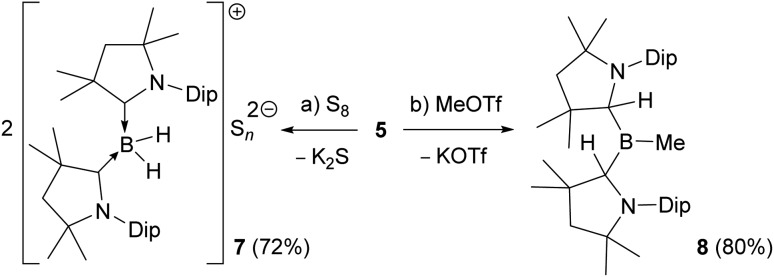
(a) Reducing and (b) nucleophilic reactivity of boryl anion **5**.

Reactions of the boryl anion **5** with a wide range of electrophiles including haloboranes, organohalides, heavier group 14 chlorides, as well as Zn(ii), Cu(i) and Au(i) halides all resulted in quantitative oxidation of **5** to radical **6**, and reduction of the corresponding electrophile. This contrasts with the boron nucleophile behaviour observed for CAAC-stabilised cyanoboryl anions.^16^,^17^ With elemental sulfur, double oxidation back to the **2-CAAC^Me^** cation was observed by NMR spectroscopic analysis (*δ*_^11^B_ = –22.4 ppm, *t*, ^1^*J*_^11^B_–_^1^H_ = 84.7 Hz), the counteranion presumably being a S_*n*_^2–^ polysulfide (**7**, Scheme 3a). The only nucleophilic reactivity observed was with methyl triflate, which yielded clean salt metathesis to the methylated trialkylborane **8** through migration of the second hydride to the remaining CAAC^Me^ ligand (*δ*_^11^B_ = 93.9 ppm, Scheme 3b).

## Conclusions

We have shown herein that the ability of CAACs to stabilise electron-rich boron centres and reversibly activate B–H bonds can be harnessed together to reduce a [L_2_BH_2_]^+^ cation to a [LRBH]^–^ anion without the usual need for halide abstraction, thanks to B-to-CAAC hydrogen shuttling. This boryl anion reacts principally as a one-electron reducing agent to yield the neutral hydroboryl radical [LRBH]˙, the surprising stability of which is ensured by the unique stereoelectronic properties of the two encapsulating CAAC^Me^ ligands.

## Conflicts of interest

The authors declare no conflict of interest.

## Supplementary Material

Supplementary information

Crystal structure data
