# Supplementary material for: Reduction of a dihydroboryl cation to a boryl anion and its air-stable, neutral hydroboryl radical through hydrogen shuttling
Source: Chem Sci. 2019 Nov 25;11(2):551–5. doi: 10.1039/c9sc05026d (PMC7069503; doi:10.1039/c9sc05026d)
Supplement: Supplementary file 1 [file SC-011-C9SC05026D-s001.pdf]

Electronic Supplementary Material (ESI) for Chemical Science.
This journal is © The Royal Society of Chemistry 2019

Supporting Information for:

Reduction of a dihydroboryl cation to a boryl anion and its air-stable, neutral hydroboryl radical through hydrogen shuttling

Stephan Hagspiel,[a,b] Merle Arrowsmith,[a,b] Felipe Fantuzzi,[a,b,c] Alexander Hermann,[a,b] Valerie Paprocki,[a,b] Regina Drescher,[a,b] Ivo Krummenacher,[a,b] Holger Braunschweig*,[a,b]

[a] *Institut für Anorganische Chemie, Julius-Maximilians-Universität Würzburg, Am Hubland, 97074 Würzburg (Germany). E-mail: h.braunschweig@uni-wuerzburg.de*

[b] *Institute for Sustainable Chemistry & Catalysis with Boron, Julius-Maximilians-Universität Würzburg, Am Hubland, 97074 Würzburg (Germany).*

[c] *Institut für Physikalische und Theoretische Chemie, Julius-Maximilians-Universität Würzburg, Emil-Fischer-Straße 42, 97074 Würzburg (Germany).*

Table of contents

Methods and materials 2
Synthetic procedures 3
NMR spectra of isolated compounds 11
IR spectra 57
UV-vis spectra 59
Cyclic voltammetry 60
EPR measurement 61
X-ray crystallographic data 62
Computational details 67
References 83

Methods and materials

All manipulations were performed either under an atmosphere of dry argon or *in vacuo* using standard Schlenk line or glovebox techniques. Deuterated solvents were dried over molecular sieves and degassed by three freeze-pump-thaw cycles prior to use. All other solvents were distilled and degassed from appropriate drying agents. Solvents were stored under argon over activated 4 Å molecular sieves. NMR spectra were acquired on a Bruker Avance 500 NMR spectrometer. Chemical shifts (δ) are yield in ppm and internally referenced to the carbon nuclei ($^{13}C\{^{1}H\}$) or residual protons (^{1}H) of the solvent. Hetero nuclei NMR spectra are referenced to external standards (^{11}B: $BF_3{\cdot}OEt_2$; ^{19}F: Cl_3CF; ^{31}P: 85% H_3PO_4). Solid-state IR spectra were recorded on a Jasco FT/IR-6200 spectrometer inside a glovebox. Microanalyses (C, H, N, S) were performed on an Elementar vario MICRO cube elemental analyzer. High-resolution mass spectrometry (HRMS) data were obtained from a Thermo Scientific Exactive Plus spectrometer. *Note: both elemental analyses and HRMS were carried out for all new compounds but in some cases these decomposed too rapidly and only one type of analysis was possible.*

Solvents and reagents were purchased from Sigma-Aldrich or Alfa Aesar. $CAAC^{Me}$ (1-(2,6-di*iso*propylphenyl)-3,3,5,5-tetramethylpyrrolidin-2-ylidene),[1] $(CAAC^{Me})BH_3$[2] and IMe^{Me} (1,3,4,5-tetramethylimidazol-2-ylidene)[3] were synthesised using literature procedures.

Synthetic procedures

(CAAC^{Me})BH$_2$(OTf), 1

MeOTf (2.03 mL, 18.5 mmol, 1.5 eq.) was added slowly to a solution of (CAAC^{Me})BH$_3$ (3.70 g, 12.3 mmol) in 60 mL of benzene whereupon a gas evolution occurred. The reaction mixture was stirred overnight prior to removal of volatiles. The residue was washed with hexane and dried *in vacuo* to yield **1** as a white solid (5.30 g, 11.8 mmol, 96% yield). Colorless single crystals were obtained by vapor diffusion of hexane into a saturated benzene solution. ^{1}H{^{11}B} NMR (500 MHz, C$_6$D$_6$, 297 K): δ = 7.06 (t, ^3J = 7.8 Hz, 1H, *p*-Ar*H*), 6.92 (d, ^3J = 7.8 Hz, 2H, *m*-Ar*H*), 3.28 (broad s, 2H, B*H*$_2$), 2.42 (sept, ^3J = 6.7 Hz, 2H, *i*Pr-C*H*), 1.41 (s, 6H, NC(C*H*$_3$)$_2$), 1.33 (s, 2H, C*H*$_2$), 1.25 (d, ^3J = 6.7 Hz, 6H, *i*Pr-C*H*$_3$), 1.02 (d, ^3J = 6.7 Hz, 6H, *i*Pr-C*H*$_3$), 0.71 (s, 6H, C(C*H*$_3$)$_2$) ppm. ^{13}C{^{1}H} NMR (126 MHz, C$_6$D$_6$, 297 K): δ = 193.0 (C$_{carbene}$, identified by HMBC), 144.7 (*i*-Ar*C*), 131.7 (*o*-Ar*C*), 130.2 (*p*-Ar*C*), 125.3 (*m*-Ar*C*), 120.2 (q, $^1J_{13C\text{-}19F}$ = 319 Hz, *C*F$_3$), 78.3 (N*C*(CH$_3$)$_2$), 52.5 (*C*(CH$_3$)$_2$), 51.1 (*C*H$_2$), 29.4 (*i*Pr-*C*H), 28.3 NC(*C*H$_3$)$_2$), 28.2 (C(*C*H$_3$)$_2$), 25.8 (*i*Pr-*C*H$_3$), 23.7 (*i*Pr-*C*H$_3$) ppm. ^{11}B NMR (160 MHz, C$_6$D$_6$, 297 K): δ = –6.32 (broad) ppm. ^{19}F NMR (471 MHz, C$_6$D$_6$, 297 K): δ = –76.2 (s) ppm. Elemental analysis for [C$_{21}$H$_{33}$BF$_3$NO$_3$S] (M$_w$ = 447.36): calcd. C 56.38, H 7.44, N 3.13, S 7.17%; found C 56.71, H 7.59, N 3.36, S 6.82%.

[(CAAC^{Me})BH$_2$(CAAC^{Me})]OTf, 2-CAAC^{Me}

a) Compound **1** (1.30 g, 2.91 mmol) and CAAC^{Me} (1.00 g, 3.49 mmol, 1.2 eq.) were dissolved in 30 mL of benzene and subsequently heated to 75 °C for 2 d. The resulting suspension was filtered and the solid obtained was washed with hexane. Drying *in vacuo* yielded **2-CAAC^{Me}** as a white solid (2.00 g, 2.73 mmol, 94% yield). Colorless single crystals were obtained by vapor diffusion of hexane in a saturated chloroform solution.

b) A solution of (CAAC^{Me})BH$_2$(CAAC^{Me}H) (20.0 mg, 34.2 μmol) in 1 mL of benzene was treated with an excess of MeOTf (5–10 eq.). Within 2 d at room temperature a suspension was formed and complete consumption of (CAAC^{Me})BH$_2$(CAAC^{Me}H) was observed by ^{11}B NMR spectroscopy. After filtration the solid obtained was washed with benzene and dried *in vacuo* affording **2-CAAC^{Me}** (22.8 mg, 31.1 μmol, 91% yield). The NMR spectra of the isolated solid were identical with those from synthesis a). ^{1}H NMR (500 MHz, CDCl$_3$, 238 K): δ = 7.27–7.17 (m, 4H, *m*-Ar*H*), 7.03 (s, 1H, *p*-Ar*H*), 7.01 (s, 1H, *p*-Ar*H*), 2.50 (sept, ^3J = 6.1 Hz, 2H, *i*Pr-C*H*), 2.34 (d, ^2J = 13.2 Hz, 2H, C*H*$_2$), 2.02 (d, ^2J = 13.1 Hz, 2H, C*H*$_2$), 1.90 (m, 2H, *i*Pr-C*H*), 1.88 (s, 6H, NC(C*H*$_3$)$_2$), 1.53 (s, 6H, NC(C*H*$_3$)$_2$), 1.44 (s, 6H, C(C*H*$_3$)$_2$), 1.31 (d, ^3J = 6.5 Hz, 6H,

*i*Pr-C*H*$_3$), 1.12–1.07 (m, 18H, C(C*H*$_3$)$_2$ and two *i*Pr-C*H*$_3$), 0.07 (d, ^3J = 6.4 Hz, 6H, *i*Pr-C*H*$_3$) ppm. ^{13}C{^{1}H} NMR (126 MHz, CDCl$_3$, 297 K): δ = 193.2 (C$_{carbenes}$, identified by HMBC), 144.8 (*i*-Ar*C*), 142.6 (*i*-Ar*C*), 132.7 (*o*-Ar*C*), 129.7 (*p*-Ar*C*), 125.8 (*m*-Ar*C*), 121.2 (q, $^1J_{13C-19F}$ = 321 Hz, *C*F$_3$), 79.2 (N*C*(CH$_3$)$_2$), 53.6 (*C*H$_2$), 51.9 (*C*(CH$_3$)$_2$), 29.8 (*i*Pr-*C*H), 29.4 (NC(*C*H$_3$)$_2$), 29.1 (C(*C*H$_3$)$_2$), 28.8 (C(*C*H$_3$)$_2$), 28.6 (NC(*C*H$_3$)$_2$), 27.2 (*i*Pr-*C*H$_3$), 25.7 (*i*Pr-*C*H$_3$), 24.4 (*i*Pr-*C*H$_3$), 24.3 (*i*Pr-*C*H$_3$) ppm. ^{11}B NMR (160 MHz, CDCl$_3$, 297 K): δ = –22.4 (t, $^1J_{11B-1H}$ = 85.3 Hz) ppm. ^{19}F NMR (471 MHz, CDCl$_3$, 297 K): δ = –78.0 (s) ppm. Solid-state IR: ν(B–H) = 2403 cm^{-1}. Elemental analysis for [$C_{41}H_{64}BF_3N_2O_3S$] (M$_w$ = 732.84): calcd. C 67.20, H 8.80, N 3.82, S 4.37%; found C 68.22, H 8.48, N 3.41, S 4.15%. HRMS LIFDI for [$C_{40}H_{64}BN_2$]$^+$ = [M – SO_3CF_3]$^+$: calcd. 583.5157; found 583.5140.

[(CAAC^{Me})BH$_2$(IMe^{Me})]OTf, 2-IMe^{Me}

A solution of **1** (100 mg, 0.22 mmol) and IMe^{Me} (27.8 mg, 0.22 mmol) in 10 mL of benzene was stirred at 60 °C overnight. The resulting suspension was cooled to room temperature and hexane was added to further induce precipitation. After filtration the white solid was washed with hexane and dried *in vacuo* yielding **2-IMe^{Me}** (109 mg, 0.19 mmol, 87% yield). ^{1}H{^{11}B} NMR (500 MHz, CDCl$_3$, 297 K): δ = 7.46 (t, ^3J = 7.8 Hz, 1H, *p*-Ar*H*), 7.33 (d, ^3J = 7.8 Hz, 2H, *m*-Ar*H*), 3.60 (s, 6H, IMe^{Me}-NC*H*$_3$), 2.66 (sept, ^3J = 6.6 Hz, 2H, *i*Pr-C*H*), 2.22 (s, 6H, IMe^{Me}-CC*H*$_3$), 2.17 (s, 2H, C*H*$_2$), 1.81 (s, 2H, B*H*$_2$), 1.41 (s, 6H, NC(C*H*$_3$)$_2$), 1.38 (d, ^3J = 6.6 Hz, 12H, *i*Pr-C*H*$_3$), 1.26 (s, 6H, C(C*H*$_3$)$_2$) ppm. ^{13}C{^{1}H} NMR (126 MHz, CDCl$_3$, 297 K): δ = 230.5 (CAAC-C$_{carbene}$, identified by HMBC), 159.1 (NHC-C$_{carbene}$, identified by HMBC), 144.7 (*i*-Ar*C*), 132.0 (*o*-Ar*C*), 130.1 (*p*-Ar*C*), 126.2(*C*CH$_3$), 125.8 (*m*-Ar*C*), 121.1 (q, $^1J_{13C-19F}$ = 321 Hz, *C*F$_3$), 79.1 (N*C*(CH$_3$)$_2$), 52.9 (*C*(CH$_3$)$_2$), 50.9 (*C*H$_2$), 33.9 (N*C*H$_3$), 29.4 (NC(*C*H$_3$)$_2$), 29.2 (*i*Pr-*C*H), 28.2 (C(*C*H$_3$)$_2$), 26.8 (*i*Pr-*C*H$_3$), 24.7 (*i*Pr-*C*H$_3$), 9.1 (C*C*H$_3$) ppm. ^{11}B NMR (160 MHz, CDCl$_3$, 297 K): δ = –29.2 (t, $^1J_{11B-1H}$ = 87.8 Hz) ppm. ^{19}F NMR (471 MHz, CDCl$_3$, 297 K): δ = –78.1 (s) ppm. Elemental analysis for [$C_{28}H_{45}BF_3N_3O_3S$] (M$_w$ = 571.55): calcd. C 58.84, H 7.94, N 7.35, S 5.61%; found C 58.45, H 7.92, N 7.75, S 5.42%. HRMS LIFDI for [$C_{27}H_{45}BN_3$]$^+$ = [M – SO_3CF_3]$^+$: calcd. 422.3701; found 422.3678.

[(CAAC^{Me})BH$_2$(PMe$_3$)]OTf, 2-PMe$_3$

A solution of **1** (100 mg, 0.22 mmol) and PMe$_3$ (0.03 mL, 0.27 mmol, 1.2 eq.) in benzene (3 mL) was stirred overnight at room temperature. After removal of all volatiles the solid obtained was washed with hexane and dried *in vacuo* to afford **2-PMe$_3$** (110 mg, 0.21 mmol, 95% yield). ^{1}H{^{11}B} NMR (500 MHz, CD$_2$Cl$_2$, 297 K): δ = 7.49 (t, ^3J = 7.8 Hz, 1H, *p*-Ar*H*),

7.33 (d, ^3J = 7.8 Hz, 2H, *m*-Ar*H*), 2.50 (sept, ^3J = 6.6 Hz, 2H, *i*Pr-C*H*), 2.23 (s, 2H, CH_2), 1.56 (s, 6H, NCH_3), 1.55 (d, $^2J_{1H-31P}$ = 11.4 Hz, 9H, P(CH_3)$_3$), 1.46 (d, $^2J_{1H-31P}$ = 23.5 Hz, 2H, PBH_2), 1.34 (d, ^3J = 6.6 Hz, 6H, *i*Pr-CH_3), 1.17 (d, ^3J = 6.6 Hz, 6H, *i*Pr-CH_3) ppm. ^{13}C{^{1}H} NMR (126 MHz, CD_2Cl_2, 297 K): δ = 223.8 ($C_{carbene}$, identified by HMBC), 143.0 (*i*-Ar*C*), 130.7 (*o*-Ar*C*), 129.2 (*p*-Ar*C*), 124.1 (*m*-Ar*C*), 119.9 (q, $^1J_{13C-19F}$ = 321 Hz, *C*F_3), 79.6 (N*C*(CH_3)$_2$), 51.3 (*C*(CH_3)$_2$), 49.4 (*C*H_2), 28.1 (*i*Pr-*C*H), 27.8 (NC(*C*H_3)$_2$), 27.7 (C(*C*H_3)$_2$), 25.0 (*i*Pr-*C*H_3), 22.1 (*i*Pr-*C*H_3), 11.9 (d, $^1J_{13C-31P}$ = 44.1 Hz, P(*C*H_3)$_3$) ppm. ^{11}B NMR (160 MHz, CD_2Cl_2, 297 K): δ = –27.8 (dt, $^1J_{11B-31P}$ = 81.5 Hz, $^1J_{11B-1H}$ = 89.8 Hz, determined by selective ^{1}H and ^{31}P-decoupling experiments) ppm. ^{19}F NMR (471 MHz, CD_2Cl_2, 297 K): δ = –78.9 (s) ppm. ^{31}P{^{1}H} NMR (202 MHz, CD_2Cl_2, 297 K): δ = –10.6 (broad m) ppm. Elemental analysis for [$C_{24}H_{42}BF_3NO_3PS$] (M_w = 523.44): calcd. C 55.07, H 8.09, N 2.68, S 6.12%; found C 54.99, H 7.71, N 3.00, S 5.67%.

[(CAAC^{Me})BH_2(C_5H_5N)]OTf, 2-Pyr

To a solution of **1** (100 mg, 0.22 mmol) in benzene (3 mL) pyridine (17.7 mg, 0.22 mmol) diluted in benzene (1 mL) was added dropwise. After stirring for 3 d at room temperature a suspension was formed and subsequently volatiles were removed *in vacuo*. The residue was washed with hexane and dried *in vacuo* to obtain **2-Pyr** as a white solid (105 mg, 0.20 mmol, 91% yield). Colorless single crystals were obtained by vapor diffusion of hexane in a saturated chloroform solution. ^{1}H{^{11}B} NMR (500 MHz, $CDCl_3$, 297 K): δ = 8.59 (d, ^3J = 5.3 Hz, 2H, Py-C^2*H*), 8.27 (t, ^3J = 7.8 Hz, 1H, Py-C^4*H*), 7.97 (t, ^3J = 7.8 Hz, 2H, Py-C^3*H*), 7.46 (t, ^3J = 7.8 Hz, 1H, *p*-Ar*H*), 7.32 (d, ^3J = 7.8 Hz, 2H, *m*-Ar*H*), 2.93 (broad s, 2H, BH_2), 2.76 (sept, ^3J = 6.6 Hz, 2H, *i*Pr-C*H*), 2.20 (s, 2H, CH_2), 1.42 (s, 6H, NC(CH_3)$_2$), 1.39 (d, ^3J = 6.5 Hz, 6H, *i*Pr-CH_3), 1.30 (d, ^3J = 6.6 Hz, 6H, *i*Pr-CH_3), 1.23 (s, 6H, C(CH_3)$_2$) ppm. ^{13}C{^{1}H} NMR (126 MHz, $CDCl_3$, 297 K): δ = 224.5 ($C_{carbene}$, identified by HMBC), 149.0 (Py-*C*^{2}), 144.7 (*i*-Ar*C*), 143.1 (Py-*C*^{4}), 131.5 (*o*-Ar*C*), 130.4 (*p*-Ar*C*), 127.7 (Py-*C*^{3}), 125.5 (*m*-Ar*C*), 121.1 (q, $^1J_{13C-19F}$ = 321 Hz, *C*F_3), 79.8 (N*C*(CH_3)$_2$), 53.5 (*C*(CH_3)$_2$), 50.8 (*C*H_2), 29.2 (*i*Pr-*C*H), 29.1 (C(*C*H_3)$_2$), 28.9 (NC(*C*H_3)$_2$), 26.4 (*i*Pr-*C*H_3), 23.9 (*i*Pr-*C*H_3) ppm. ^{11}B NMR (160 MHz, $CDCl_3$, 297 K): δ = –9.3 (broad) ppm. ^{19}F NMR (471 MHz, $CDCl_3$, 297 K): δ = –78.1 (s) ppm. Elemental analysis for [$C_{26}H_{38}BF_3N_2O_3S$] (M_w = 526.47): calcd. C 59.32, H 7.28, N 5.32, S 6.09%; found C 59.47, H 7.26, N 5.73, S 5.72%. HRMS LIFDI for [$C_{25}H_{38}BN_2$]$^+$ = [M – SO_3CF_3]$^+$: calcd. 377.3123; found 377.3099.

[(CAAC^{Me})BH$_2$(DMAP)]OTf, 2-DMAP

To a solution of **1** (100 mg, 0.22 mmol) in THF (3 mL) DMAP (27.3 mg, 0.22 mmol) dissolved in THF (1 mL) was added dropwise. The mixture was stirred for 3 d at room temperature prior to removal of volatiles. The solid obtained was washed with hexane and dried *in vacuo* to yield **2-DMAP** (111 mg, 0.19 mmol, 89% yield). Colorless single crystals were obtained by vapor diffusion of hexane in a saturated THF solution. *Note: The reaction with 1.0 eq. DMAP in benzene instead of THF also results in the formation of* ***3-DMAP*** *(see below), while 50% of* ***1*** *remain unreacted. While* ***3-DMAP*** *crystallizes quantitatively* ***1*** *remains in solution, thus enabling separation.* ^{1}H NMR (500 MHz, $CDCl_3$, 297 K): δ = 7.83 (d, ^3J = 7.5 Hz, 2H, *o*-DMAP-*H*), 7.44 (t, ^3J = 7.8 Hz, 1H, *p*-Ar*H*), 7.30 (d, ^3J = 7.8 Hz, 2H, *m*-Ar*H*), 6.79 (d, ^3J = 7.5 Hz, 2H, *m*-DMAP-*H*), 3.17 (s, 6H, DMAP-CH_3), 2.66 (sept, ^3J = 6.6 Hz, 2H, *i*Pr-C*H*), 2.13 (s, 2H, CH_2), 1.37 (s, 6H, NC(CH_3)$_2$), 1.36 (d, ^3J = 6.6 Hz, 6H, *i*Pr-CH_3), 1.30 (d, ^3J = 6.6 Hz, 6H, *i*Pr-CH_3), 1.26 (s, 6H, C(CH_3)$_2$) ppm. ^{13}C{^{1}H} NMR (126 MHz, $CDCl_3$, 297 K): δ = 227.8 ($C_{carbene}$, identified by HMBC), 155.8 (*i*-Ar*C*), 147.3 (*o*-DMAP-*C*), 144.5 (*o*-Ar*C*), 131.6 (*p*-DMAP-*C*), 130.2 (*p*-Ar*C*), 125.4 (*m*-Ar*C*), 121.2 (q, $^1J_{13C\text{-}19F}$ = 321 Hz, *C*F_3), 107.8 (*m*-DMAP-*C*), 79.2 (N*C*(CH_3)$_2$), 53.1 (*C*(CH_3)$_2$), 51.4 (*C*H_2), 39.9 (DMAP-*C*H_3), 29.3 (*i*Pr-*C*H), 29.0 (C(*C*H_3)$_2$), 28.9 (C(*C*H_3)$_2$), 26.4 (*i*Pr-*C*H_3), 24.0 (*i*Pr-*C*H_3) ppm. ^{11}B NMR (160 MHz, $CDCl_3$, 297 K): δ = –10.6 (broad) ppm. ^{19}F NMR (471 MHz, $CDCl_3$, 297 K): δ = –78.0 (s) ppm. HRMS LIFDI for $[C_{27}H_{43}BN_3]^+$ = $[M – SO_3CF_3]^+$: calcd. 420.3545; found 420.3525.

[(CAAC^{Me}H)BH(DMAP)$_2$]OTf, 3-DMAP

A solution of **1** (100 mg, 0.22 mmol) and DMAP (54.6 mg, 0.44 mmol, 2.0 eq.) in THF (4 mL) was stirred for 3 d at room temperature. The formed suspension was evaporated to dryness and the solid obtained was washed with hexane yielding **3-DMAP** as a white powder (142 mg, 0.20 mmol, 93% yield). Colorless single crystals were obtained by vapor diffusion of hexane in a saturated THF solution. ^{1}H NMR (400 MHz, $CDCl_3$, 297 K): δ = 7.73 (d, ^3J = 6.5 Hz, 2H, *m*-DMAP-*H*), 7.72 (broad, 2H, *o*-DMAP-*H*), 7.01 (d, ^3J = 7.3 Hz, 1H, *m*-Ar*H*), 7.03 (app. t, ^3J = 7.5 Hz, 1H, *p*-Ar*H*), 6.79 (d, ^3J = 7.6 Hz, 1H, *m*-Ar*H*), 6.52 (d, ^3J = 6.8 Hz, 2H, *m*-DMAP-*H*), 6.21 (d, ^3J = 6.5 Hz, 2H, *m*-DMAP-*H*), 3.75 (sept, ^3J = 6.4 Hz, 1H, *i*Pr-C*H*), 3.51 (d, ^3J = 5.7 Hz, 1H, BC*H*), 3.36 (sept, ^3J = 6.7 Hz, 1H, *i*Pr-C*H*), 3.08 (s, 6H, DMAP-CH_3), 3.00 (s, 6H, DMAP-CH_3), 2.24 (d, ^2J = 12.5 Hz, 1H, CH_2), 1.84 (d, ^2J = 12.5 Hz, 1H, CH_2), 1.53 (s, 3H, N(CH_3)$_3$), 1.49 (d, ^3J = 6.4 Hz, 3H, *i*Pr-CH_3), 1.24 (d, ^3J = 6.7 Hz, 3H, *i*Pr-CH_3), 1.15 (s, 3H, N(CH_3)$_3$), 1.05 (d, ^3J = 6.7 Hz, 3H, *i*Pr-CH_3), 1.00 (s, 3H, C(CH_3)$_2$), 0.92 (s, 3H, C(CH_3)$_2$), 0.59 (d, ^3J = 6.4 Hz, 3H, *i*Pr-CH_3) ppm.^{13}C{^{1}H} NMR (126 MHz, $CDCl_3$, 297 K): δ = 156.2

(*p*-DMAP-*C*), 155.9 (*p*-DMAP-*C*), 151.9 (*o*-Ar*C*), 149.2 (*o*-Ar*C*), 146.5 (*i*-Ar*C*), 144.8 (*o*-DMAP-*C*), 144.5 (*o*-DMAP-*C*), 126.2 (*p*-Ar*C*), 125.8 (*m*-Ar*C*), 124.1 (*m*-Ar*C*), 121.4 (q, $^{1}J_{13C-19F}$ = 321 Hz, CF_3), 107.5 (*m*-DMAP-*C*), 107.0 (*m*-DMAP-*C*), 67.9 (B*C*H), 63.3 (N*C*(CH_3)$_2$), 61.6 (*C*H_2), 41.6 (*C*(CH_3)$_2$), 39.9 (DMAP-*C*H_3), 39.9 (DMAP-*C*H_3), 33.8 (C(*C*H_3)$_2$), 33.2 (C(*C*H_3)$_2$), 29.0 (*i*Pr-*C*H), 28.5 (C(*C*H_3)$_2$), 28.4 (C(*C*H_3)$_2$), 27.3 (*i*Pr-*C*H), 27.1 (*i*Pr-*C*H_3), 25.9 (*i*Pr-*C*H_3), 25.0 (*i*Pr-*C*H_3), 24.0 (*i*Pr-*C*H_3) ppm. ^{11}B NMR (160 MHz, $CDCl_3$, 297 K): δ = 4.2 (broad) ppm. ^{19}F NMR (471 MHz, $CDCl_3$, 297 K): δ = –78.9 (s) ppm. HRMS LIFDI for $[C_{34}H_{53}BN_5]^+$ = $[M – SO_3CF_3]^+$: calcd. 542.4389; found 542.4363; $[C_{27}H_{43}BN_3]^+$ = $[M – SO_3CF_3 – DMAP]^+$: calcd. 420.3545; found 420.3525.

[(CAAC^{Me}H)BH(Pyr)$_2$]OTf, 3-Pyr

Compound **1** (20.0 mg, 45 µmol) were dissolved in pyridine. ^{11}B NMR data recorded after 15 min at rt show a ca. 1:1 mixture of **2-Pyr** (δ_{11B} = –9.1 ppm) as well as a second species (δ_{11B} = 6.9 ppm, very broad) attributable to the pyridine analogue of **3-DMAP**, [(CAAC^{Me}H)BH(Pyr)$_2$]OTf (**3-Pyr**), by analogy. Heating at 80 °C for 1 day resulted in a maximum conversion of ca. 75% to **3-Pyr**. Attempts to isolate this compound failed as removal of the pyridine solvent also results in removal of the second adducted pyridine ligand and isolation of **2-Pyr**.

[(CAAC^{Me})BH$_2$(4,4'-bipyridine)BH$_2$(CAAC^{Me})](OTf)$_2$, 4-Bipy

To a solution of **1** (200 mg, 0.45 mmol, 2.0 eq.) in benzene (4 mL) 4,4'-bipyridine (35.0 mg, 0.22 mmol) dissolved in benzene (1 mL) was added dropwise. The reaction mixture instantly turned red accompanied by the formation of a precipitate. After stirring for 1 d at room temperature the suspension was filtered. The pink solid was washed with benzene and hexanes and dried *in vacuo* to afford **4-Bipy** (196 mg, 0.18 mmol, 85% yield). ^{1}H{^{11}B} NMR (500 MHz, $CDCl_3$, 297 K): δ = 8.79 (d, ^{3}J = 6.9 Hz, 4H, *o*-Bipy*H*), 8.70 (d, ^{3}J = 6.9 Hz, 4H, *m*-Bipy*H*), 7.48 (t, ^{3}J = 7.7 Hz, 2H, *p*-Ar*H*), 7.33 (d, ^{3}J = 7.6 Hz, 4H, *m*-Ar*H*), 2.96 (s, 4H, B*H*$_2$), 2.69 (sept, ^{3}J = 6.6 Hz, 4H, *i*Pr-C*H*), 2.16 (s, 4H, C*H*$_2$), 1.41 (s, 12H, NC(C*H*$_3$)$_2$), 1.39 (d, ^{3}J = 6.8 Hz, 12H, *i*Pr-C*H*$_3$), 1.32 (d, ^{3}J = 6.5 Hz, 12H, *i*Pr-C*H*$_3$), 1.23 (s, 12H, C(C*H*$_3$)$_2$) ppm. ^{13}C{^{1}H} NMR (126 MHz, $CDCl_3$, 297 K): δ = 224.7 (CAAC-$C_{carbene}$), 150.0 (*m*-Bipy-*C*), 147.8 (*p*-Bipy-*C*), 144.5 (*o*-Ar*C*), 131.3 (*i*-Ar*C*), 130.5 (*p*-Ar*C*), 126.4 (*o*-Bipy-*C*), 125.5 (*m*-Ar*C*), 121.0 (q, $^{1}J_{13C-19F}$ = 320 Hz, *C*F_3), 79.7 (N*C*(CH_3)$_2$), 53.4 (*C*(CH_3)$_2$), 51.3 (*C*H_2), 29.4 (*i*Pr-*C*H), 29.2 (C(*C*H_3)$_2$), 29.0 (NC(*C*H_3)$_2$), 26.4 (*i*Pr-*C*H_3), 23.9 (*i*Pr-*C*H_3) ppm. ^{11}B NMR (160 MHz, $CDCl_3$, 297 K): δ = –8.6 (very broad) ppm. ^{19}F NMR (471 MHz, $CDCl_3$, 297 K): δ = –78.2 (s) ppm.

Elemental analysis for [$C_{52}H_{74}B_2F_6N_4O_6S_2$] ($M_w$ = 1050.92): calcd. C 59.43, H 7.10, N 5.33, S 6.10%; found C 58.33, H 6.97, N 5.35, S 5.97%.

[(CAAC^{Me})BH(CAAC^{Me}H)]K, 5

a) Compound **2-CAAC^{Me}** (20.0 mg, 27.3 μmol) and KC_8 (8.0 mg, 60.0 μmol, 2.2 eq.) were suspended in 0.6 mL of C_6D_6. After 1 min the suspension was filtered yielding an intense red solution of **5** in analytically pure quality sufficient for NMR spectroscopic analysis. The solution was concentrated *in vacuo* yielding bright orange single crystals of **5** after 1 d at rt. *Note: Evaporation of the solvent in vacuo resulted in some hydrolysis to the known compound [(CAAC^{Me})BH$_2$(CAAC^{Me}H)].*[2]

b) **2-CAAC^{Me}** (100 mg, 0.14 mmol) and KC_8 (40.0 mg, 0.30 mmol, 2.2 eq.) were suspended in 3 mL of hexane for one minute prior to filtration. Complete evaporation of the solvent under atmospheric pressure afforded **7** as a red solid in essentially quantitative yield (86 mg, 0.13 mmol, 99% yield). ^{1}H{^{11}B} NMR (500 MHz, C_6D_6, 297 K): δ = 7.03–7.01 (m, 1H, Ar*H*), 6.84–6.80 (m, 2H, Ar*H*), 6.73–6.69 (m, 2H, Ar*H*), 6.67–6.64 (m, 1H, Ar*H*), 4.38 (d, ^{3}J = 6.6 Hz, 1H, BC*H*), 4.08 (sept, ^{3}J = 6.7 Hz, 1H, *i*Pr-C*H*), 3.83 (sept, ^{3}J = 6.8 Hz, 1H, *i*Pr-C*H*), 3.63 (sept, ^{3}J = 6.7 Hz, 1H, *i*Pr-C*H*), 3.37 (sept, ^{3}J = 6.8 Hz, 1H, *i*Pr-C*H*), 2.09 (d, ^{2}J = 11.7 Hz, 1H, CH_2), 2.02 (d, ^{2}J = 8.6 Hz, 1H, CH_2), 2.00 (s, 3H, NC(CH_3)$_2$), 1.96 (d, ^{2}J = 11.8 Hz, 1H, CH_2), 1.95 (d, ^{2}J = 11.5 Hz, 1H, CH_2), 1.90 (d , ^{3}J = 6.6 Hz, 1H, B*H*), 1.66 (s, 6H, NC(CH_3)$_2$), 1.48 (d, ^{3}J = 6.7 Hz, 3H, *i*Pr-CH_3), 1.47 (s, 3H, NC(CH_3)$_2$), 1.36 (s, 3H), 1.34–1.32 (m, 6H), 1.22 (d, ^{3}J = 6.8 Hz, 3H, *i*Pr-CH_3), 1.16–1.15 (m, 9H), 1.14–1.13 (m, 3H), 1.08–1.06 (m, 6H), 0.82 (d, ^{3}J = 7.1 Hz, 3H, *i*Pr-CH_3) ppm. ^{13}C{^{1}H} NMR (126 MHz, C_6D_6, 297 K): δ = 155.9, 155.7, 155.2, 150.1 ($C_{carbene}$), 149.7, 147.7, 145.5, 125.2, 124.8, 124.5, 123.4, 123.4, 123.2 (= 12 C Aryl), 67.1 (B*C*H), 62.0 (*C*H$_2$), 61.6 (*C*H$_2$), 61.2 (N*C*(CH$_3$)$_2$), 60.9 (N*C*(CH$_3$)$_2$), 43.5, 43.1, 38.8, 37.9, 33.1, 32.0, 32.0, 29.1, 28.3, 28.2, 27.9, 27.6, 27.5, 27.2, 27.1, 26.5, 26.2, 26.1, 25.3, 25.2, 24.8 ppm. ^{11}B NMR (160 MHz, C_6D_6, 297 K): δ = 16.7 (broad) ppm. Solid-state IR: ν(B–H) = 2329 cm^{-1}. Elemental analysis for [$C_{40}H_{64}BKN_2$] (M_w = 622.87): calcd. C 77.13, H 10.36, N 4.50%; found C 77.35, H 11.06, N 4.36%.

[(CAAC^{Me})BH(CAAC^{Me}H)]K(thf)$_3$, 5-thf

2-CAAC^{Me} (50.0 mg, 54.6 μmol) and KC_8 (20.0 mg, 15.0 μmol, 2.2 eq.) were suspended in 3 mL of a 1:5 THF/hexane solvent mixture for one minute prior to filtration. The filtrate was stored at –25 °C for several days providing a crop of red crystals suitable for X-ray structural analysis (25.0 mg, 29.8 μmol, 55% yield). ^{1}H NMR (400 MHz, C_6D_6, 297 K): δ = 7.02 (d,

^3J = 7.5 Hz, 1H, Ar*H*), 6.86 (d, ^3J = 7.5 Hz, 1H, Ar*H*), 6.81 (t, ^3J = 7.8 Hz, 1H, Ar*H*), 6.75–6.72 (m, 2H, Ar*H*), 6.69 (t, ^3J = 7.6 Hz, 1H, Ar*H*), 4.39 (d, ^3J = 6.6 Hz, 1H, BC*H*), 4.11 (sept, ^3J = 6.7 Hz, 1H, *i*Pr-C*H*), 3.84 (sept, ^3J = 6.8 Hz, 1H, *i*Pr-C*H*), 3.65 (sept, ^3J = 6.7 Hz, 1H, *i*Pr-C*H*), 3.55 (m, 12H, THF-OCH_2), 3.43 (sept, ^3J = 6.8 Hz, 1H, *i*Pr-C*H*), 2.10 (d, ^2J = 11.8 Hz, 1H, CH_2), 2.04 (d, ^2J = 8.6 Hz, 1H, CH_2), 2.00 (s, 3H, NC(CH_3)$_2$), 1.98–1.93 (m, 2H, CH_2), 1.66 (s, 3H, NC(CH_3)$_2$), 1.65 (s, 3H, NC(CH_3)$_2$), 1.50 (d, ^3J = 6.7 Hz, 3H, *i*Pr-CH_3), 1.46 (s, 3H, NC(CH_3)$_2$), 1.43–1.39 (m, 12H, THF-CH_2), 1.36–1.35 (m, 6H), 1.32 (s, 3H), 1.22 (d, ^3J = 6.7 Hz, 3H, *i*Pr-CH_3), 1.17–1.13 (m, 12H), 1.11–1.09 (m, 6H), 0.85 (d, ^3J = 6.9 Hz, 3H, *i*Pr-CH_3) ppm. ^{11}B NMR (128 MHz, C_6D_6, 297 K): δ = 16.8 ppm. Solid-state IR: ν(B–H) = 2269 cm^{-1}. Elemental analysis for [$C_{52}H_{88}BKN_2O_3$] (M_w = 839.19): calcd. C 74.43, H 10.57, N 3.34%; found C 74.54, H 10.33, N 3.87%.

[(CAAC^{Me})BH(CAAC^{Me}H)]$^•$, 6

A solution of **5-thf** (100 mg, 0.16 mmol) in 5 mL of THF was added dropwise to a solution of **2-CAAC^{Me}** (117 mg, 0.16 mmol) in 3 mL of THF. The reaction mixture instantly turned intense purple and a colorless precipitate was formed. The ^{11}B NMR spectrum of the filtrate was silent indicating the presence of a radical species. After evaporation of the solvent under atmospheric pressure the product was extracted with hexane. Complete removal of hexane under atmospheric pressure afforded **6** as bright orange crystals (166 mg, 0.28 mmol, 89% yield) suitable for X-ray structure analysis. Solid state IR: ν(B–H) = 2533 cm^{-1}. Elemental analysis for [$C_{40}H_{64}BN_2$] (M_w = 583.78): calcd. C 82.30, H 11.05, N 4.80%; found C 82.08, H 11.35, N 4.75%.

[(CAAC^{Me})$_2$BH$_2$](S$_n$)$_{0.5}$, 7:

a) To a solution of **5** (100 mg, 0.16 mmol) in 3 mL of benzene elemental sulfur (33 mg, 1.28 mmol, 64.0 eq.) was added. The reaction mixture turned dark orange accompanied by formation of precipitate. After stirring for 2 h at room temperature the suspension was filtered. The residue was washed with benzene and hexane affording **7** as an off-white solid (104 mg, 0.12 mmol, 72%). The ^{1}H and ^{11}B NMR spectra of **7** were essentially identical with those of **2-CAAC^{Me}**, indicating the formation of the [(CAAC^{Me})$_2$BH$_2$]$^+$ cation, presumably with a S_n^{2-} counteranion. ^{1}H{^{11}B} NMR (500 MHz, $CDCl_3$, 297 K): δ = 7.27 (t, ^3J = 7.7 Hz, 2H, *p*-Ar*H*), 7.24–7.18 (m, 2H, *m*-Ar*H*), 7.07–7.02 (m, 2H, *m*-Ar*H*), 2.57 (br s, 2H, *i*Pr-C*H*), 2.43 (d, ^2J = 12.0 Hz, 2H, CH_2), 2.04 (d, ^2J = 11.4 Hz, 2H, CH_2), 1.93 (br s, 8H, two *i*Pr-C*H* and

$NC(CH_3)_2$), 1.70 (s, 2H, BH_2), 1.57 (s, 6H, $NC(CH_3)_2$), 1.49 (s, 6H, $C(CH_3)_2$), 1.33 (s, 6H, *i*Pr-CH_3), 1.14 (br s, 18H, $C(CH_3)_2$ and two *i*Pr-CH_3), 0.17 (s, 6H, *i*Pr-CH_3) ppm. $^{13}C\{^1H\}$ NMR (126 MHz, $CDCl_3$, 297 K): δ = 192.7 ($C_{carbenes}$), 144.8 (*i*-Ar*C*), 142.6 (*i*-Ar*C*), 132.7 (*o*-Ar*C*), 129.8 (*p*-Ar*C*), 125.9 (*m*-Ar*C*), 79.3 ($NC(CH_3)_2$), 53.7 (CH_2), 52.0 ($C(CH_3)_2$), 29.9 (*i*Pr-*C*H), 29.4 ($NC(CH_3)_2$), 29.2 ($C(CH_3)_2$), 28.9 ($C(CH_3)_2$), 28.7 ($NC(CH_3)_2$), 27.2 (*i*Pr-CH_3), 25.7 (*i*Pr-CH_3), 24.5 (*i*Pr-CH_3), 24.3 (*i*Pr-CH_3) ppm. ^{11}B NMR (160 MHz, $CDCl_3$, 297 K): δ = –22.4 (t, $^1J_{11B\text{-}1H}$ = 84.7 Hz) ppm. HRMS LIFDI-pos. for $[C_{40}H_{64}BN_2]^+$ = $[M]^+$: calcd. 583.5157; found 583.5140.

$(CAAC^{Me}H)_2B(CH_3)$, 8:

2-CAAC^{Me} (20.0 mg, 27.3 μmol) and KC_8 (8.0 mg, 60.0 μmol, 2.2 eq.) were suspended in 1 mL of hexane for 1 min prior to filtration. The red filtrate was treated with MeOTf (4.5 mg, 27.3 μmol) whereupon the mixture instantly turned colorless accompanied by formation of a precipitate. After filtration and evaporation of the solvent *in vacuo*, the residue was dissolved in 0.6 mL of C_6D_6 and NMR-spectroscopic analysis showed **8** in analytically pure quality. After freeze-drying **7** was obtained as a colorless solid (13 mg, 21.7 μmol, 80% yield). ^1H NMR (500 MHz, C_6D_6, 297 K): δ = 7.14–7.07 (m, 6H, Ar*H*), 4.35 (sept, ^3J = 6.8 Hz, 2H, *i*Pr-C*H*), 3.65 (s, 2H, BC*H*), 3.23 (sept, ^3J = 6.7 Hz, 2H, *i*Pr-C*H*), 2.09 (d, ^2J = 12.6 Hz, 2H, CH_2), 1.66 (s, 3H, BCH_3), 1.57 (s, 6H, $NC(CH_3)_2$), 1.54 (d, ^3J = 6.7 Hz, 6H, *i*Pr-CH_3), 1.40 (d, ^2J = 12.8 Hz, 2H, CH_2), 1.37 (d, ^3J = 6.9 Hz, 6H, *i*Pr-CH_3), 1.23 (d, ^3J = 7.0 Hz, 6H, *i*Pr-CH_3), 1.19 (s, 6H, $NC(CH_3)_2$), 1.16 (d, ^3J = 6.7 Hz, 6H, *i*Pr-CH_3), 0.98 (s, 6H, $C(CH_3)_2$), 0.58 (s, 6H, $C(CH_3)_2$) ppm. $^{13}C\{^1H\}$ NMR (126 MHz, C_6D_6, 297 K): δ = 151.3 (*o*-Ar*C*), 149.1 (*o*-Ar*C*), 146.6 (*i*-Ar*C*), 126.3 (*m*-Ar*C*), 125.9 (*m*-Ar*C*), 124.2 (*p*-Ar*C*), 77.5 (B*C*H), 64.2 ($NC(CH_3)_2$), 60.8 (CH_2), 42.5 ($C(CH_3)_2$), 34.2 ($NC(CH_3)_2$), 33.1 ($NC(CH_3)_2$), 29.9 ($C(CH_3)_2$), 28.7 (*i*Pr-*C*H), 28.2 ($C(CH_3)_2$), 27.5 (*i*Pr-*C*H), 27.0 (*i*Pr-CH_3), 26.6 (*i*Pr-CH_3), 24.7 (*i*Pr-CH_3), 24.7 (*i*Pr-CH_3), 14.1 (BCH_3) ppm. ^{11}B NMR (160 MHz, C_6D_6, 297 K): δ = 93.9 (very broad) ppm. Elemental analysis for $[C_{41}H_{67}BN_2]$ (M_w = 598.81): calcd. C 82.24, H 11.28, N 4.68%; found C 82.52, H 11.67, N 4.92%. HRMS ASAP pos for $[C_{41}H_{68}BN_2]$ = [M + H]: calcd. 599.5470; found 599.5468.

NMR spectra of isolated compounds

Figure S1. $^{1}H\{^{11}B\}$ NMR spectrum of **1** in C_6D_6.

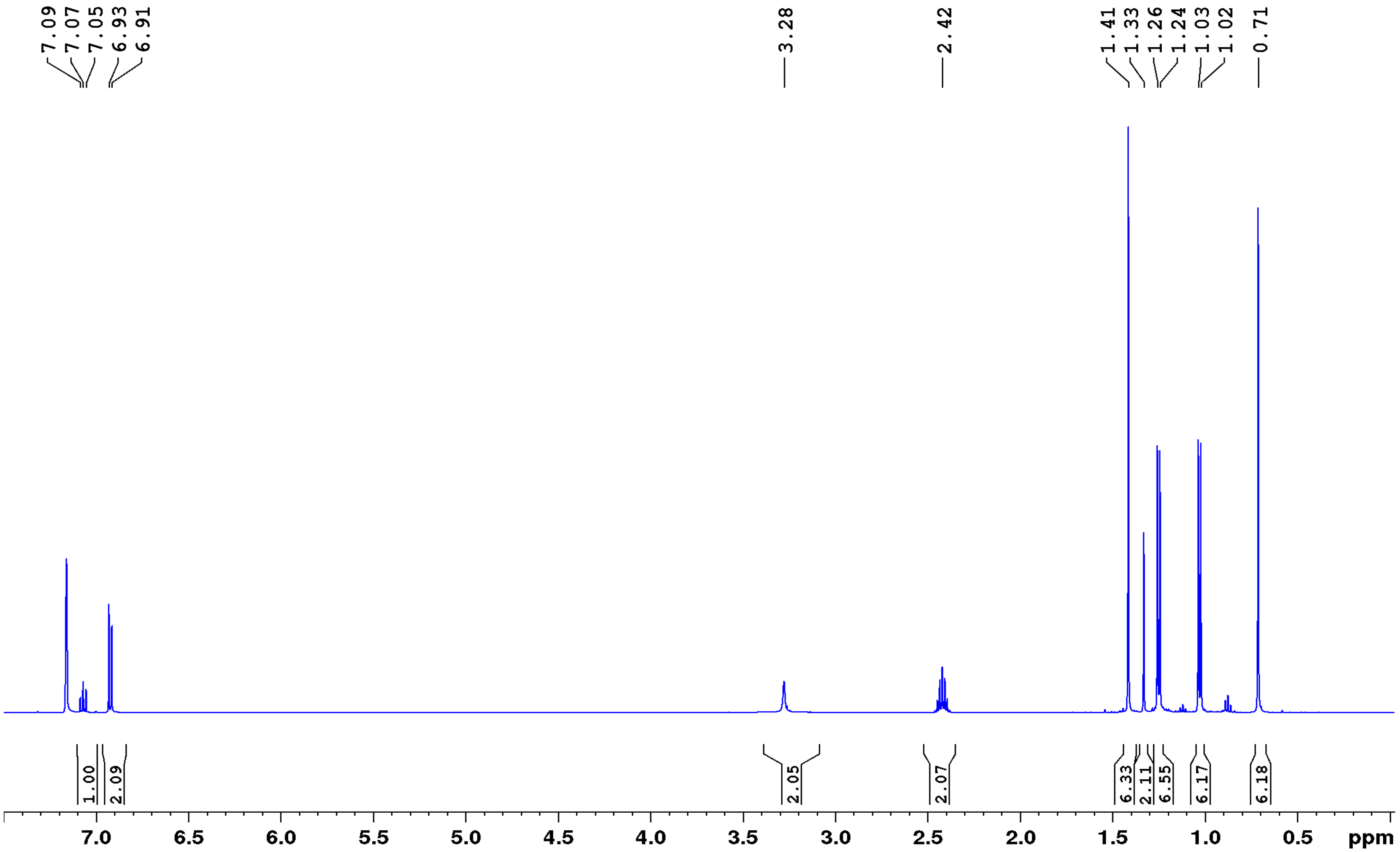

Figure S2. $^{13}C\{^{1}H\}$ NMR spectrum of **1** in C_6D_6.

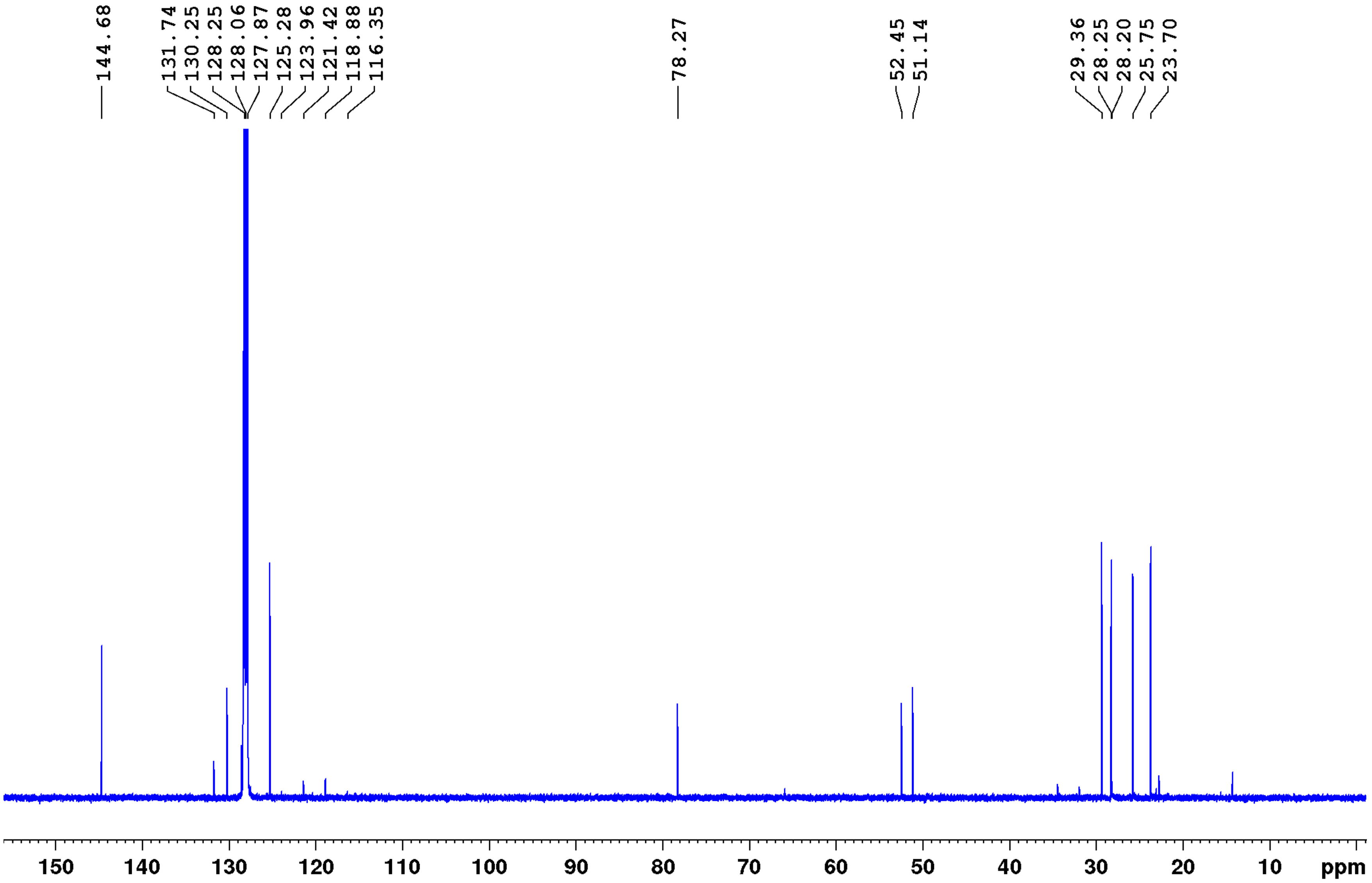

Figure S3. ^{11}B NMR spectrum of **1** in C_6D_6.

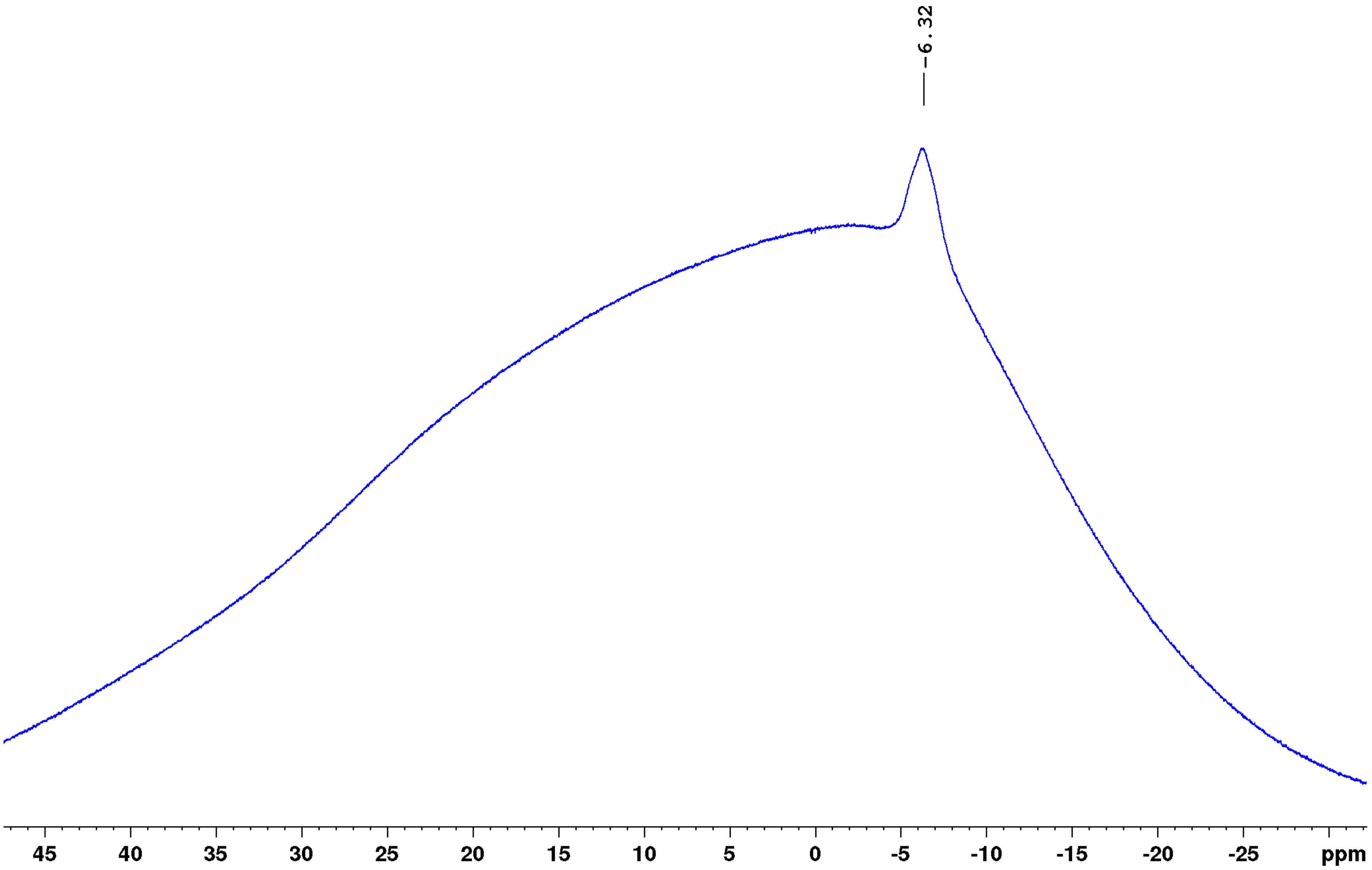

Figure S4. ^{19}F NMR spectrum of **1** in C_6D_6.

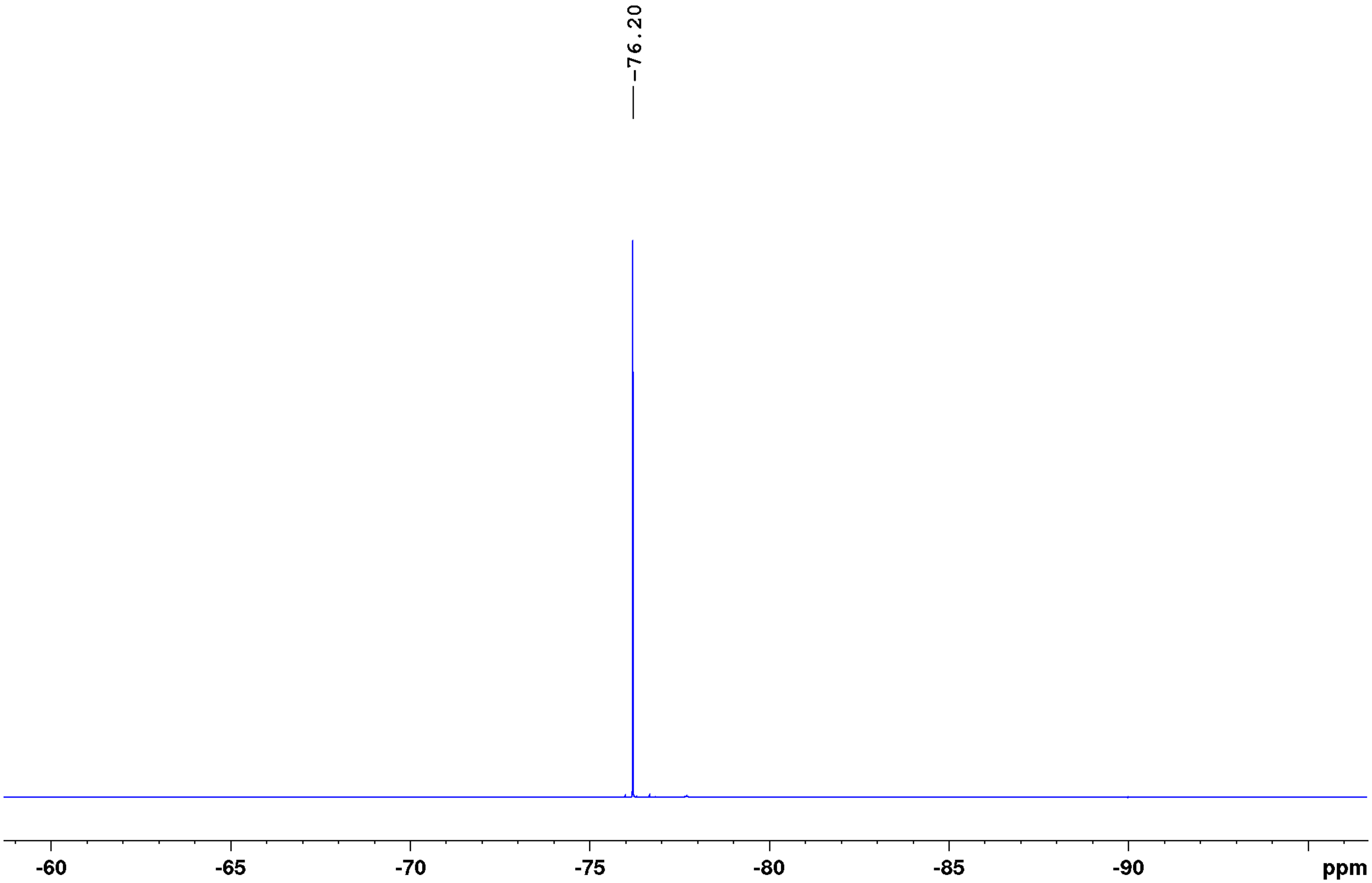

Figure S5. $^{1}H\{^{11}B\}$ NMR spectrum of **2-CAAC^{Me}** in $CDCl_3$ at rt.

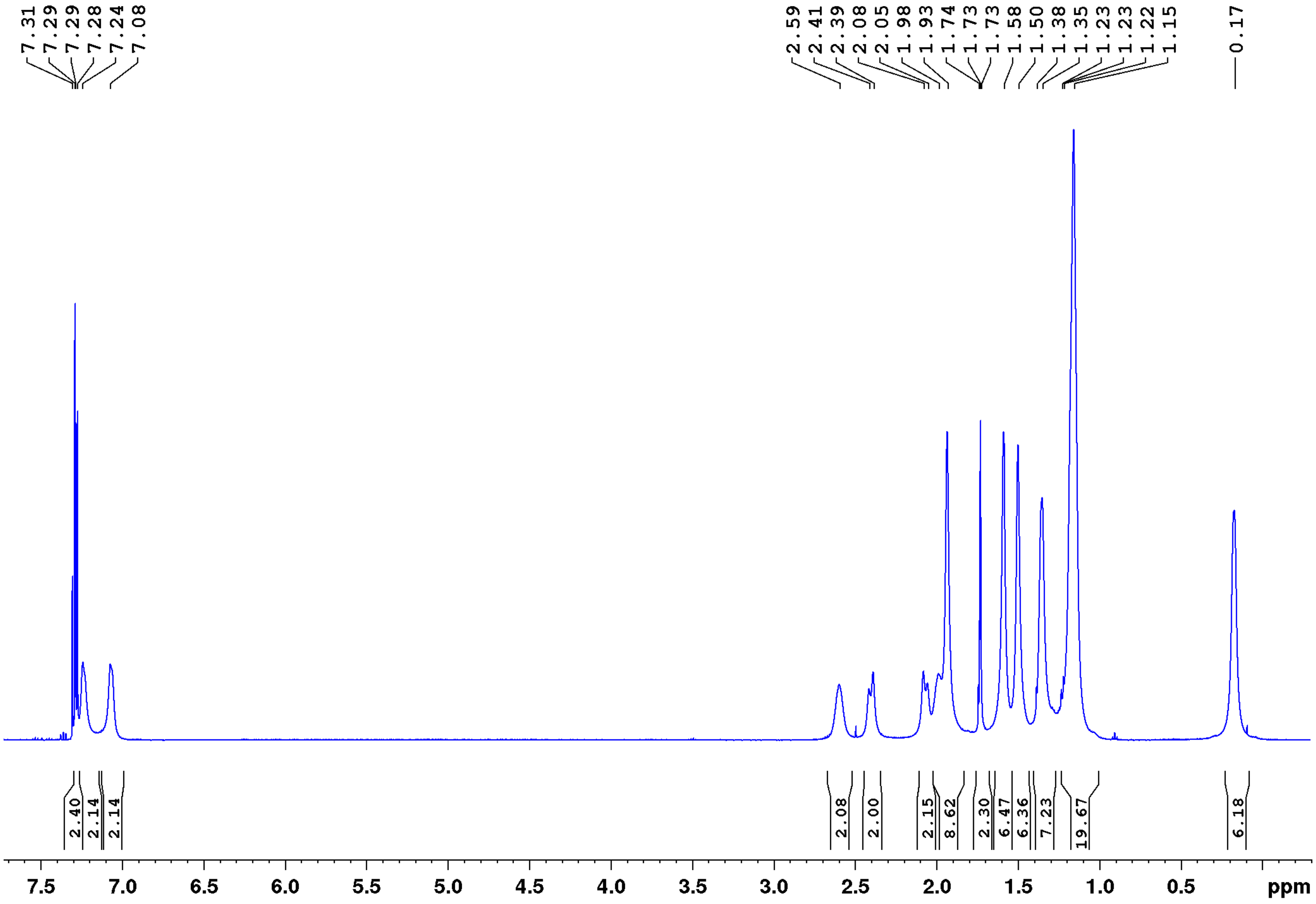

Figure S6. ^{1}H NMR spectrum of **2-CAAC^{Me}** in $CDCl_3$ at –55 °C.

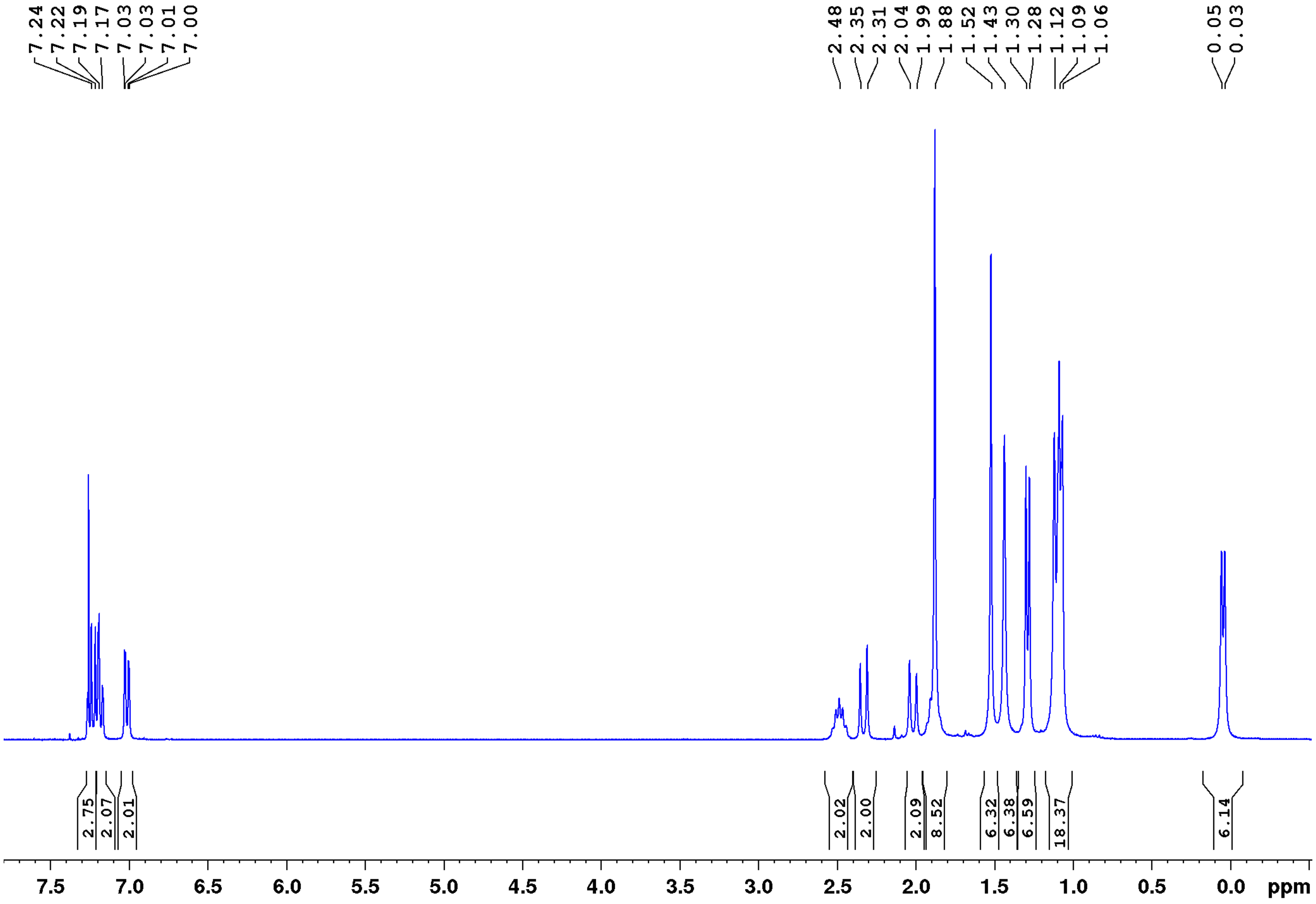

Figure S7. Stack-plot of $^{1}H\{^{11}B\}$ NMR spectra of **2-CAAC^{Me}** in $CDCl_3$ from –15 to +55 °C.

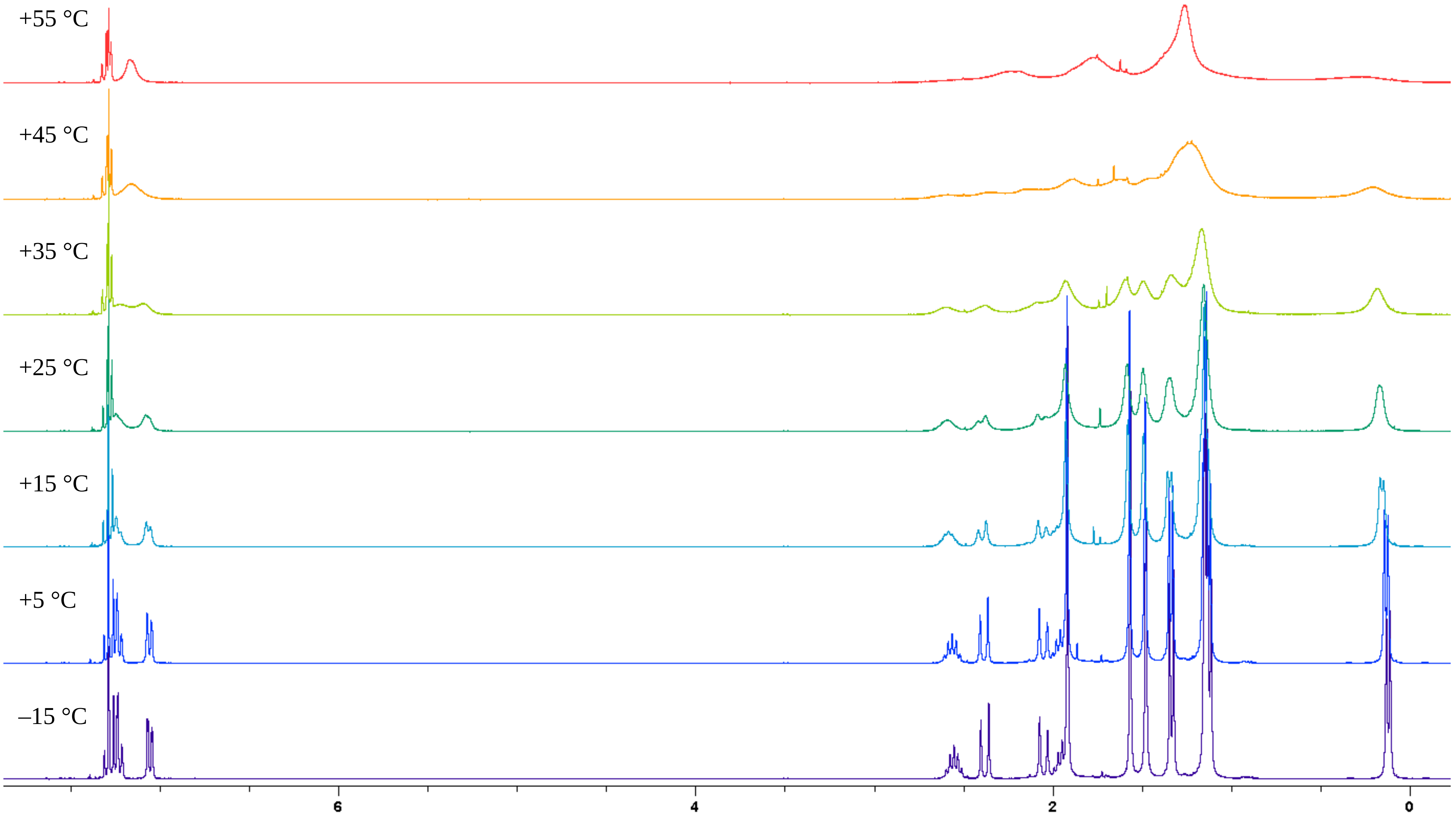

Figure S8. $^{13}C\{^{1}H\}$ NMR spectrum of **2-CAAC^{Me}** in $CDCl_3$.

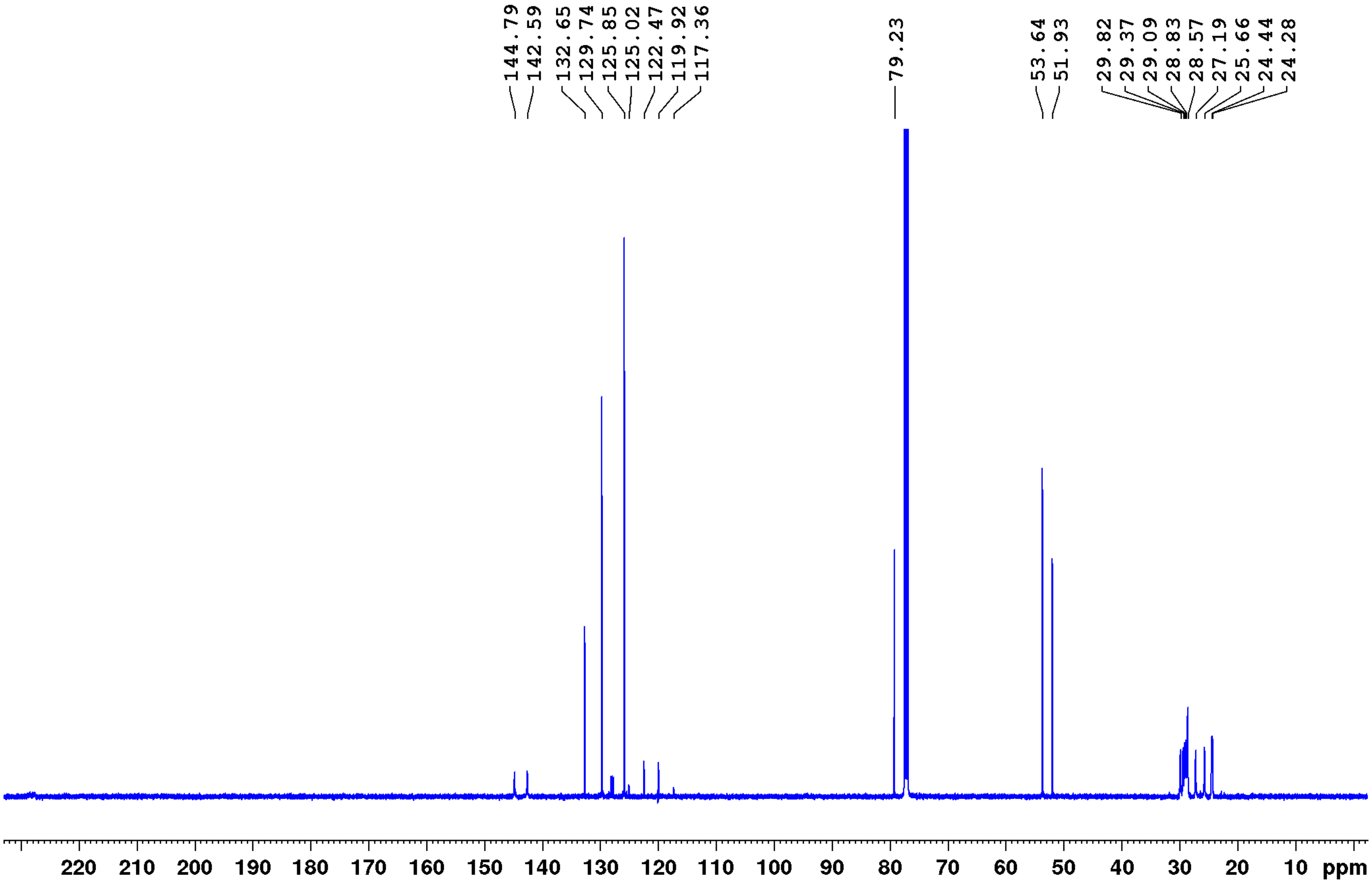

Figure S9. ^{11}B NMR spectrum of **2-CAAC^{Me}** in $CDCl_3$.

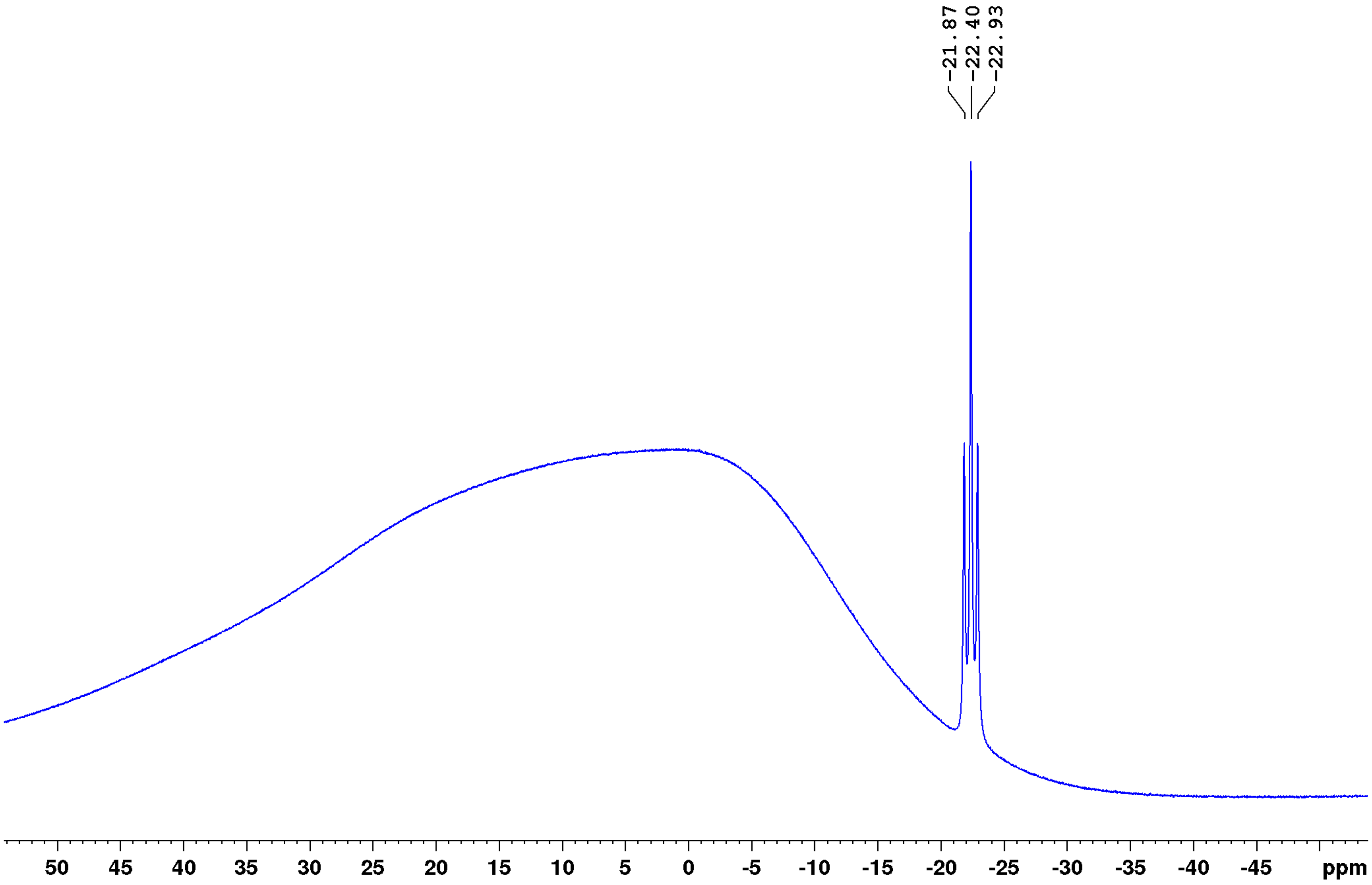

Figure S10. ^{19}F NMR spectrum of **2-CAAC^{Me}** in $CDCl_3$.

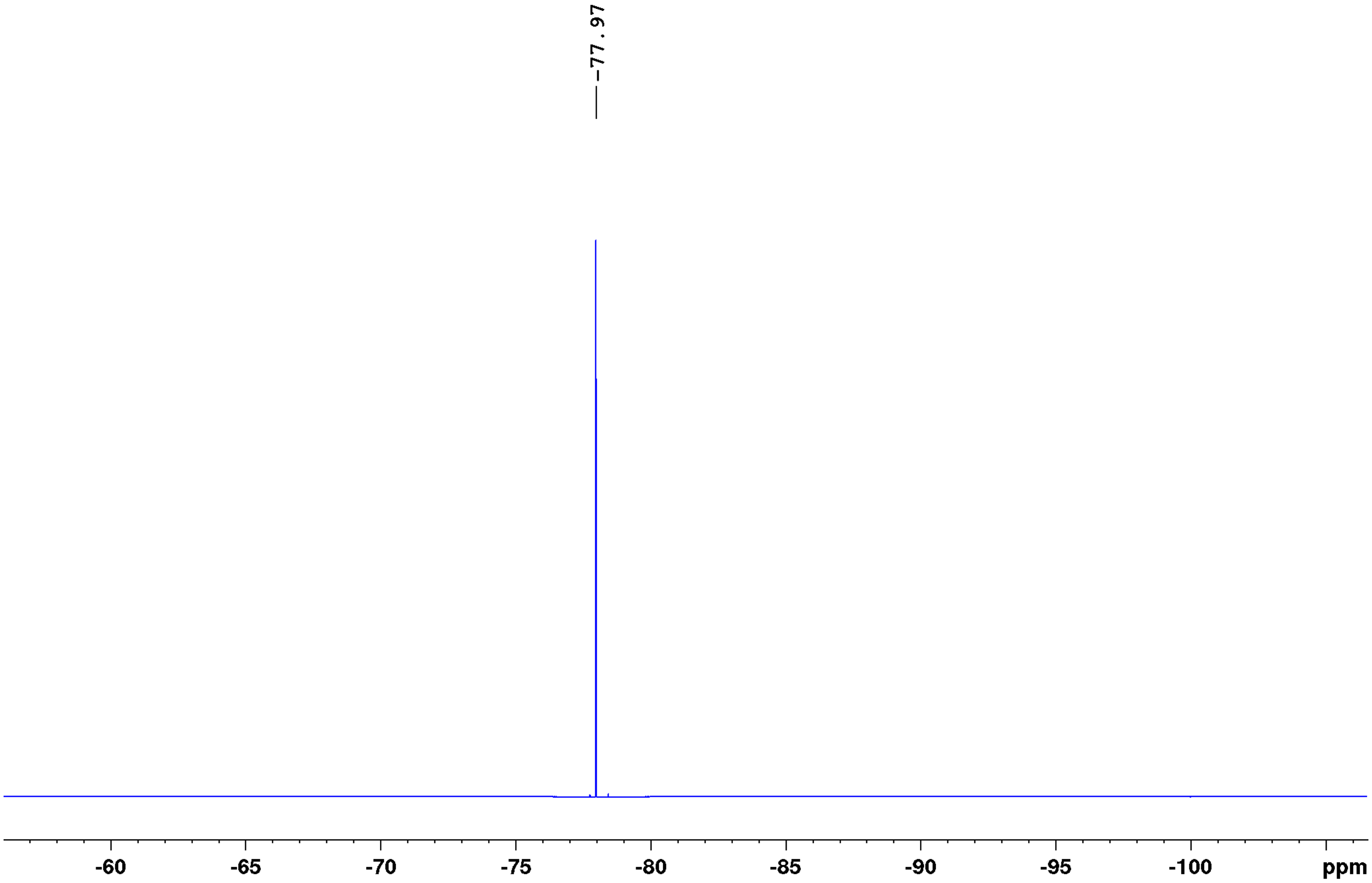

Figure S11. $^{1}H\{^{11}B\}$ NMR spectrum of **2-IMe^{Me}** in $CDCl_3$.

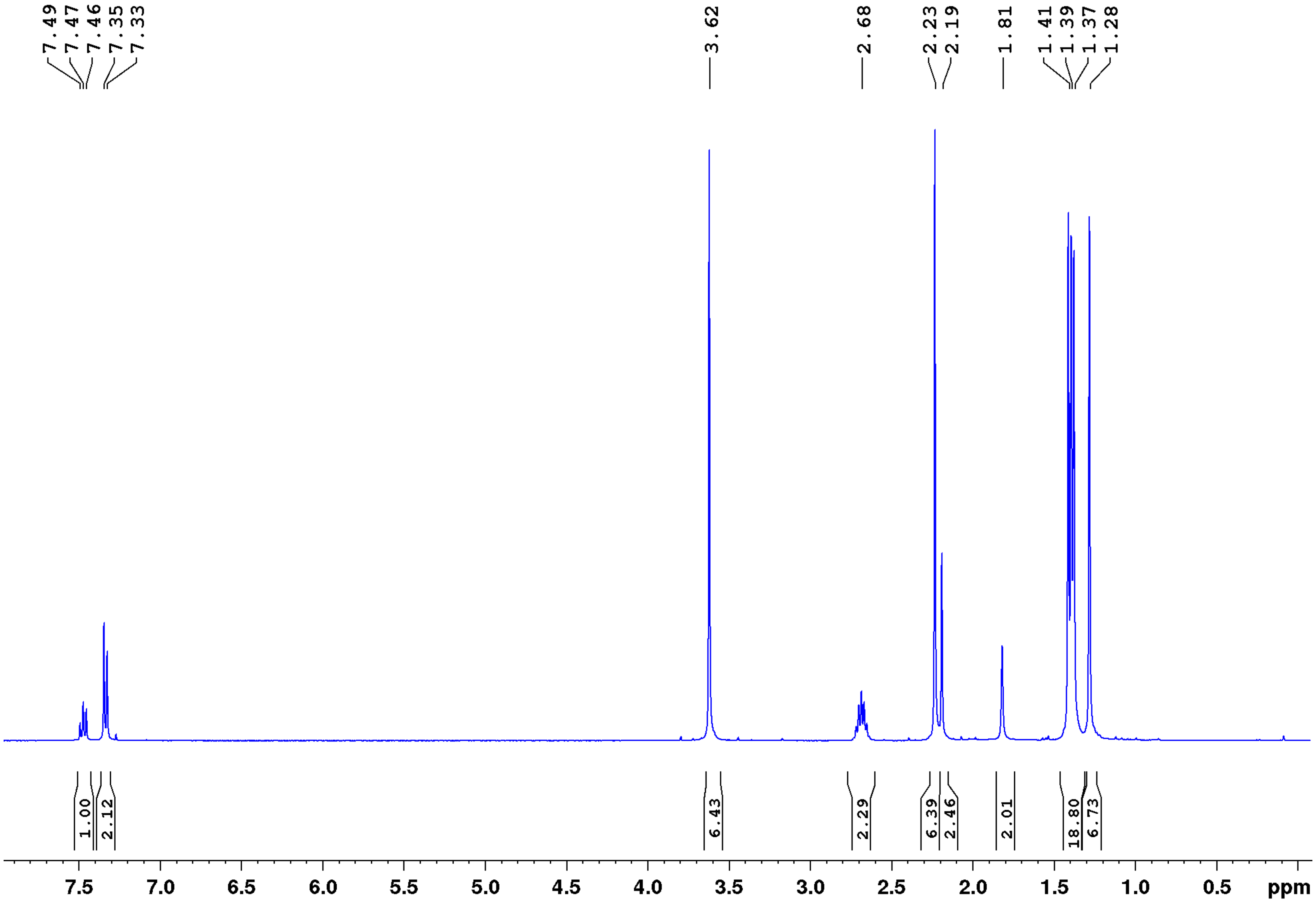

Figure S12. $^{13}C\{^{1}H\}$ NMR spectrum of **2-IMe^{Me}** in $CDCl_3$.

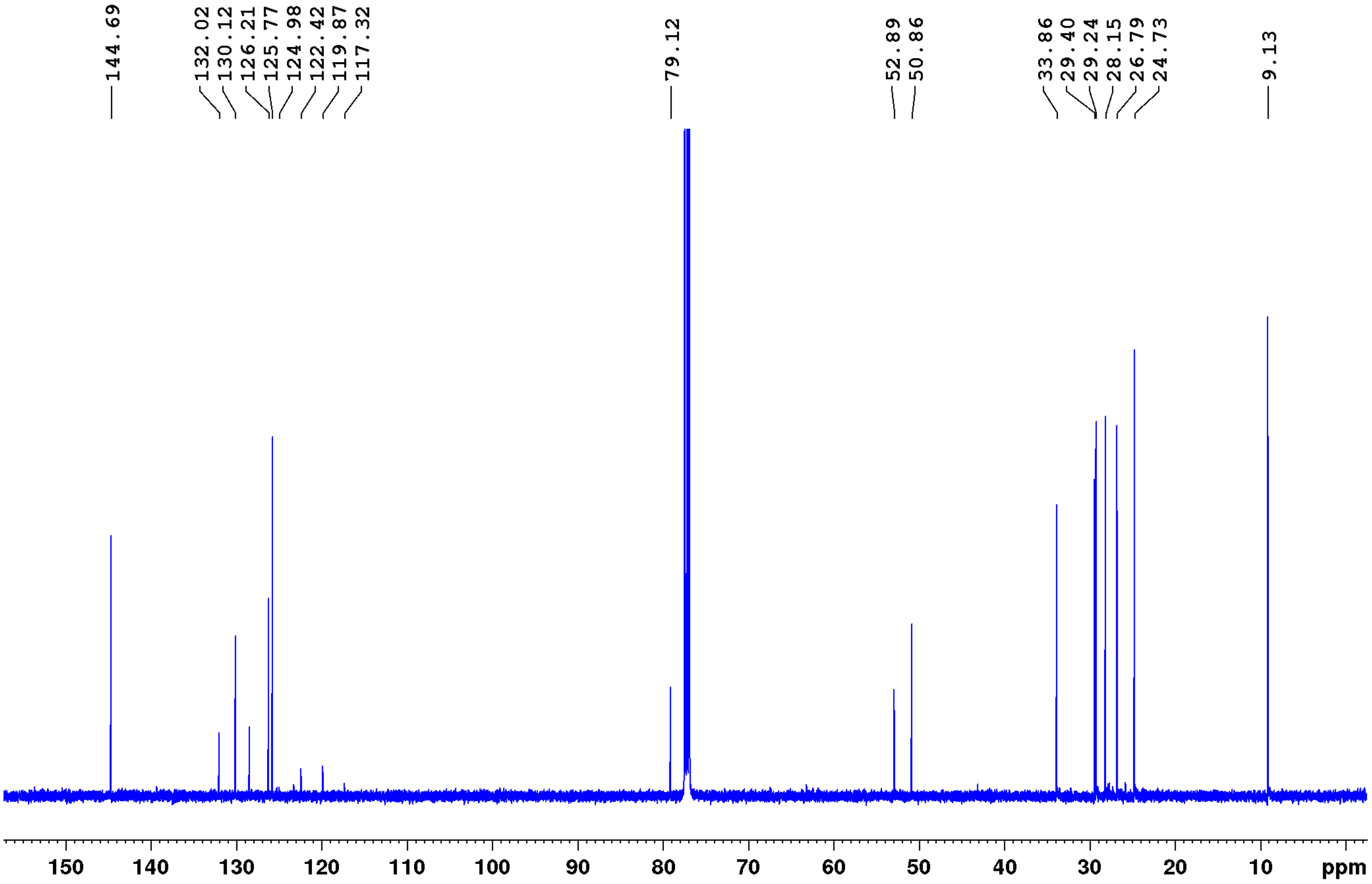

Figure S13. ^{11}B NMR spectrum of **2-IMe^{Me}** in $CDCl_3$.

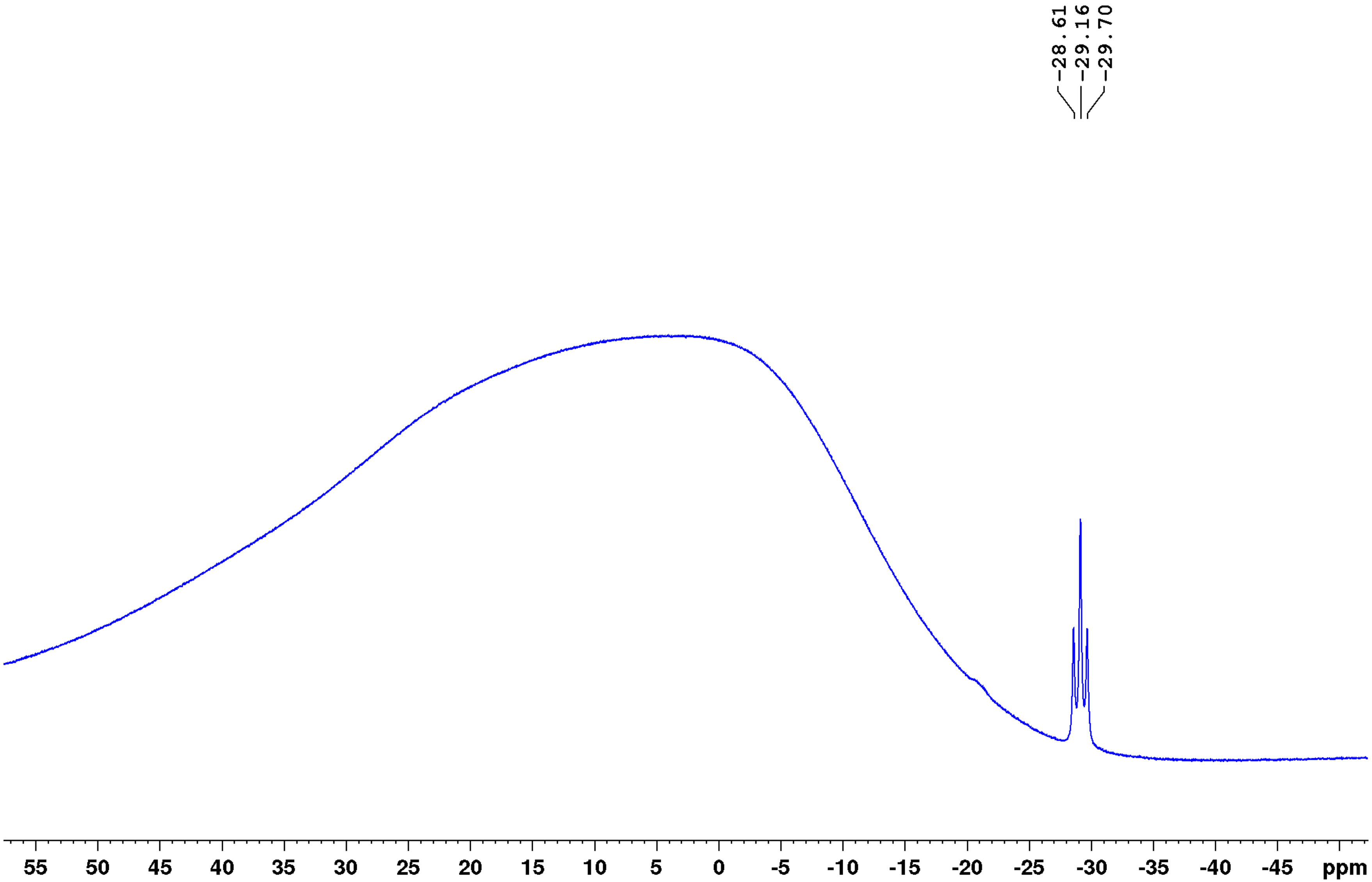

Figure S14. ^{19}F NMR spectrum of **2-IMe^{Me}** in $CDCl_3$.

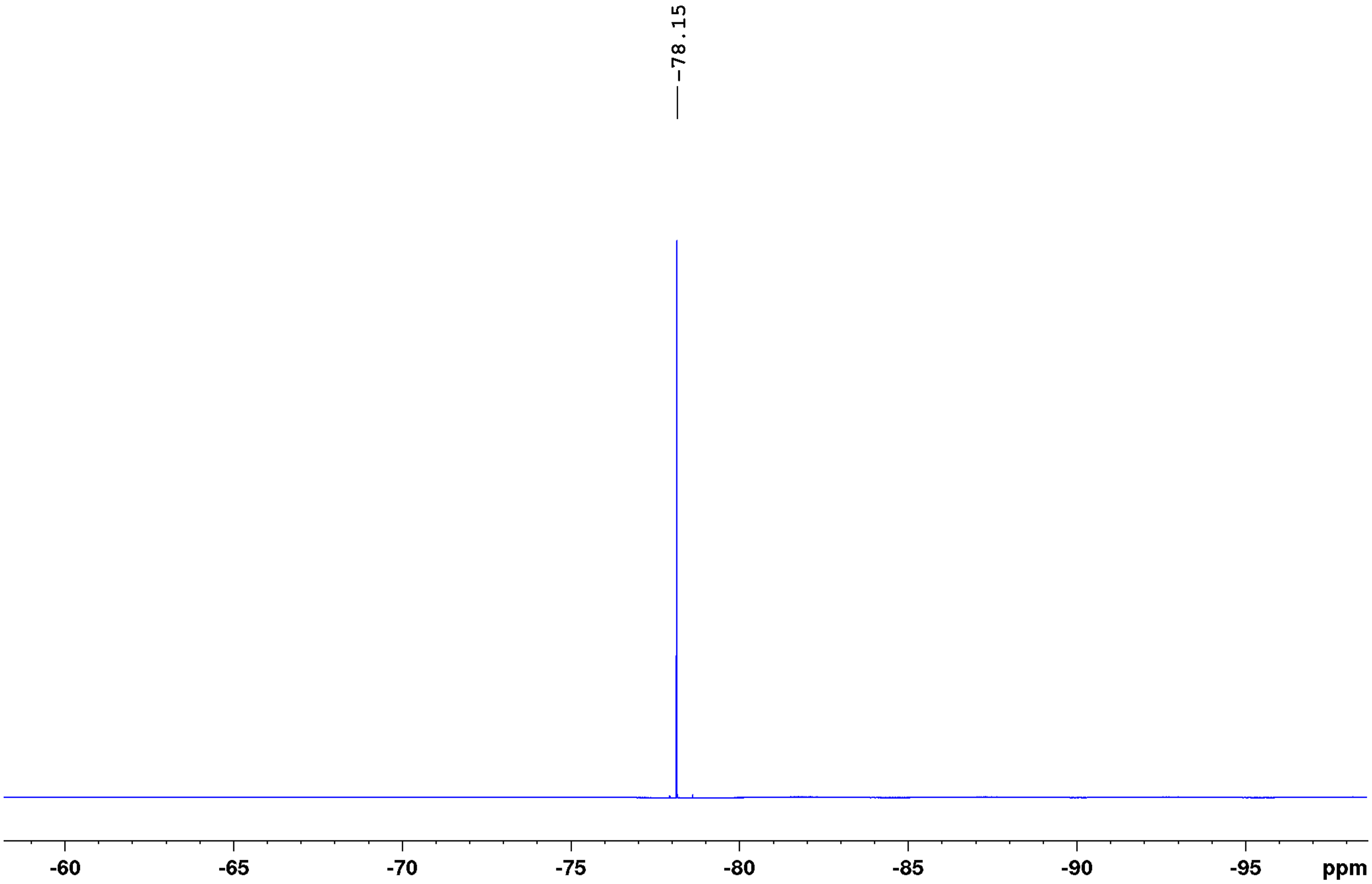

Figure S15. $^{1}H\{^{11}B\}$ NMR spectrum of **2-PMe$_3$** in CD_2Cl_2.

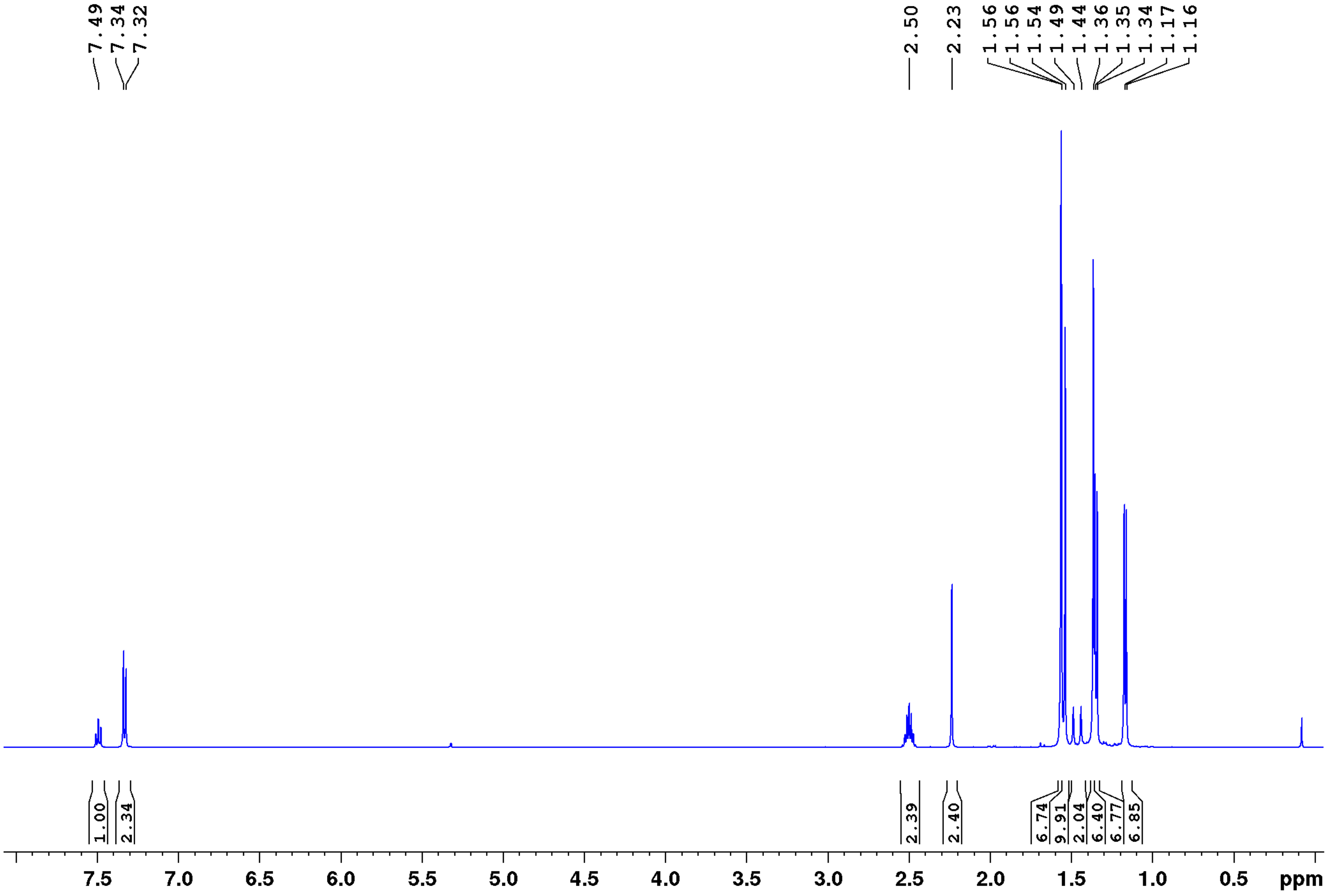

Figure S16. $^{13}C\{^{1}H\}$ NMR spectrum **2-PMe$_3$** in CD_2Cl_2.

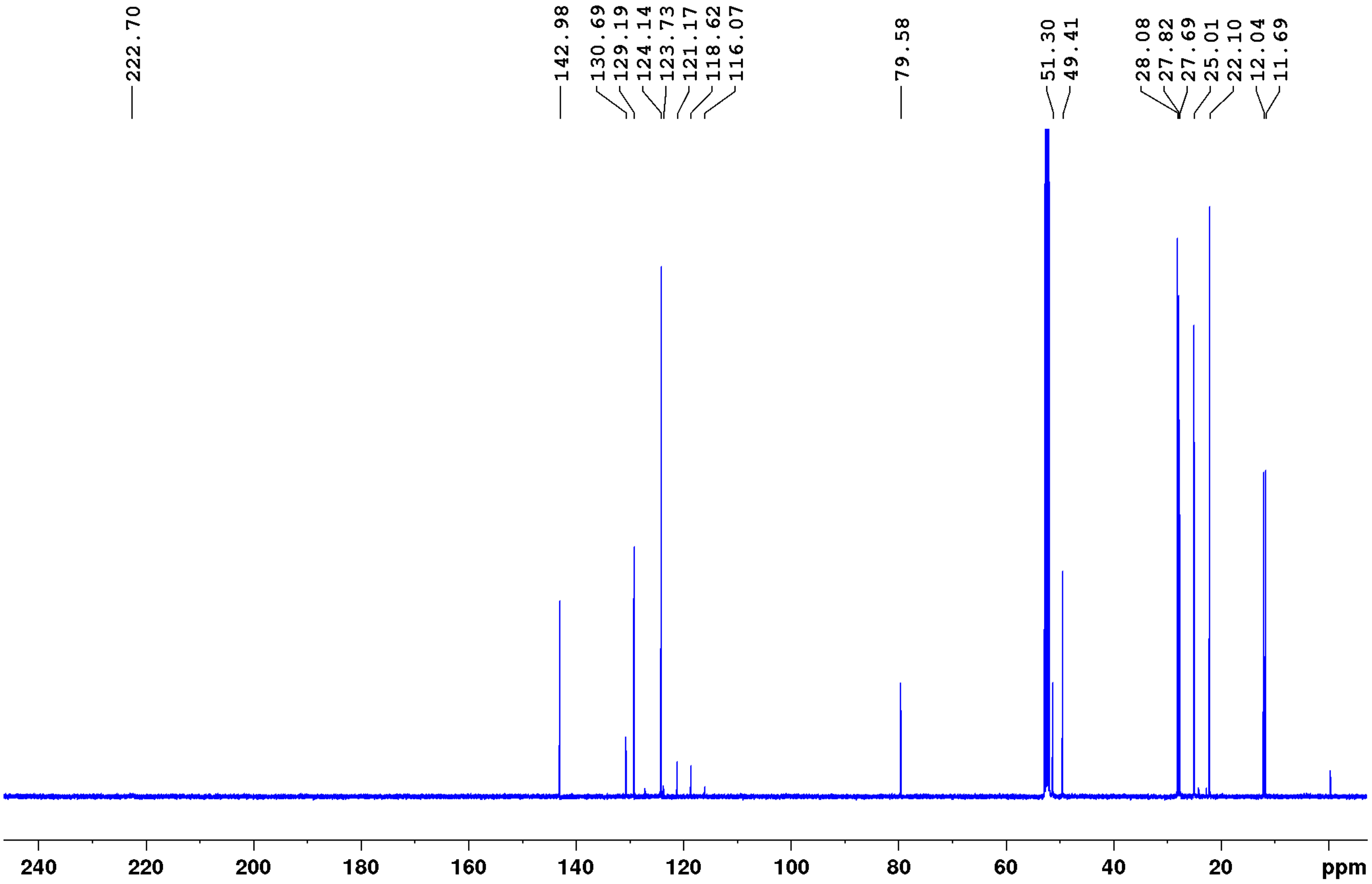

Figure S17. ^{11}B NMR spectrum of **2-PMe$_3$** in CD_2Cl_2.

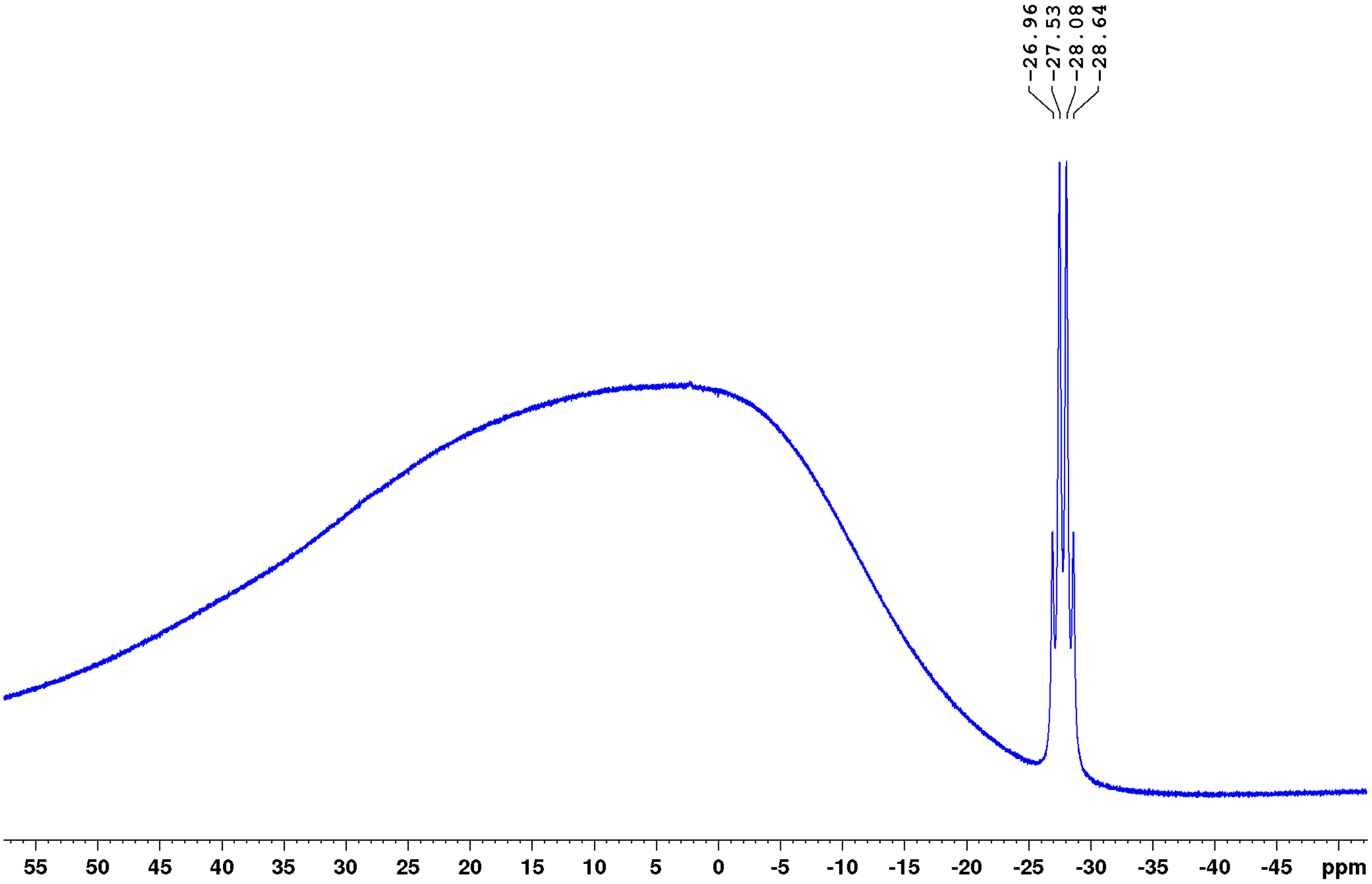

Figure S18. ^{19}F NMR spectrum of **2-PMe$_3$** in CD_2Cl_2.

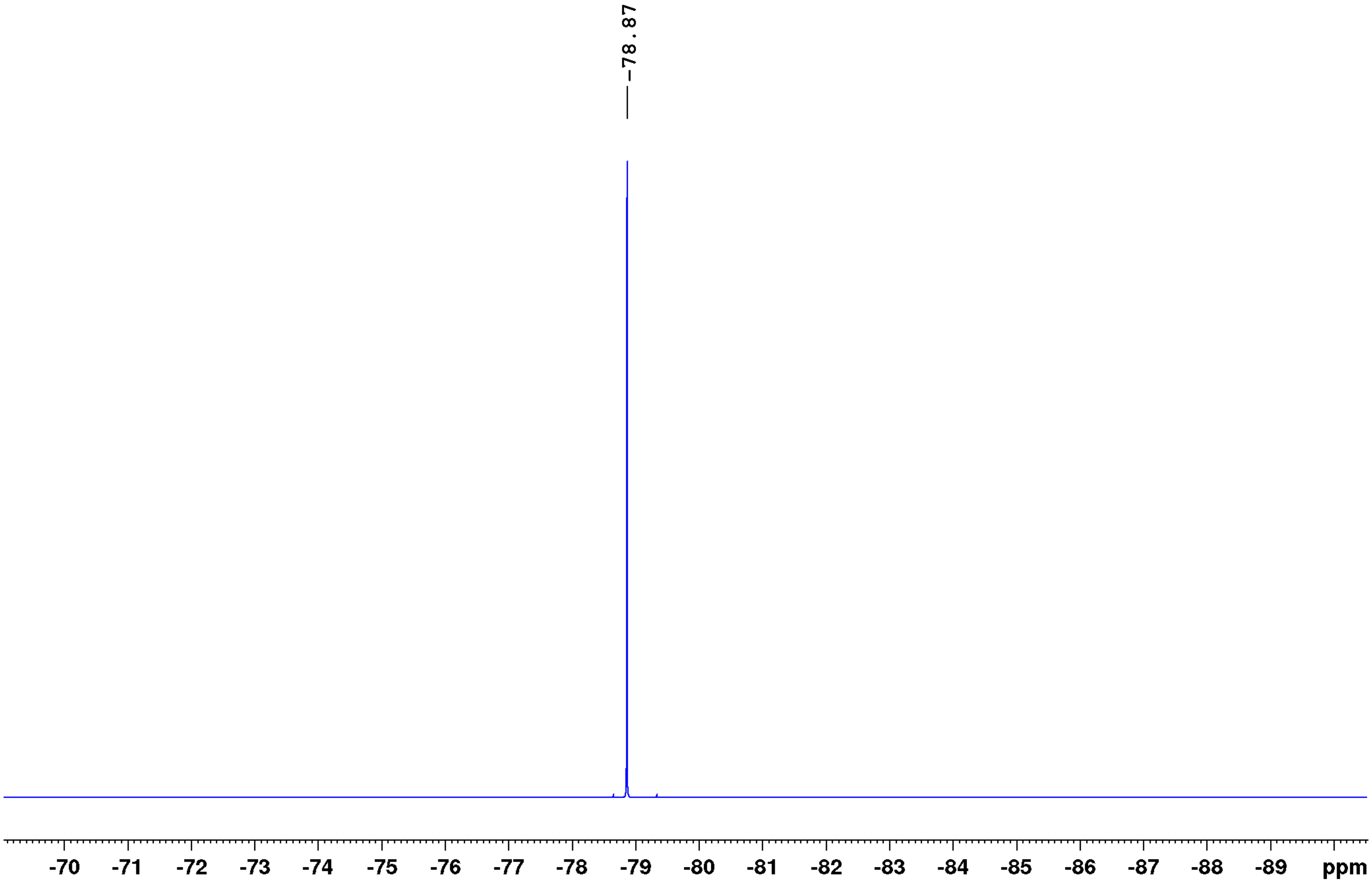

Figure S19. ^{31}P NMR spectrum of **2-PMe$_3$** in CD_2Cl_2.

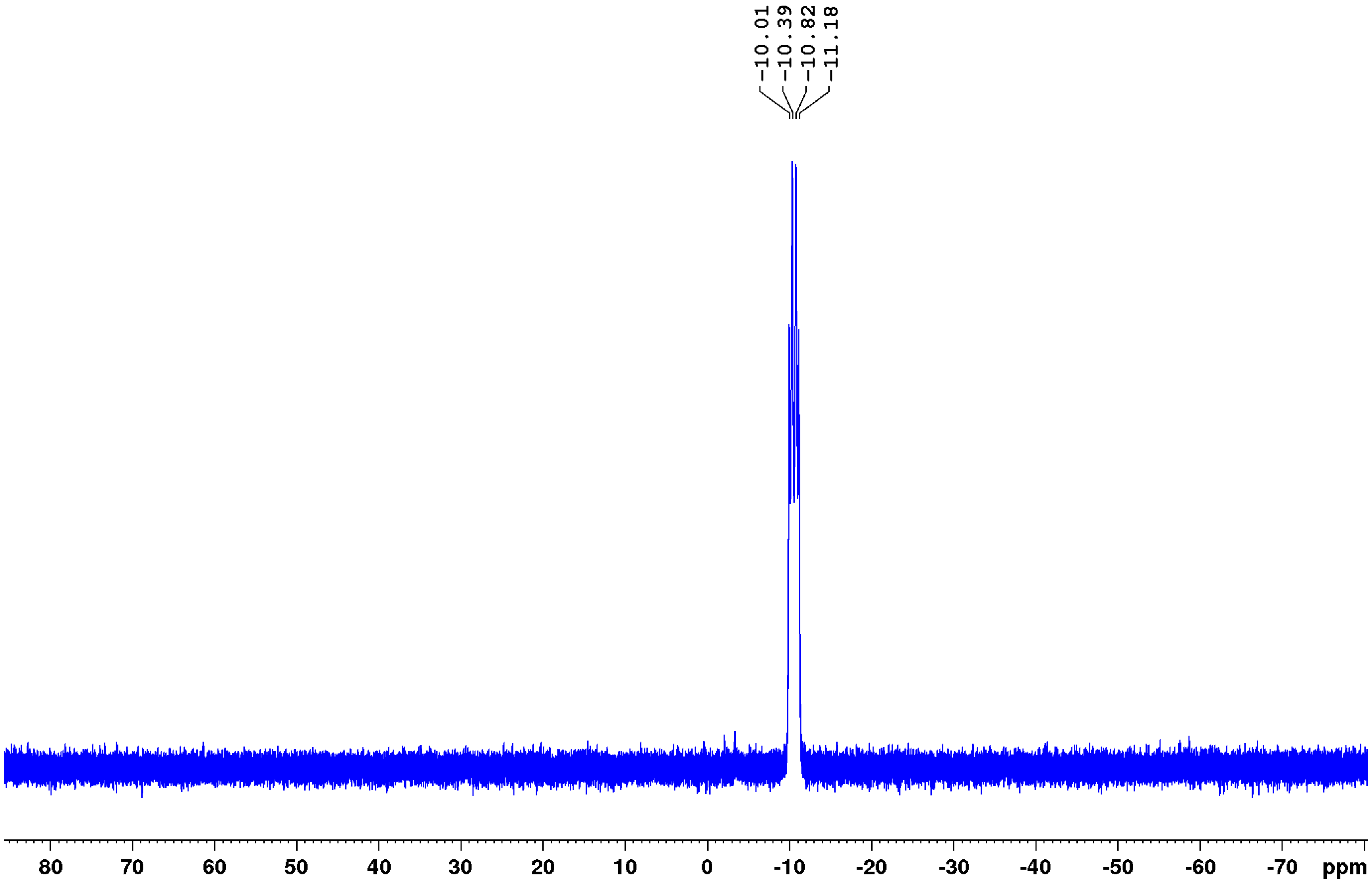

Figure S20. $^{1}H\{^{11}B\}$ NMR spectrum of **2-Pyr** in $CDCl_3$.

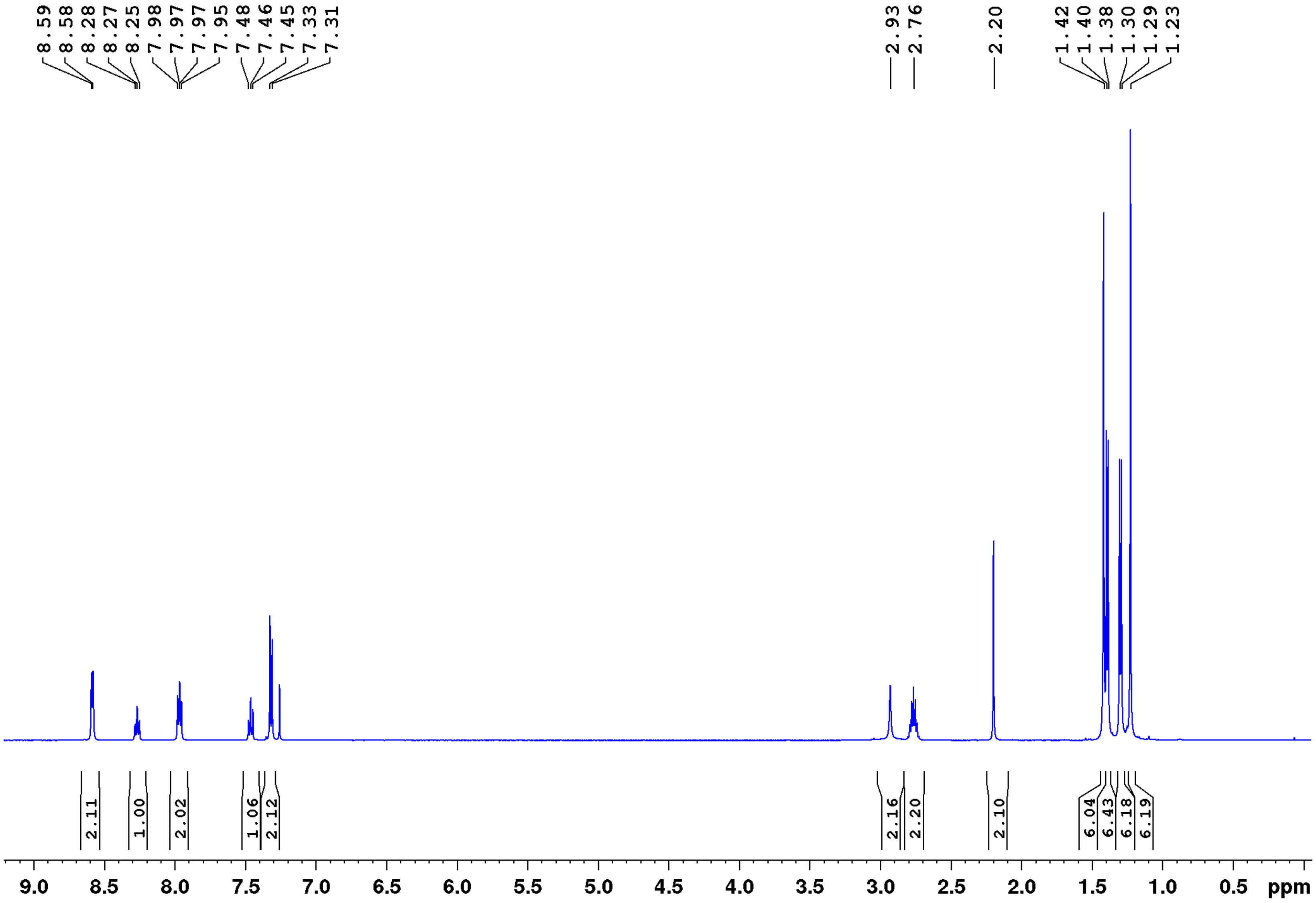

Figure S21. $^{13}C\{^1H\}$ NMR spectrum of **2-Pyr** in $CDCl_3$.

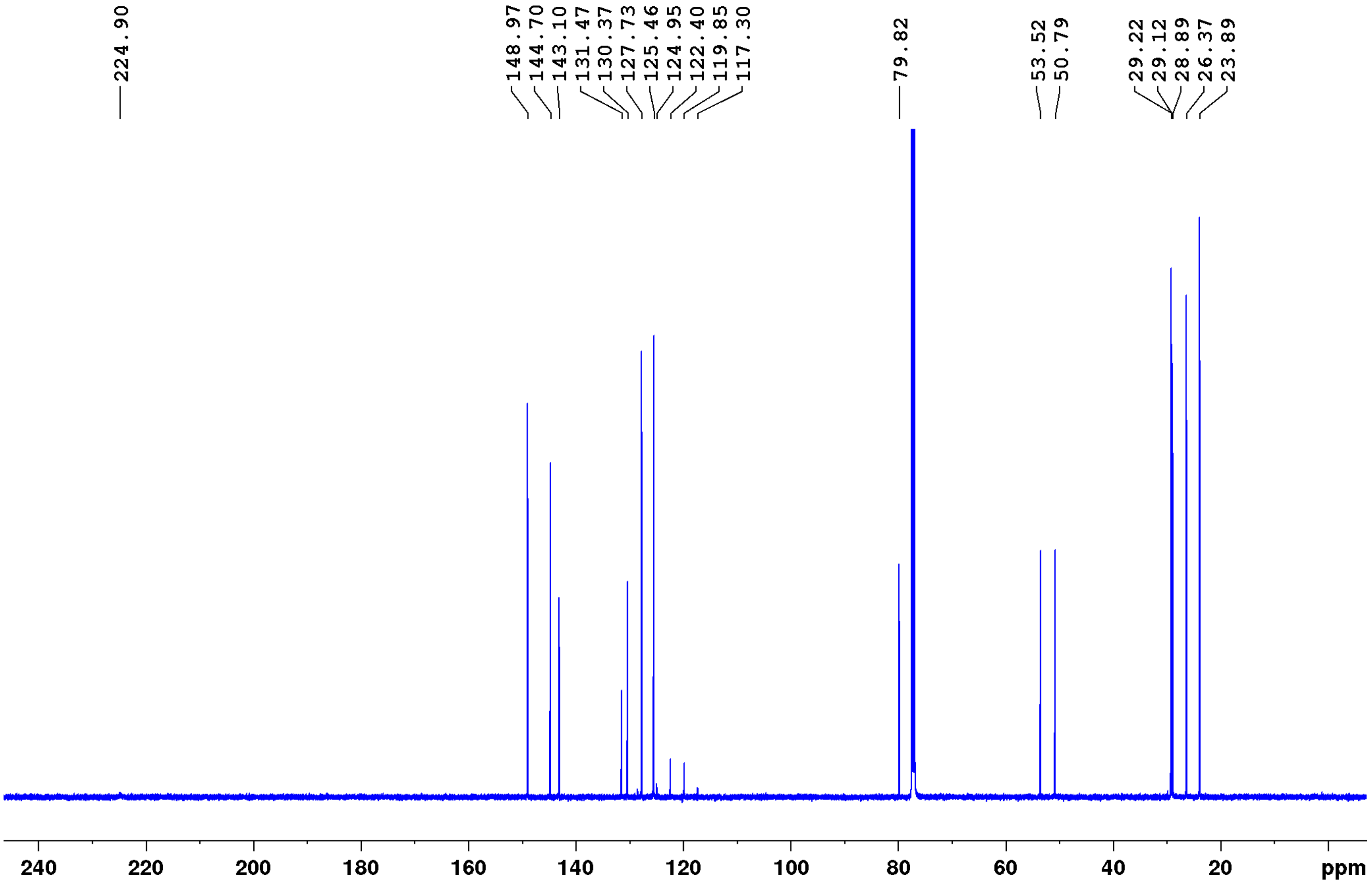

Figure S22. ^{11}B NMR spectrum of **2-Pyr** in $CDCl_3$.

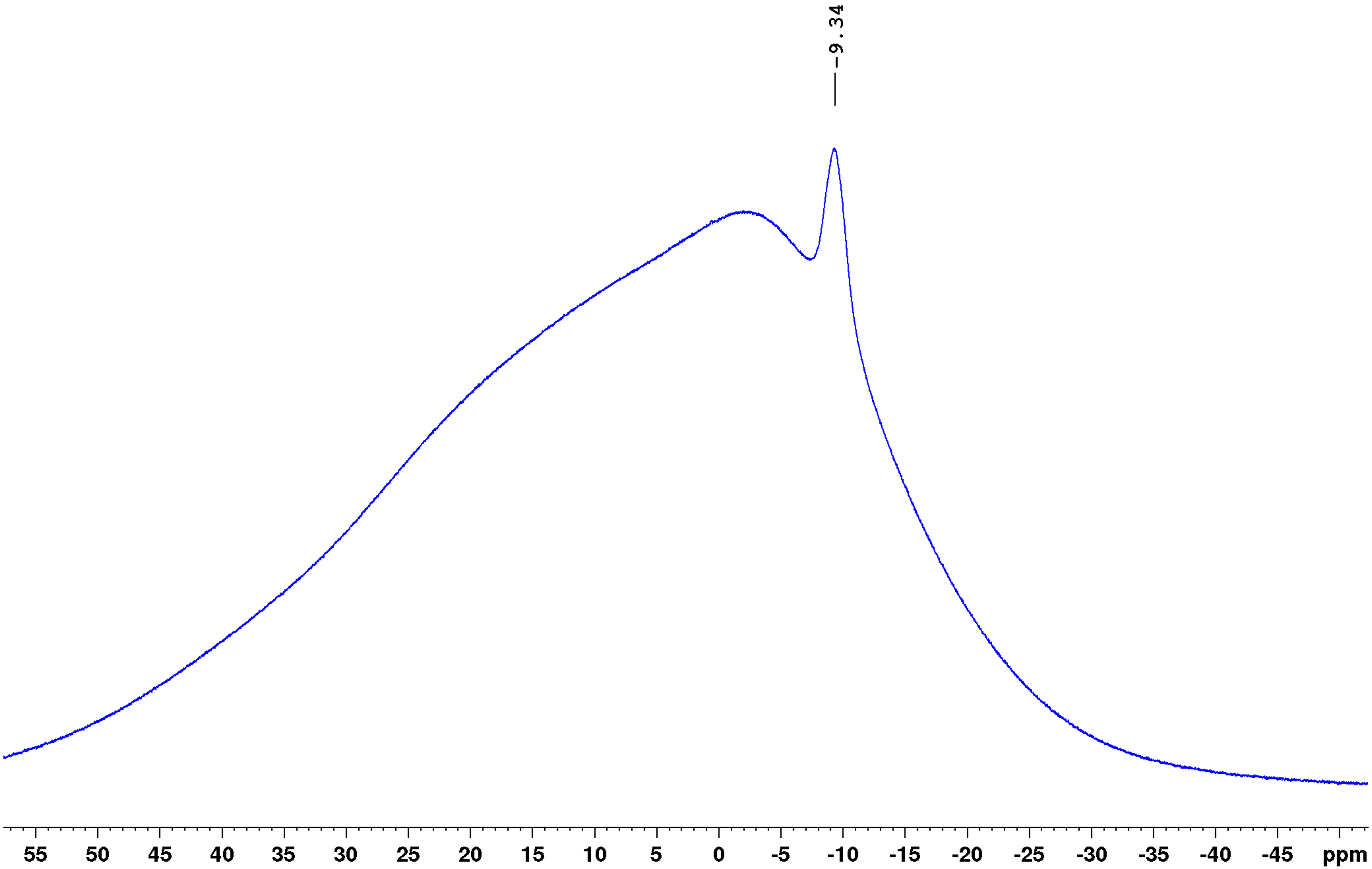

Figure S23. ^{19}F NMR spectrum of **2-Pyr** in $CDCl_3$.

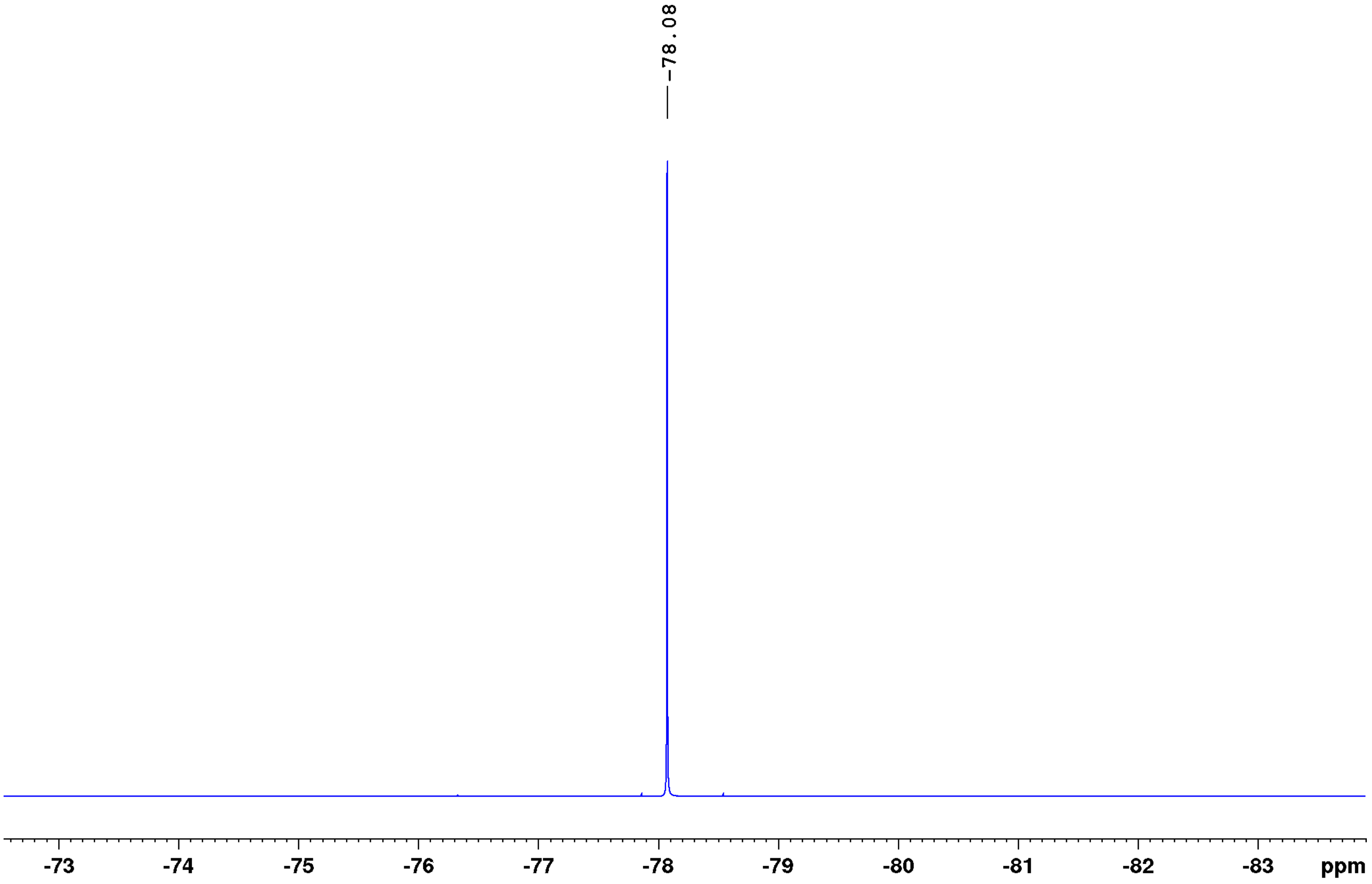

Figure S24. $^1H\{^{11}B\}$ NMR spectrum of **2-DMAP** in $CDCl_3$.

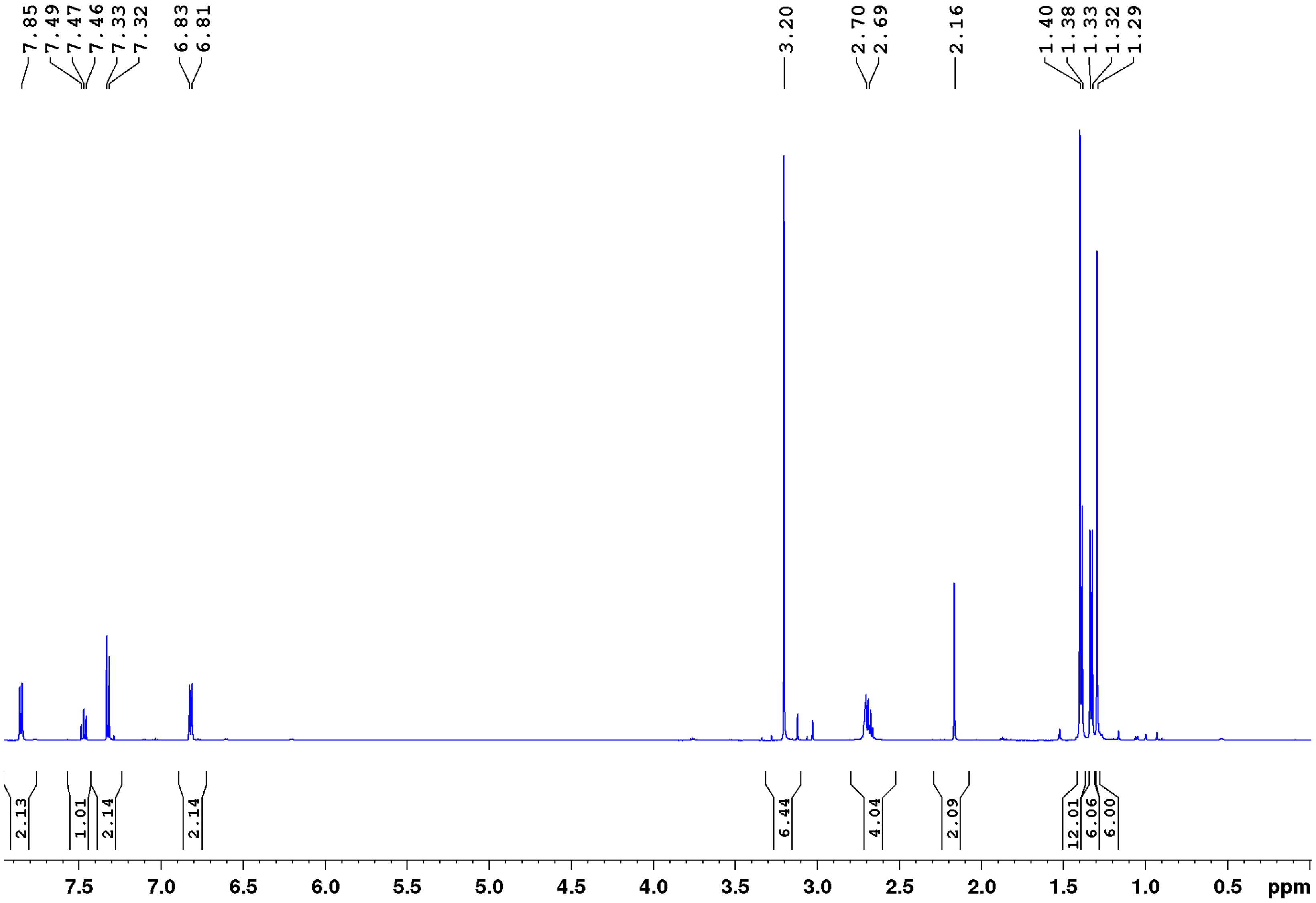

Figure S25. $^{13}C\{^1H\}$ NMR spectrum of **2-DMAP** in $CDCl_3$.

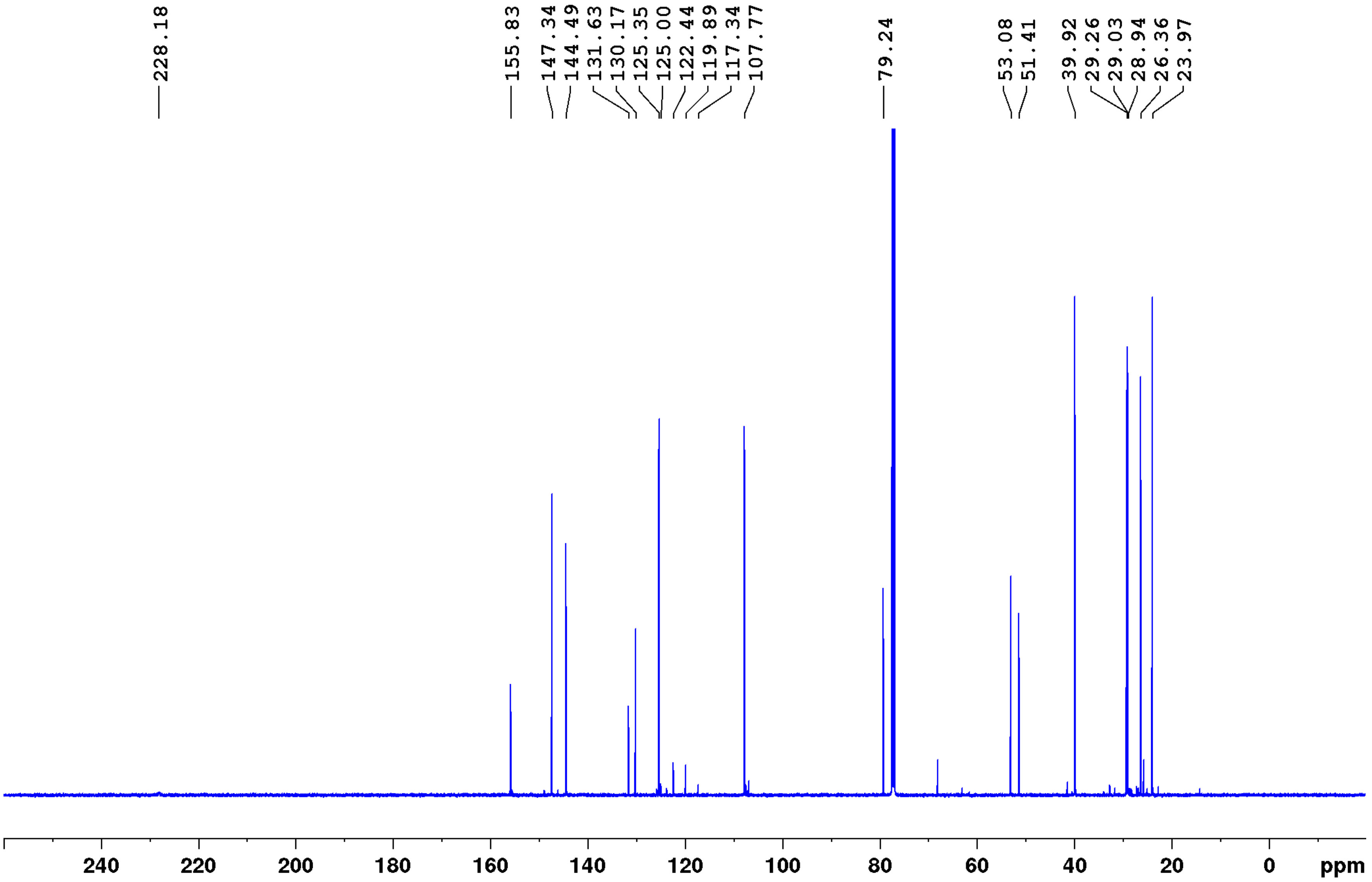

Figure S26. ^{11}B NMR spectrum of **2-DMAP** in $CDCl_3$.

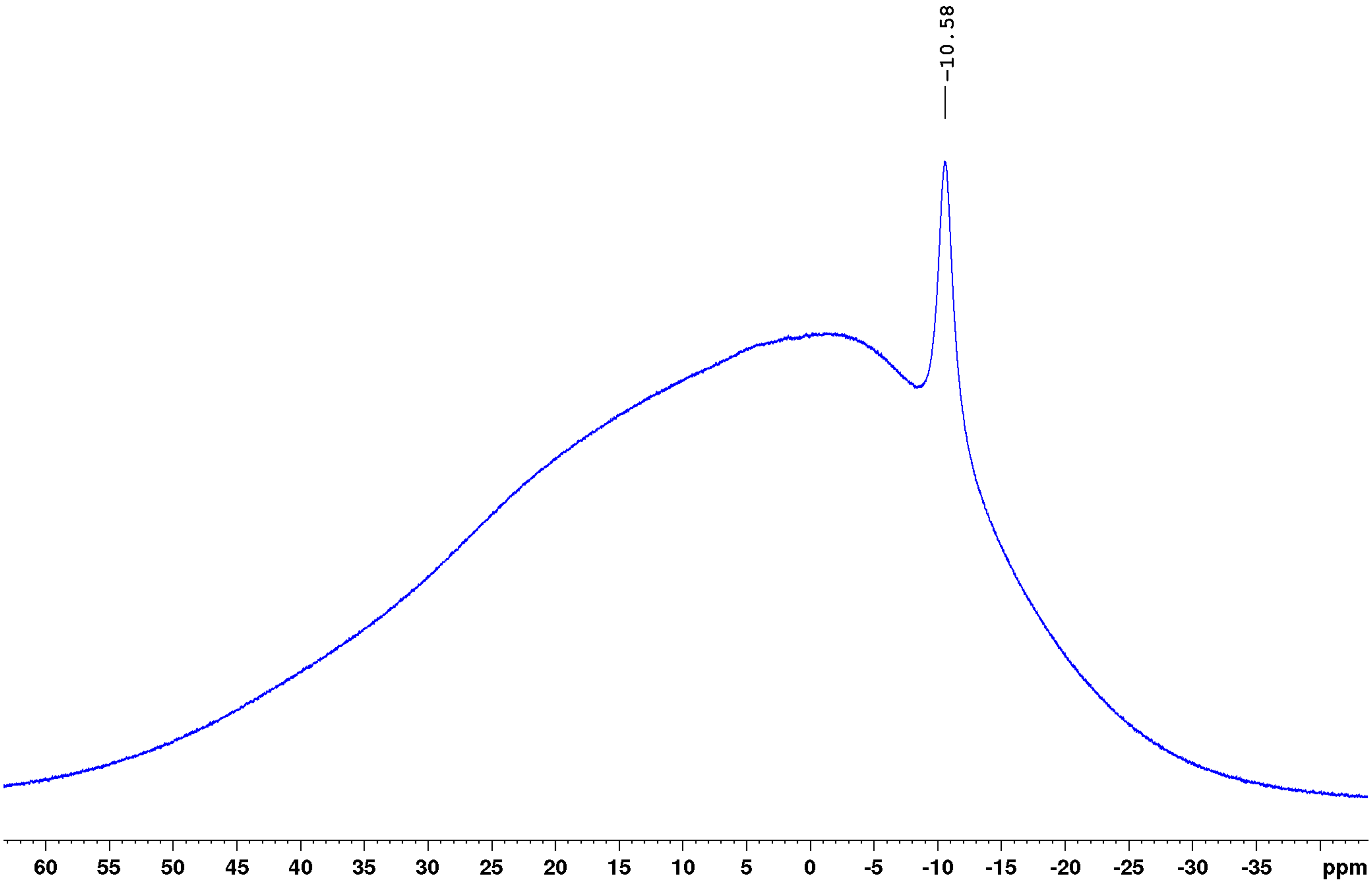

Figure S27. ^{19}F NMR spectrum of **2-DMAP** in $CDCl_3$.

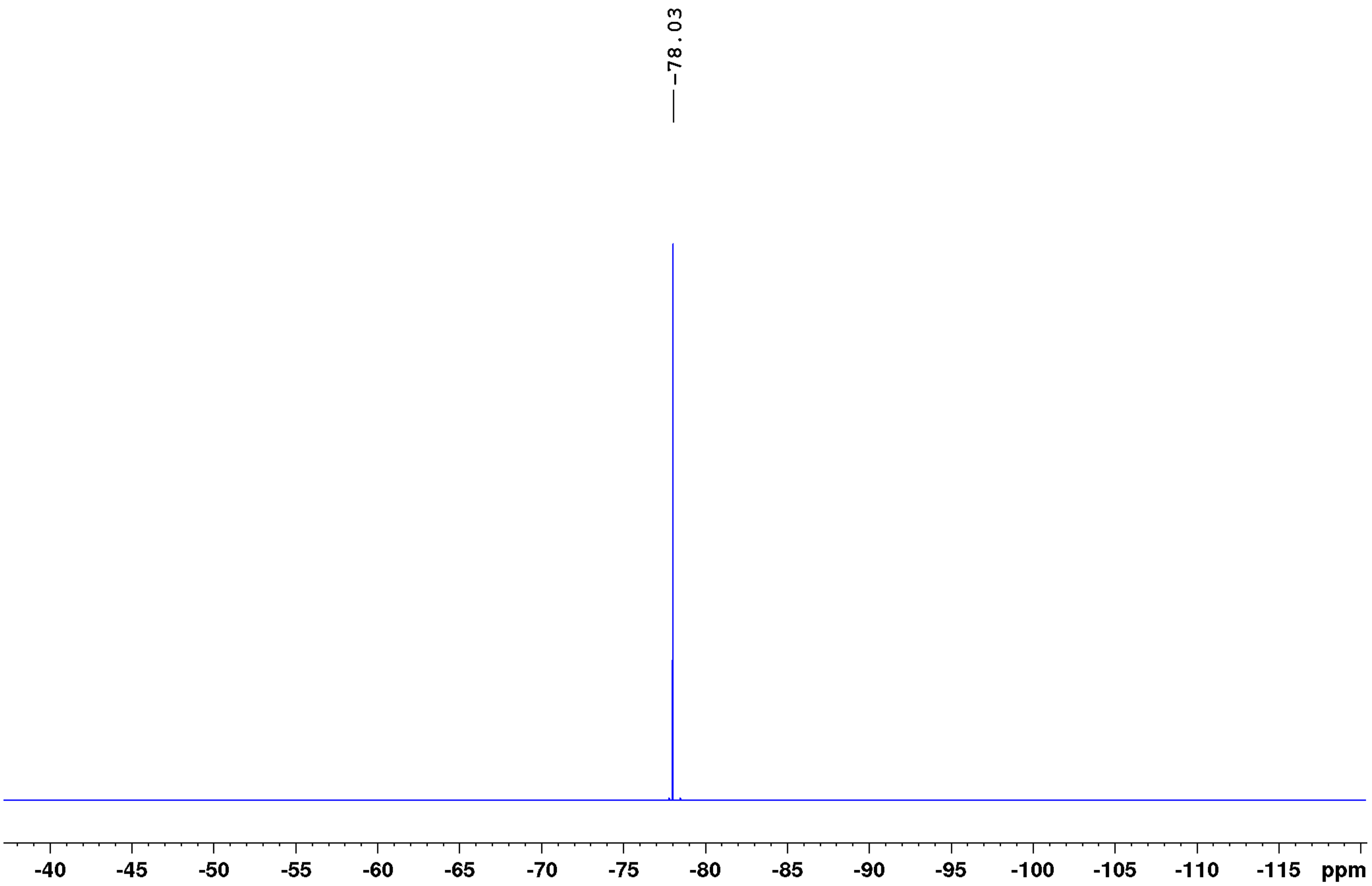

Figure S28. ^{1}H NMR spectrum of **3** in CD_2Cl_2.

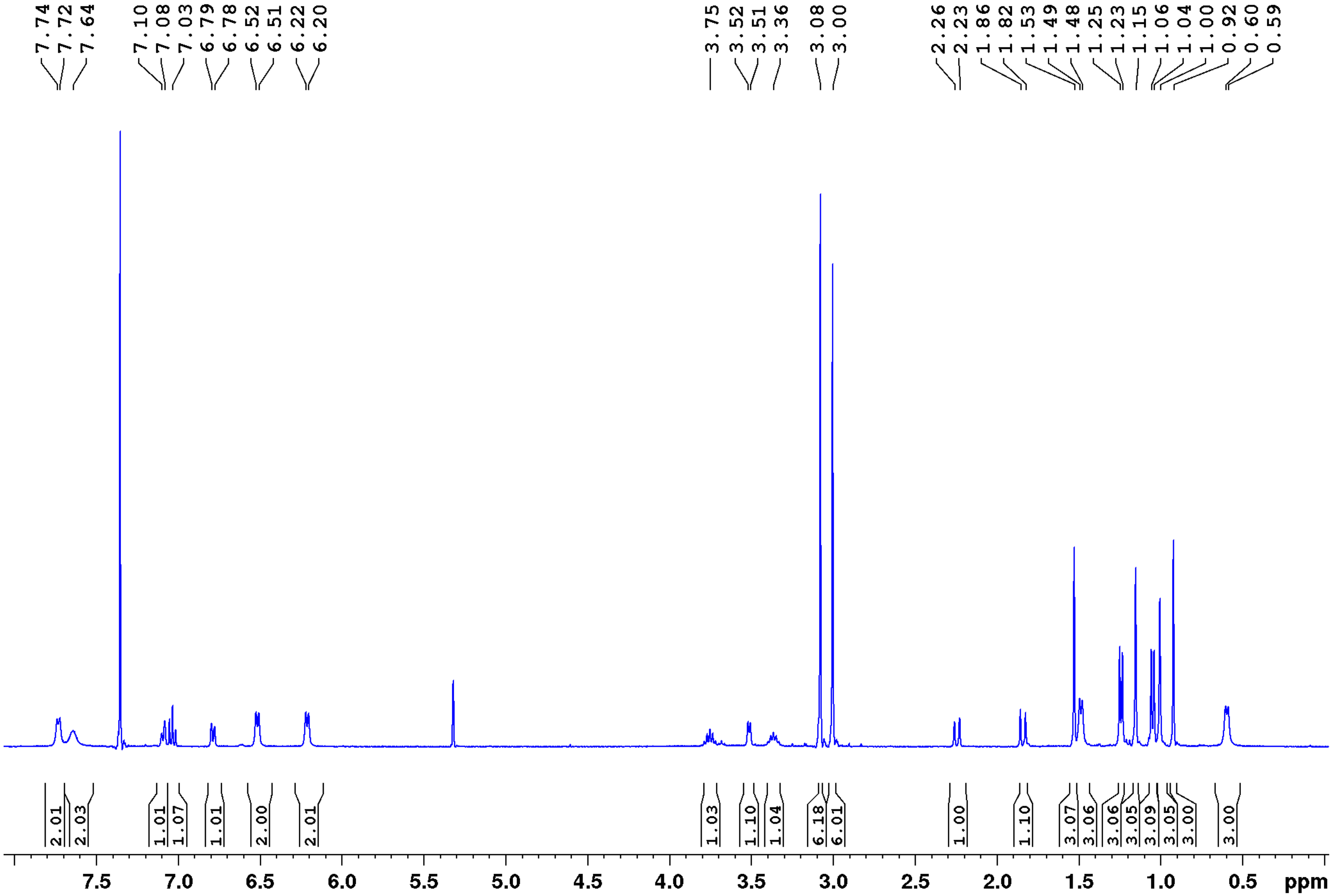

Figure S29. $^{13}C\{^{1}H\}$ NMR spectrum of **3** in CD_2Cl_2.

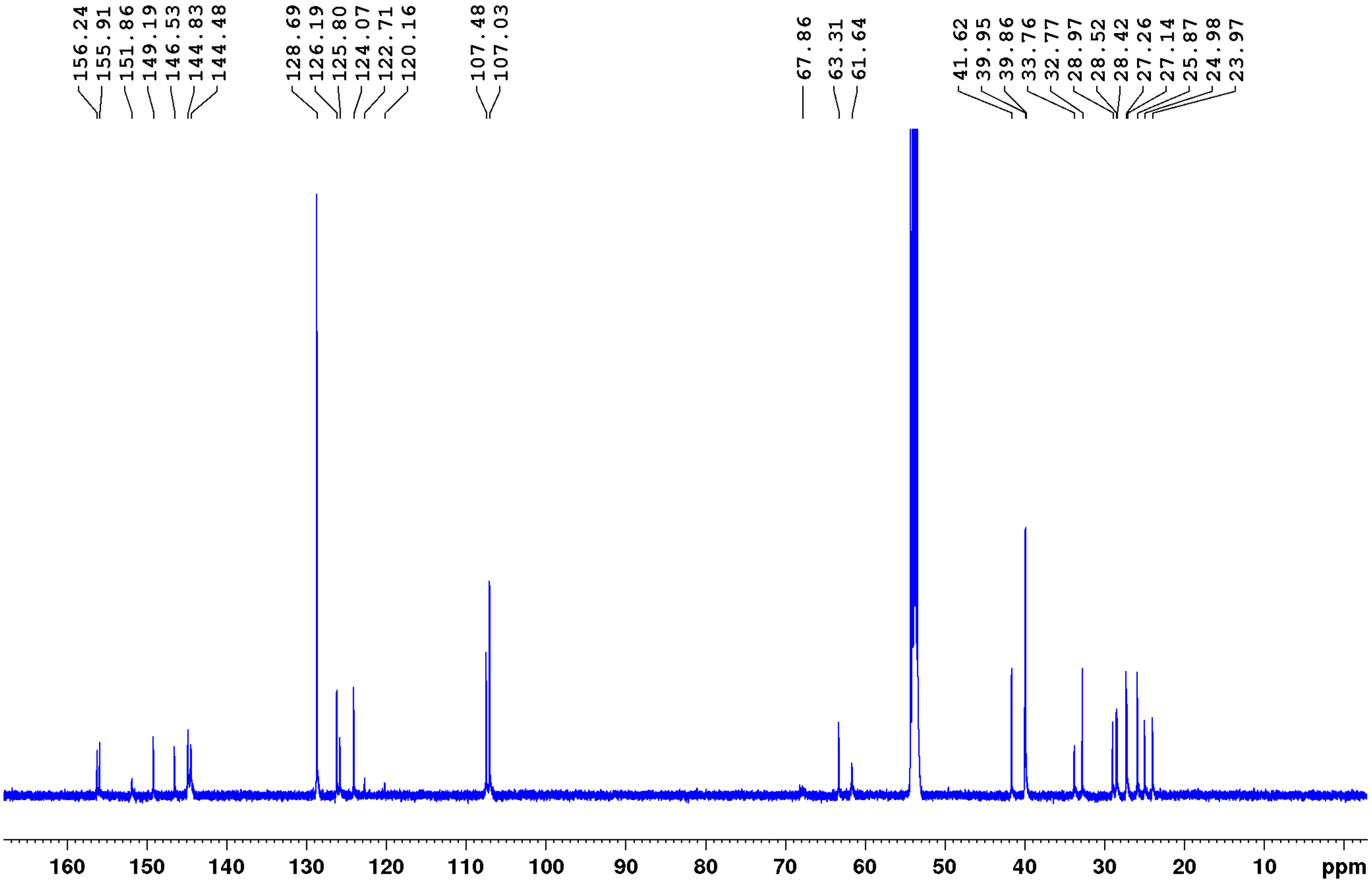

Figure S30. ^{11}B NMR spectrum of **3** in CD_2Cl_2.

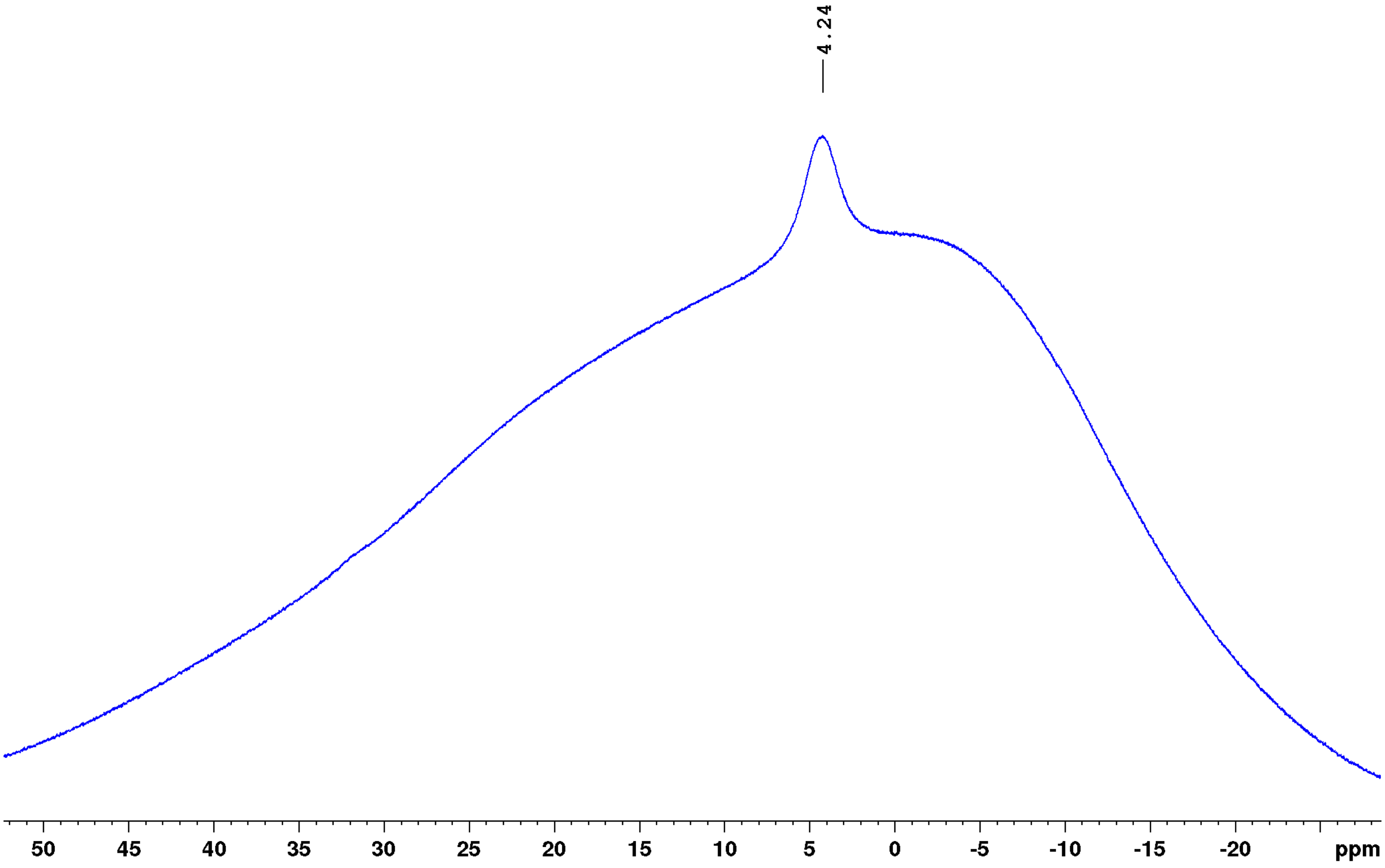

Figure S31. ^{19}F NMR spectrum of **3** in CD_2Cl_2.

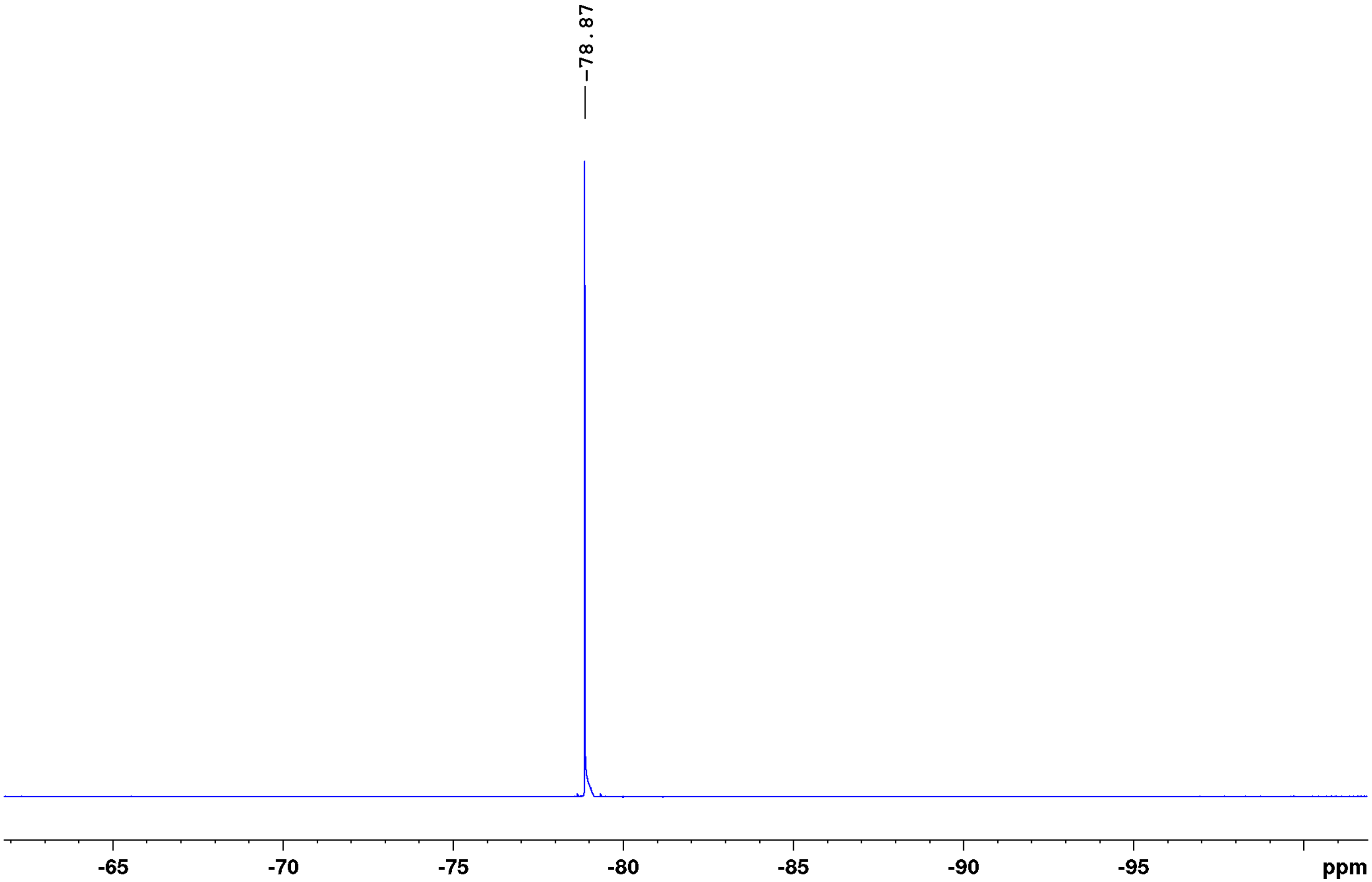

Figure S32. $^{1}H\{^{11}B\}$ NMR spectrum of **4-Bipy** in $CDCl_3$.

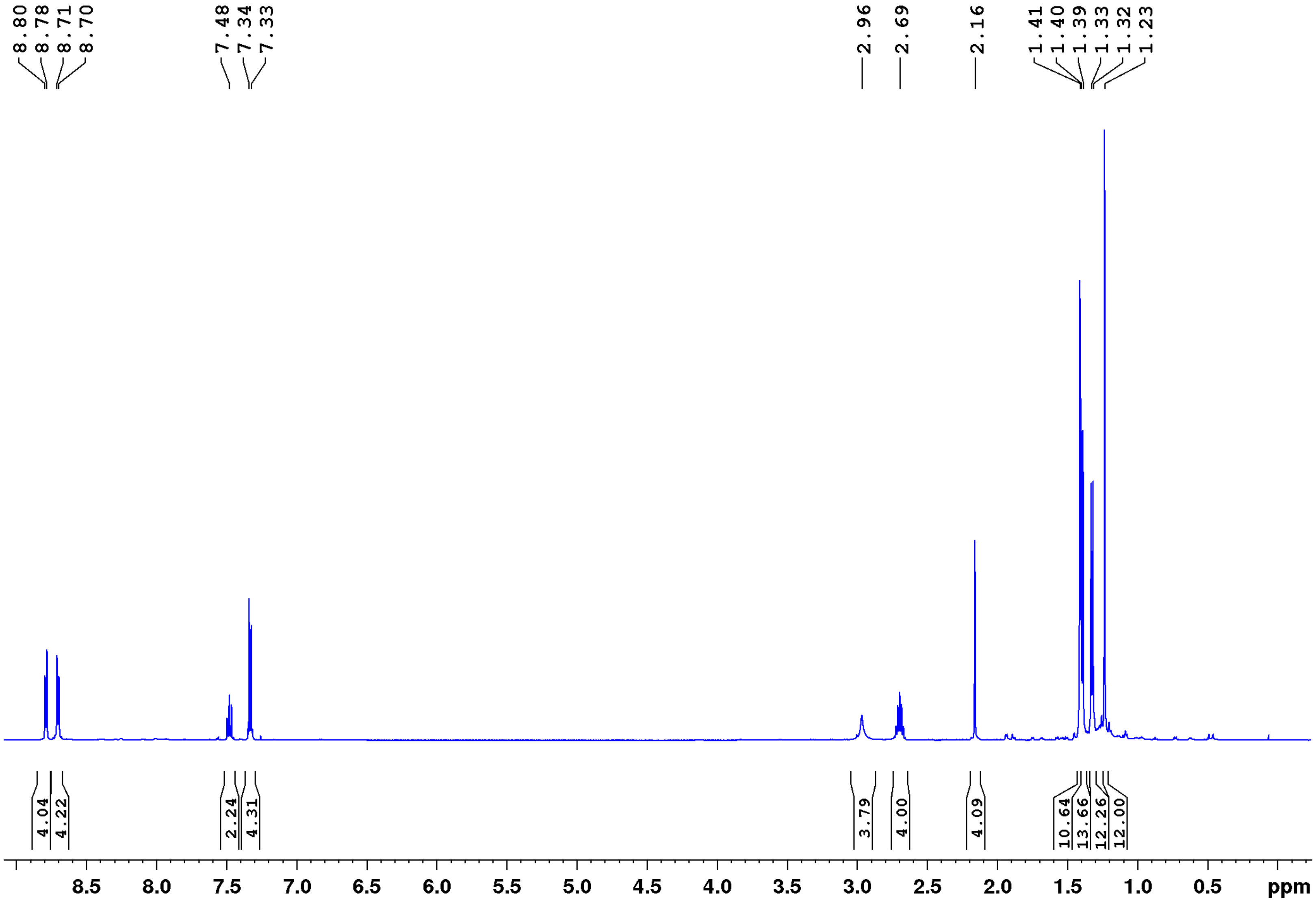

Figure S33. $^{13}C\{^{1}H\}$ NMR spectrum of **4-Bipy** in $CDCl_3$.

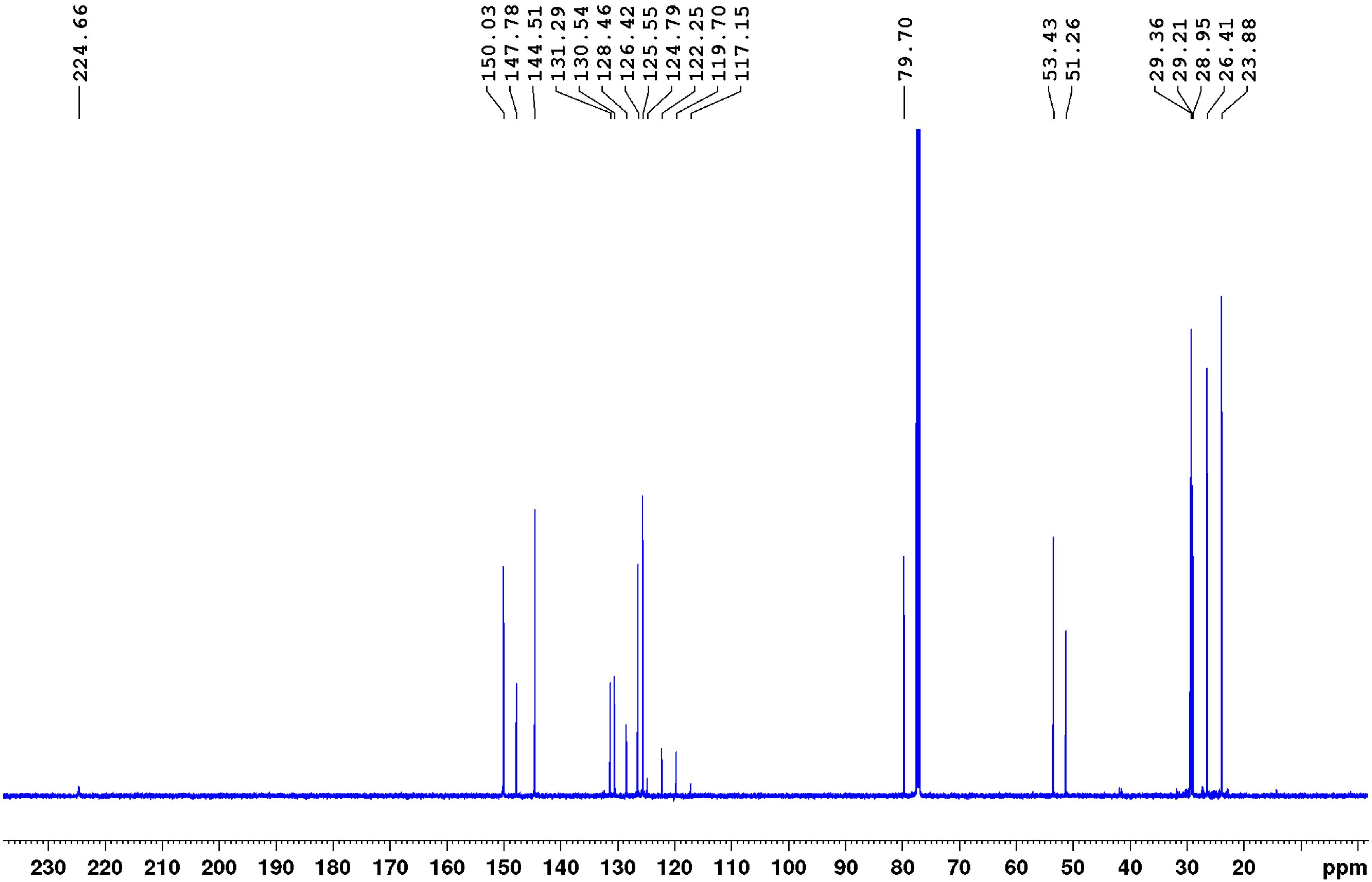

Figure S34. ^{11}B NMR spectrum of **4-Bipy** in $CDCl_3$.

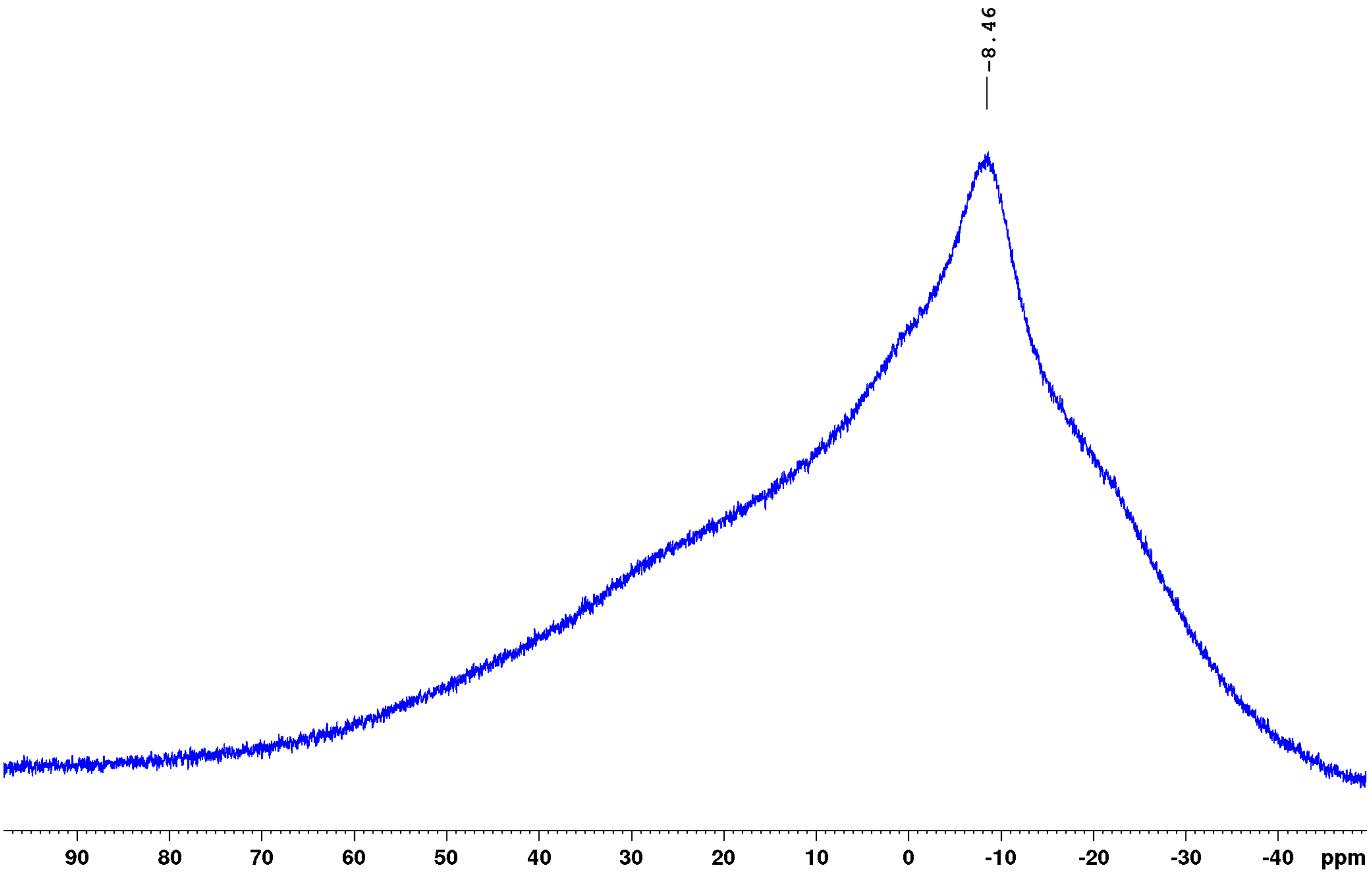

Figure S35. ^{19}F NMR spectrum of **4-Bipy** in $CDCl_3$.

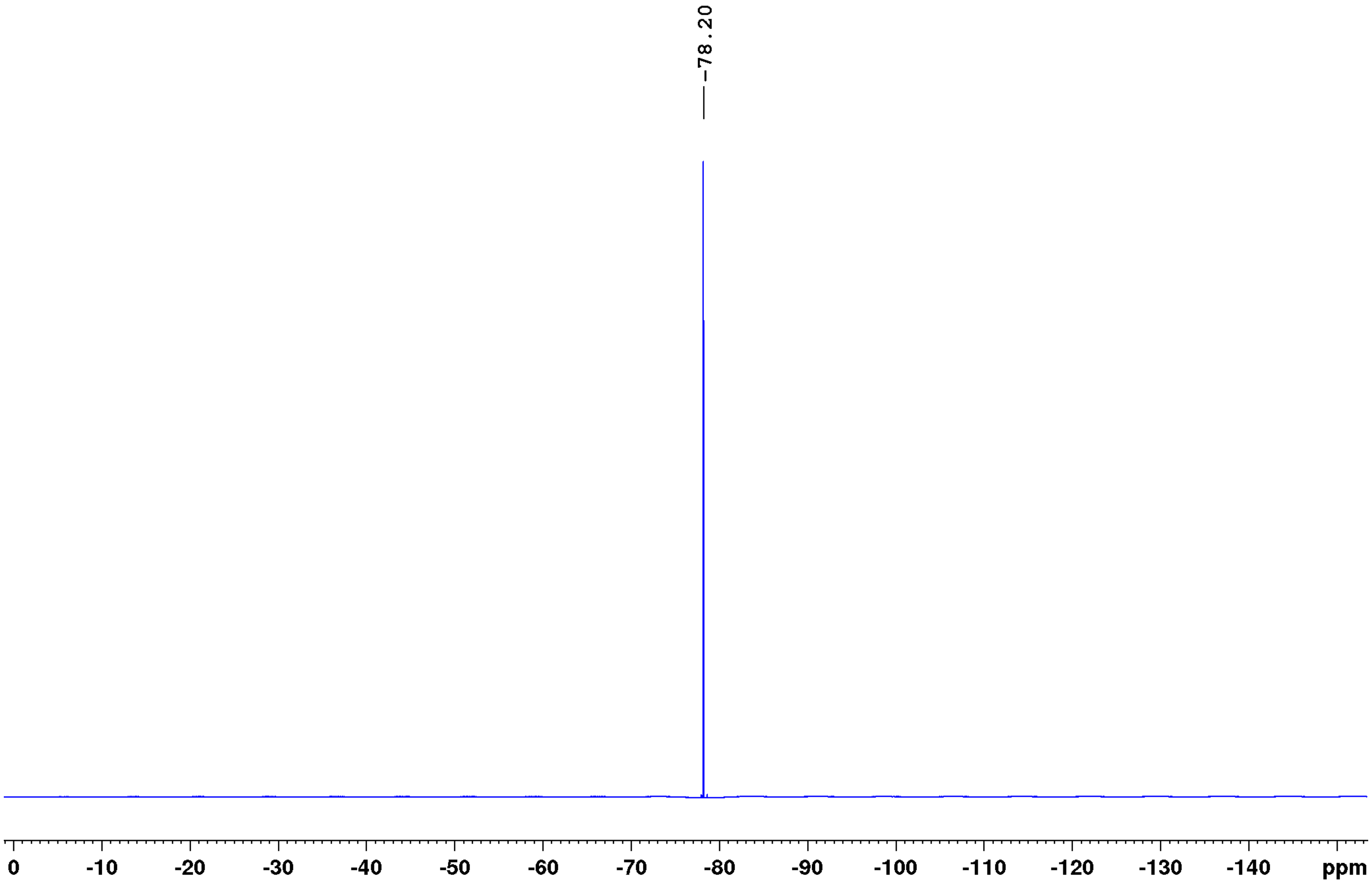

Figure S36. $^{1}H\{^{11}B\}$ NMR spectrum of **5** in C_6D_6.

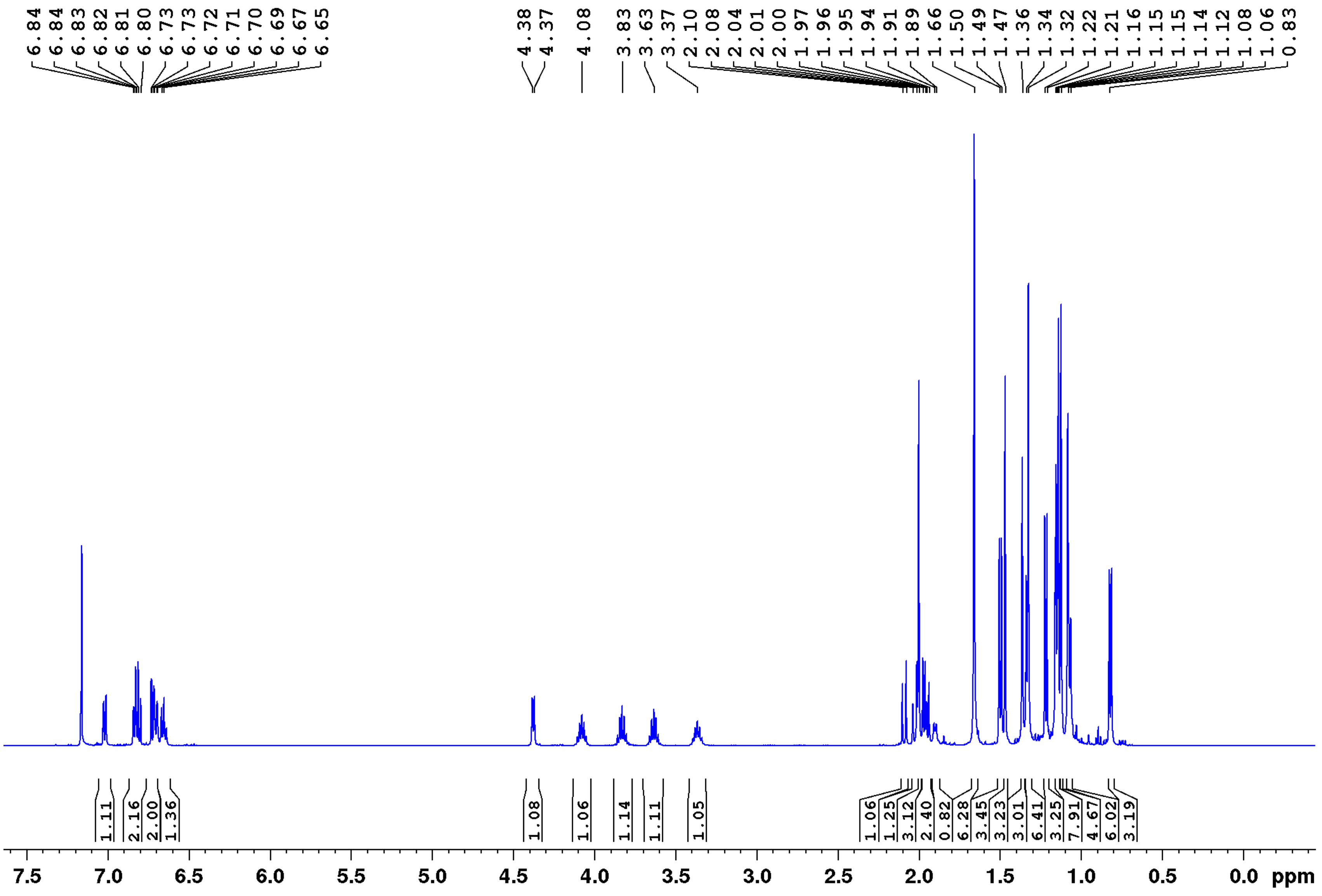

Figure S37. $^{13}C\{^{1}H\}$ NMR spectrum of **5** in C_6D_6.

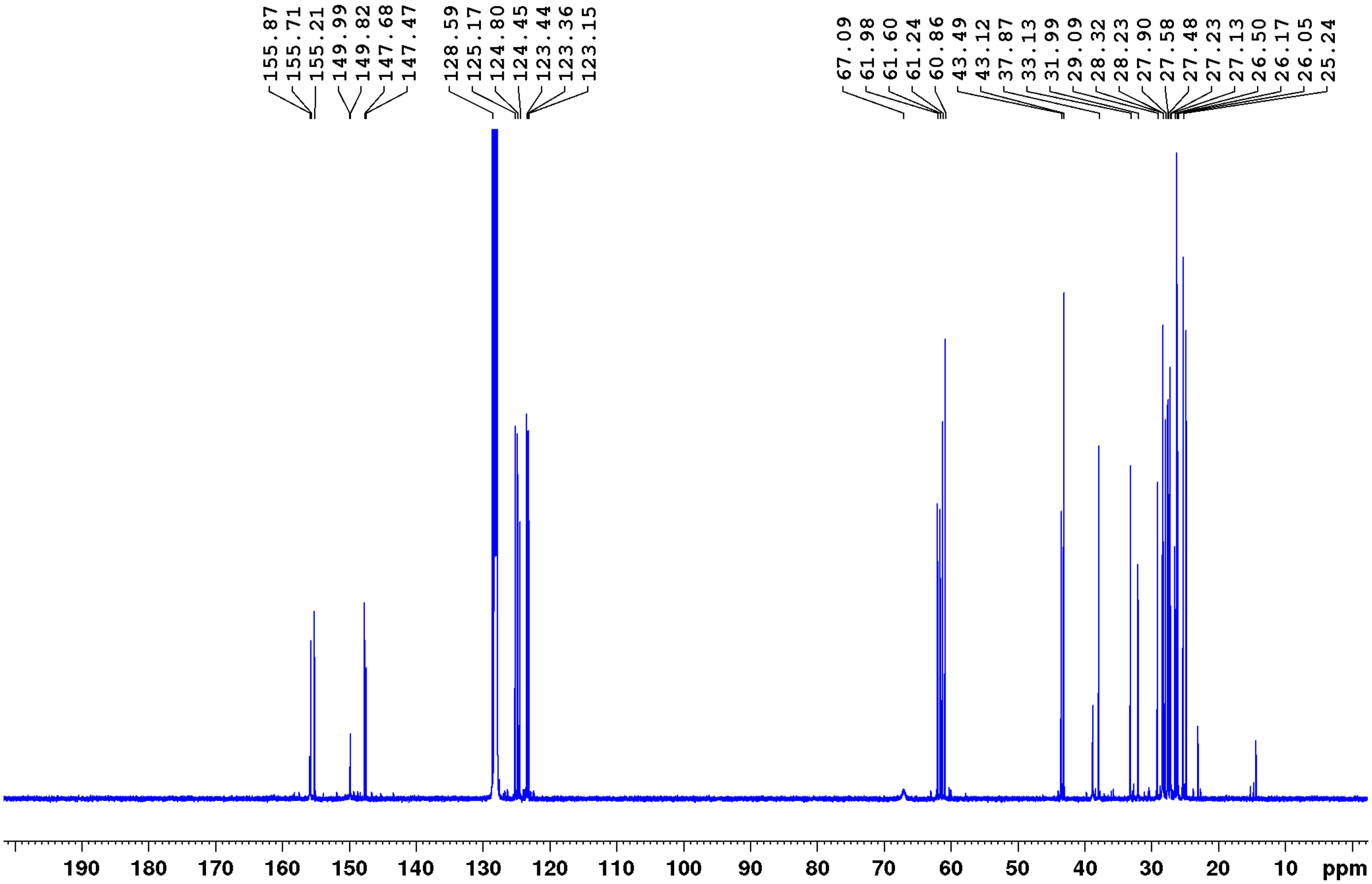

Figure S38. ^{11}B NMR spectrum of **5** in C_6D_6.

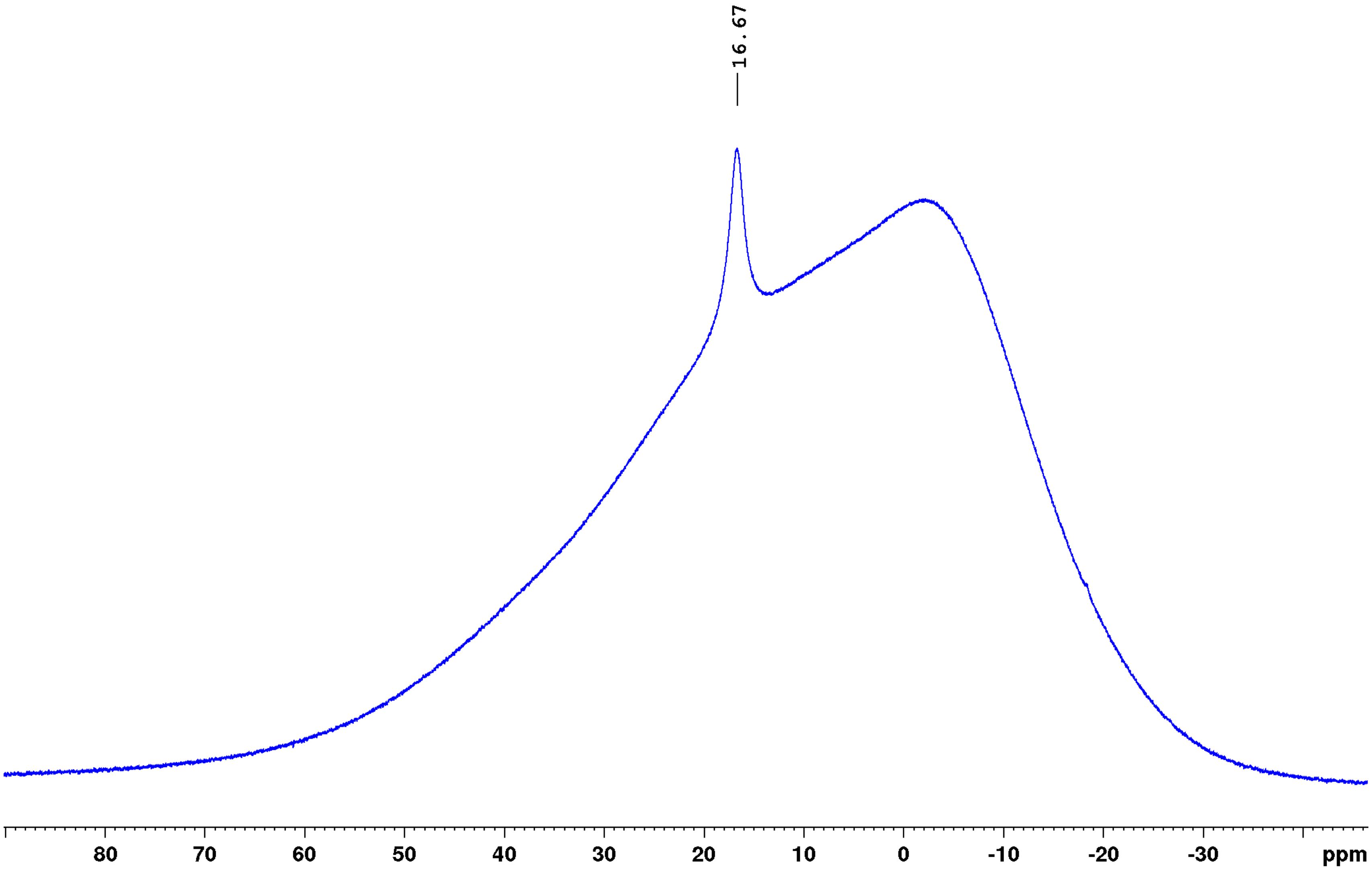

Figure S39. ^{1}H NMR spectrum of **5-thf** in C_6D_6.

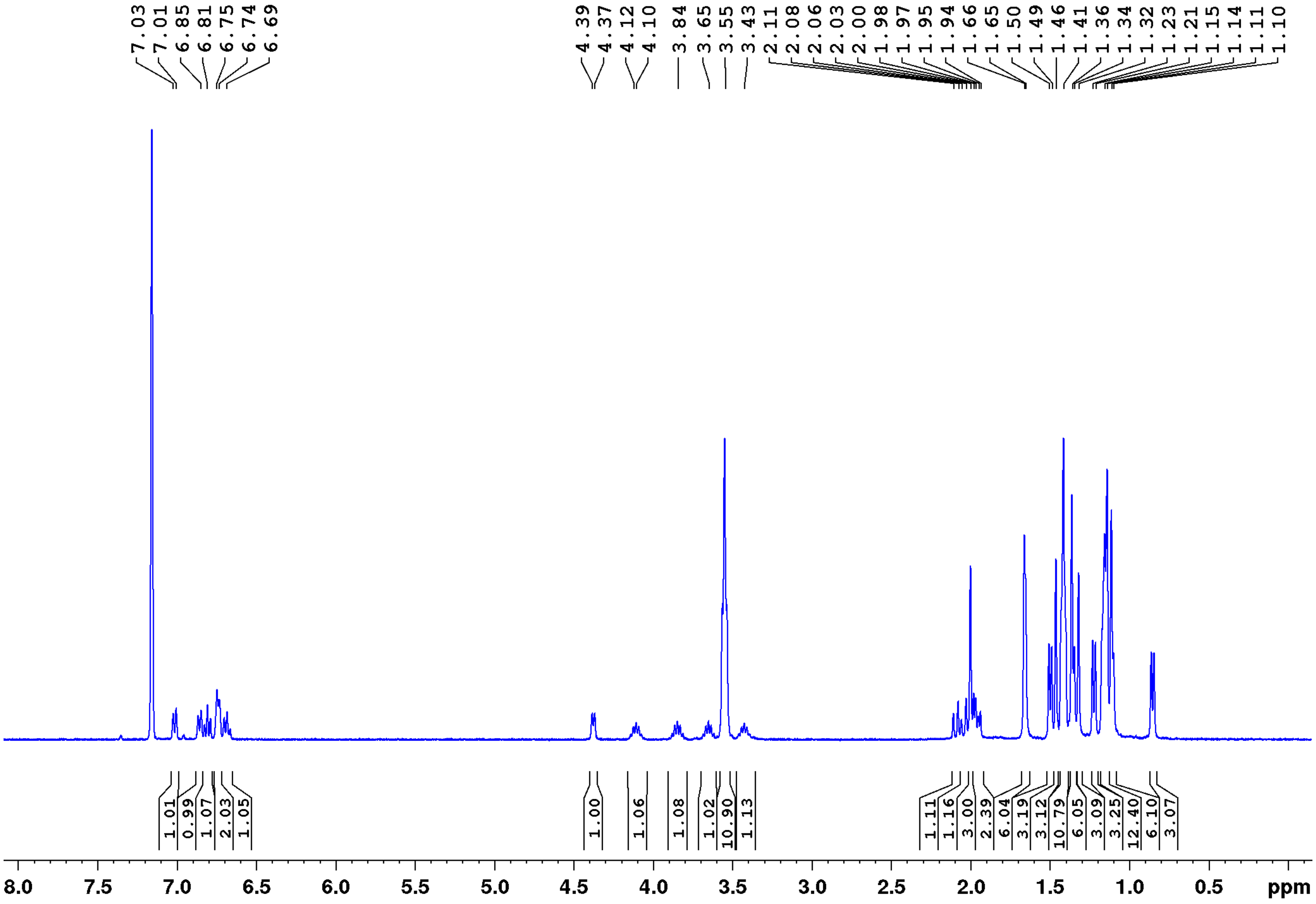

Figure S40. ^{11}B NMR spectrum of **5-thf** in C_6D_6.

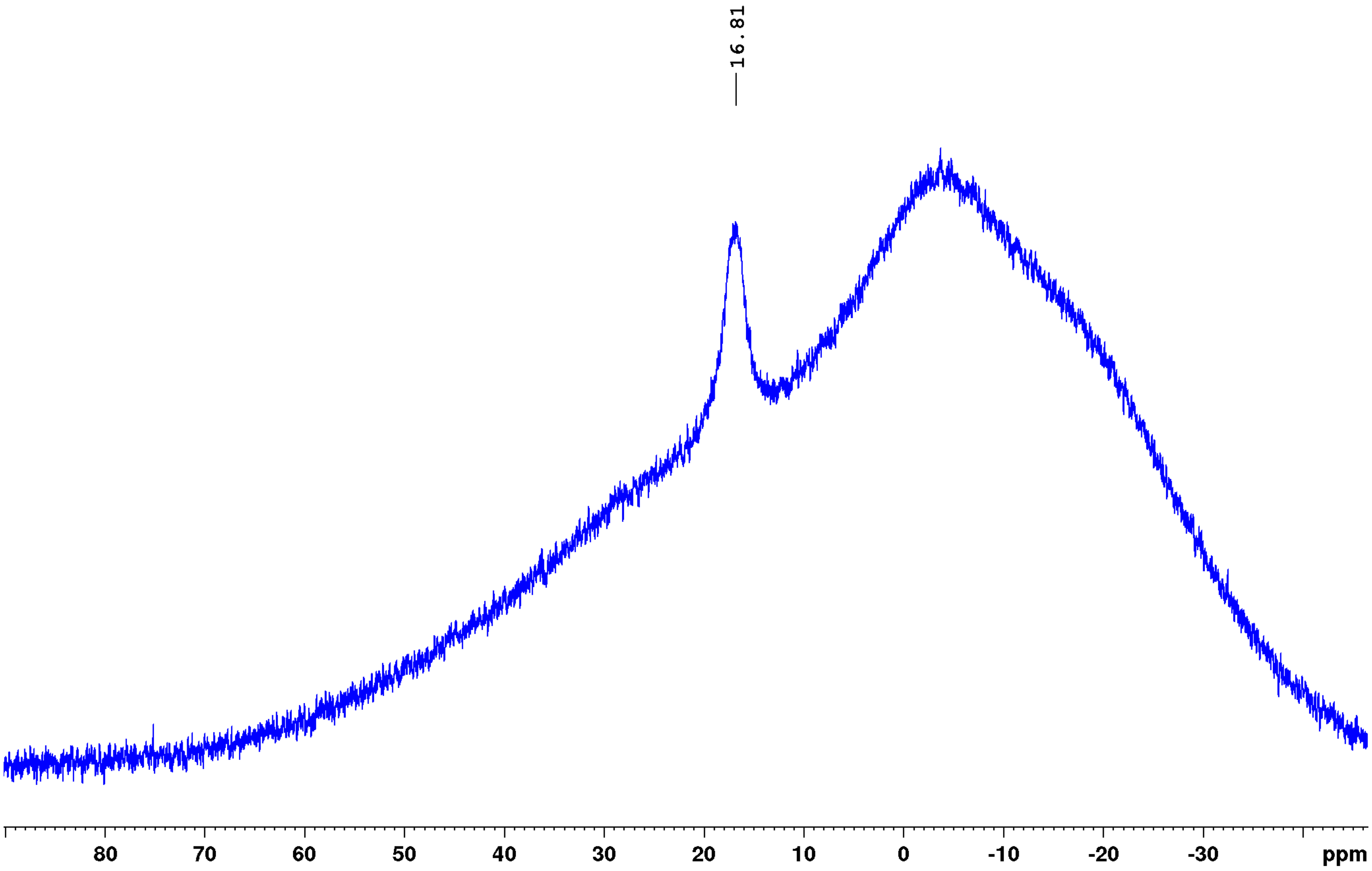

Figure S41. ^{1}H NMR spectrum of **7** in C_6D_6.

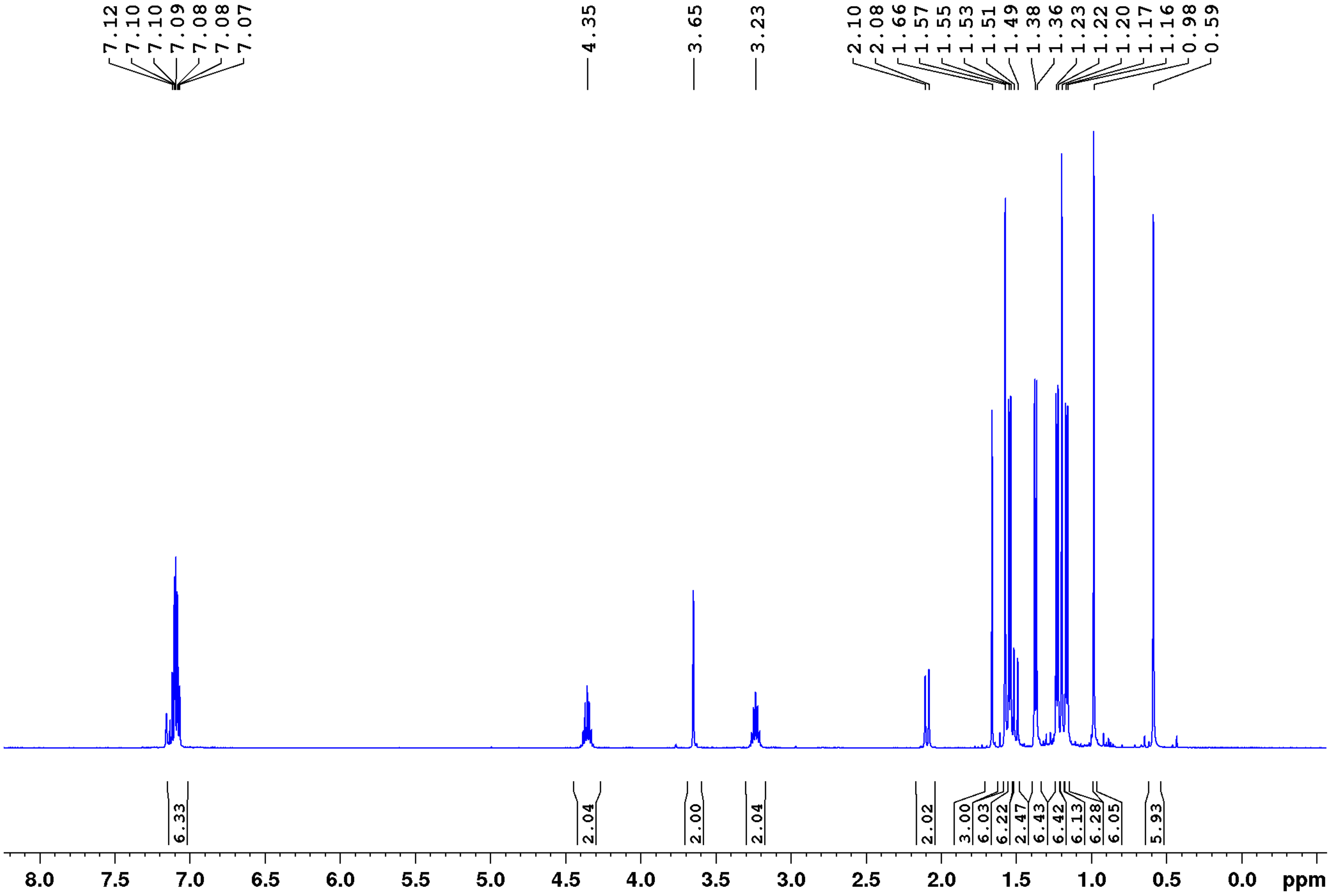

Figure S42. $^{13}C\{^{1}H\}$ NMR spectrum of **7** in C_6D_6.

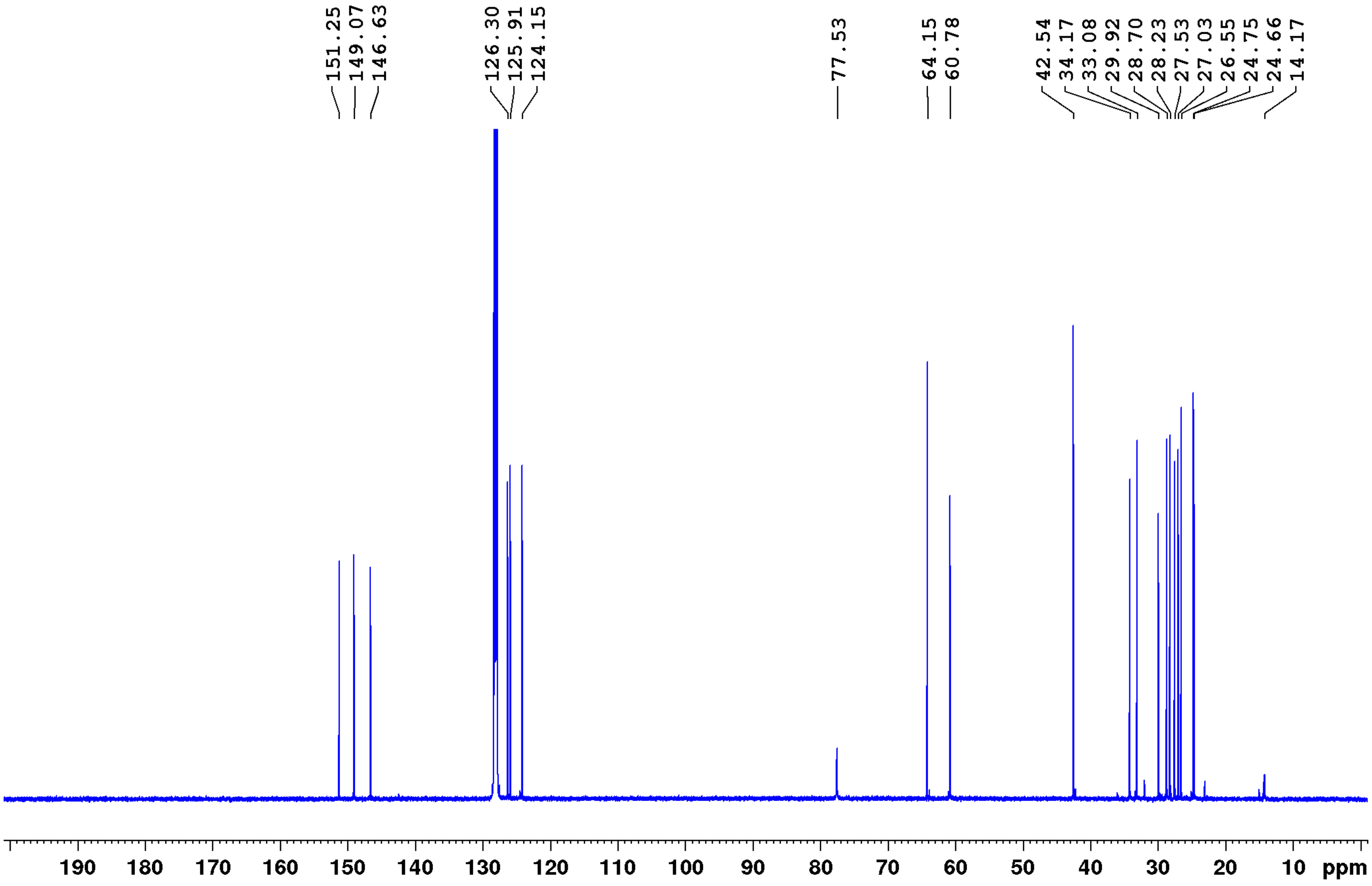

Figure S43. ^{11}B NMR spectrum of **7** in C_6D_6.

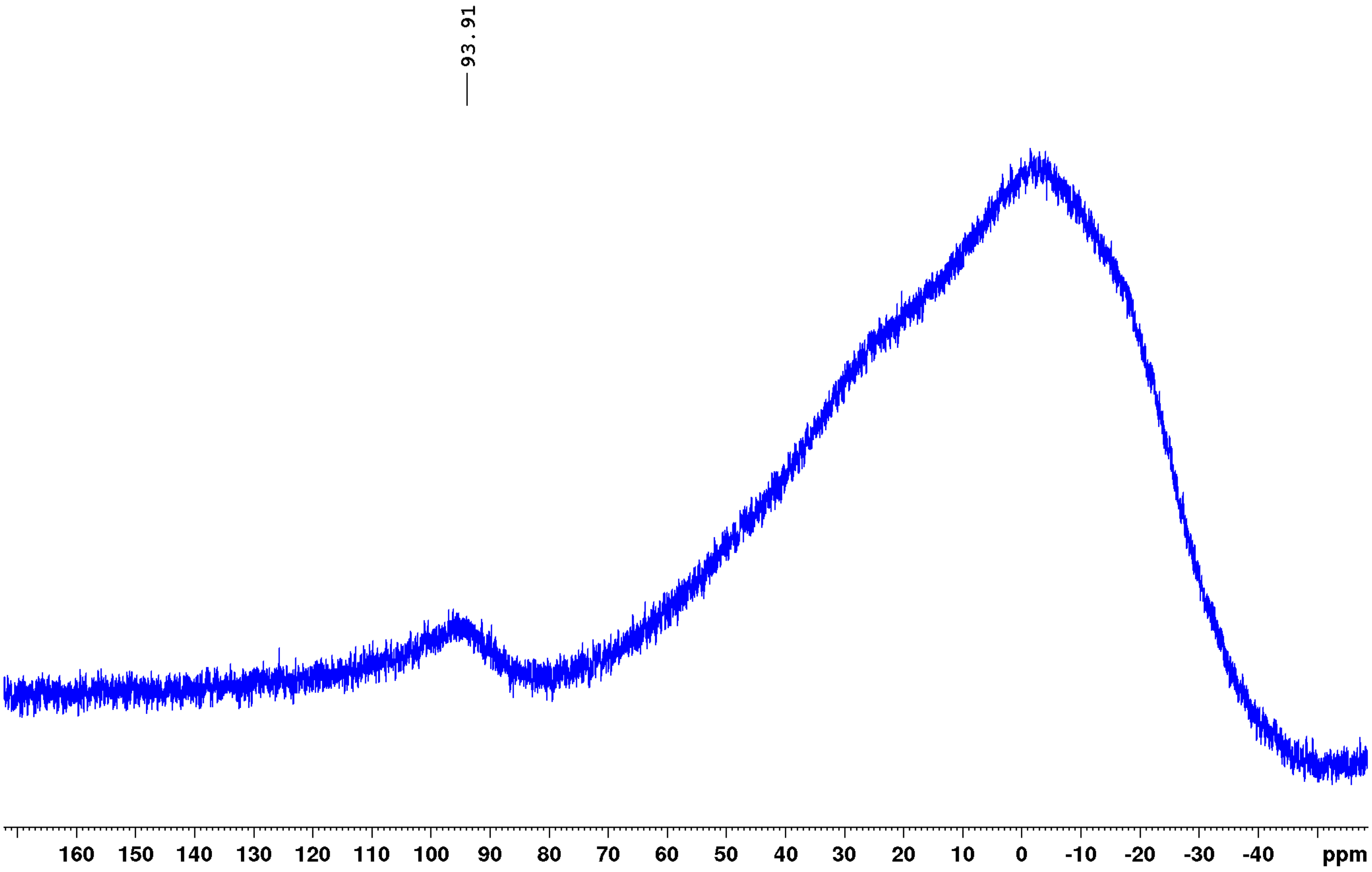

Figure S44. $^{1}H\{^{11}B\}$ NMR spectrum of **8** in $CDCl_3$.

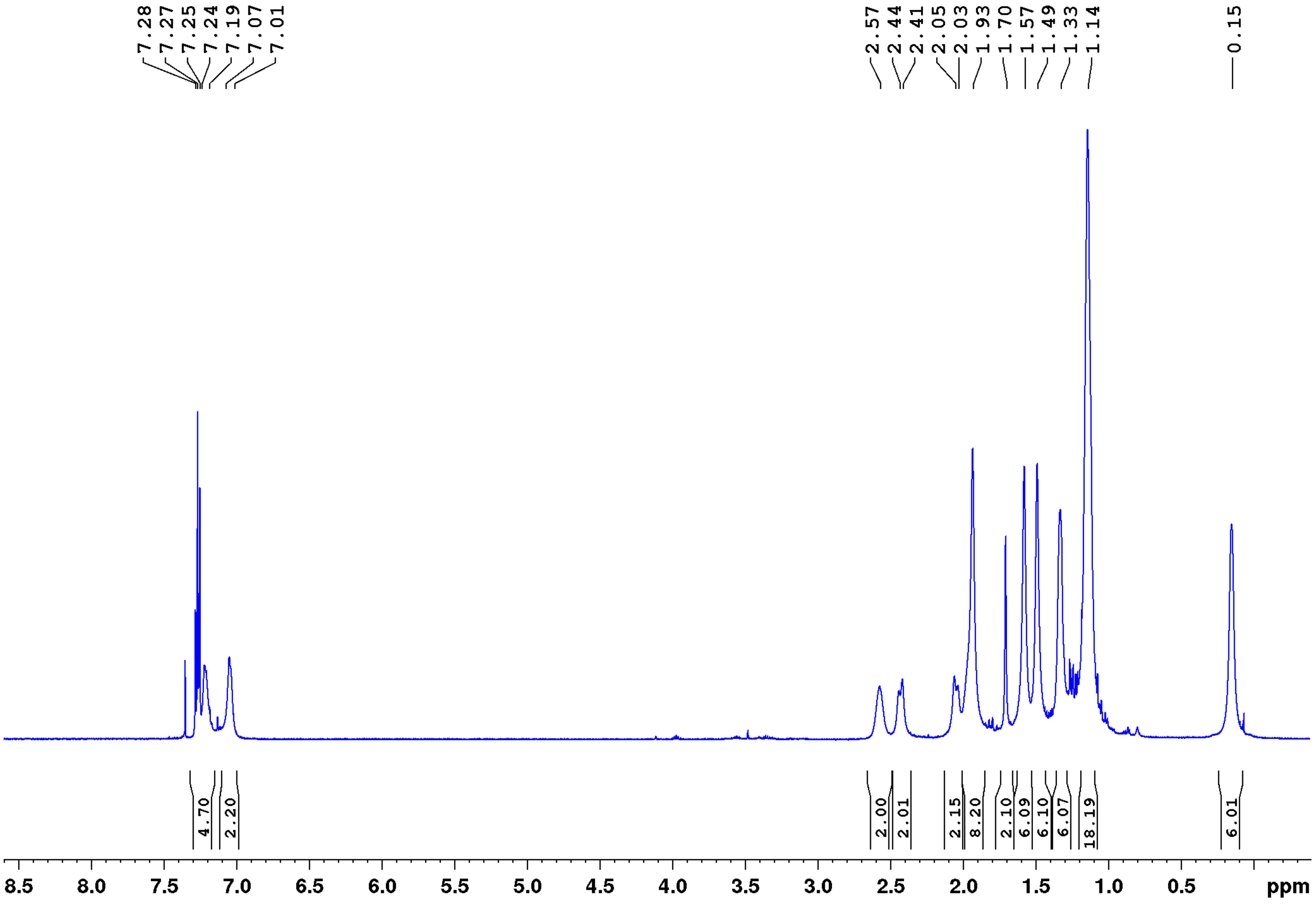

Figure S45. $^{13}C\{^{1}H\}$ NMR spectrum of **8** in $CDCl_3$.

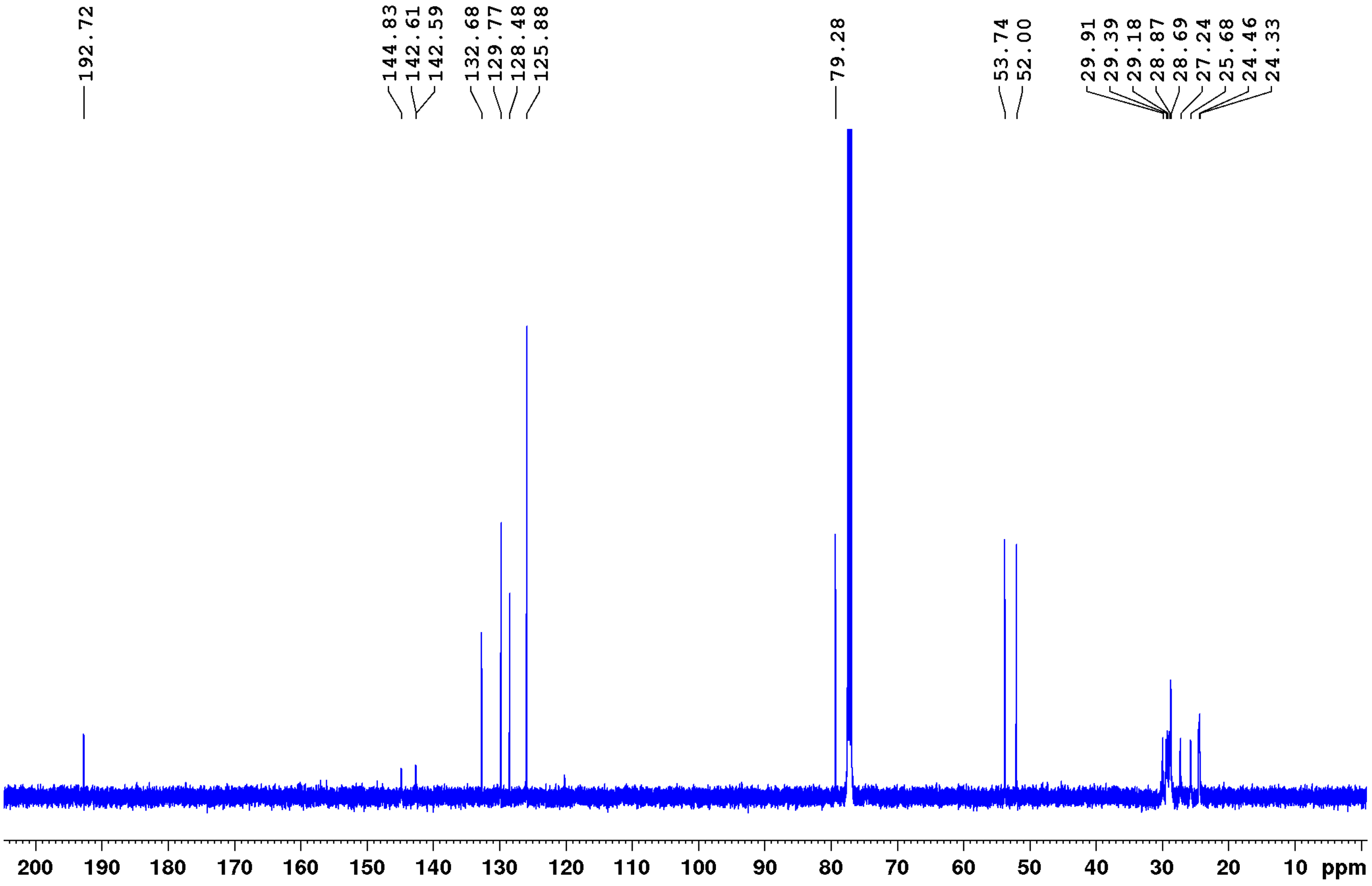

Figure S46. ^{11}B NMR spectrum of **8** in $CDCl_3$.

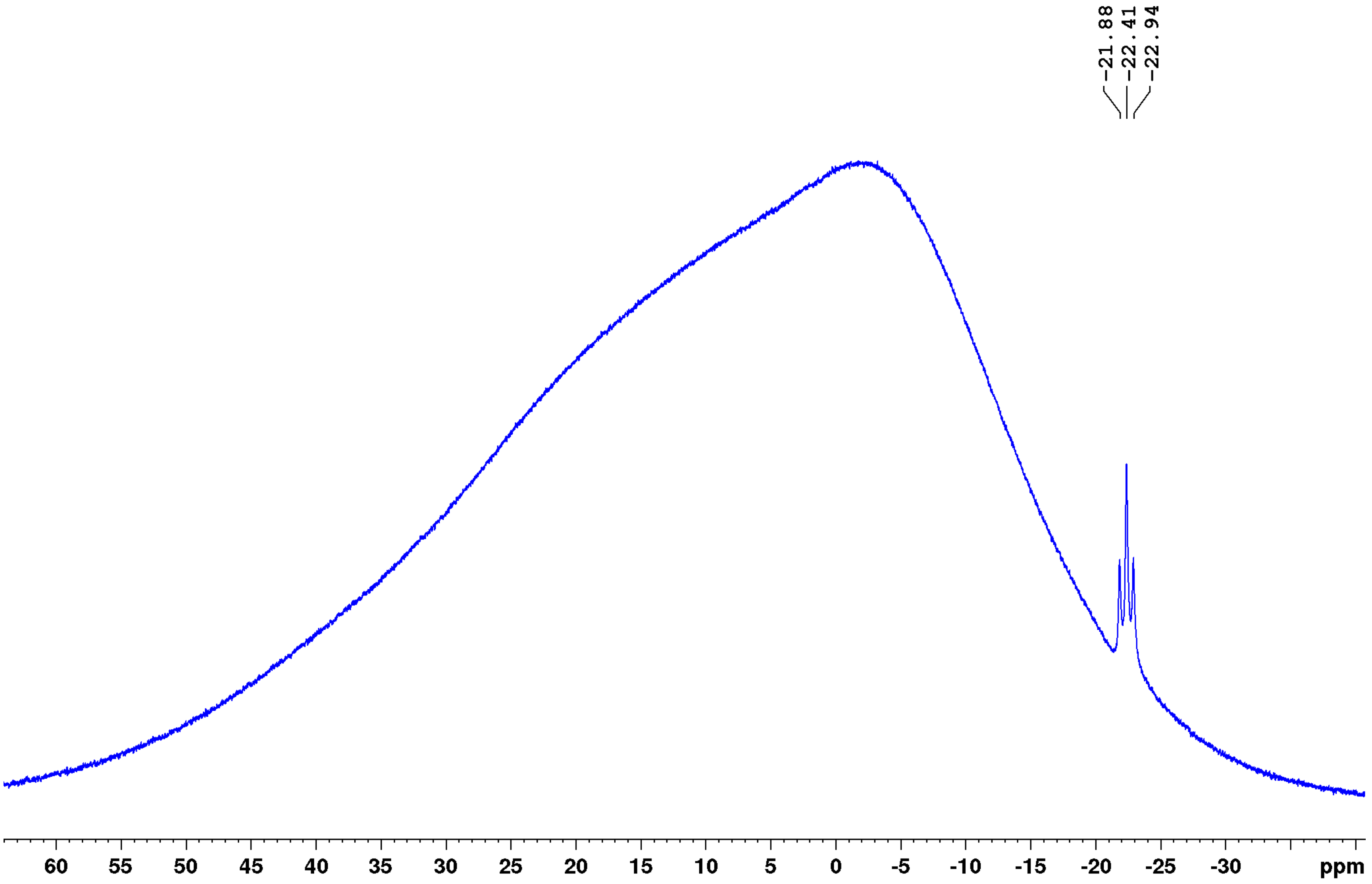

IR spectra

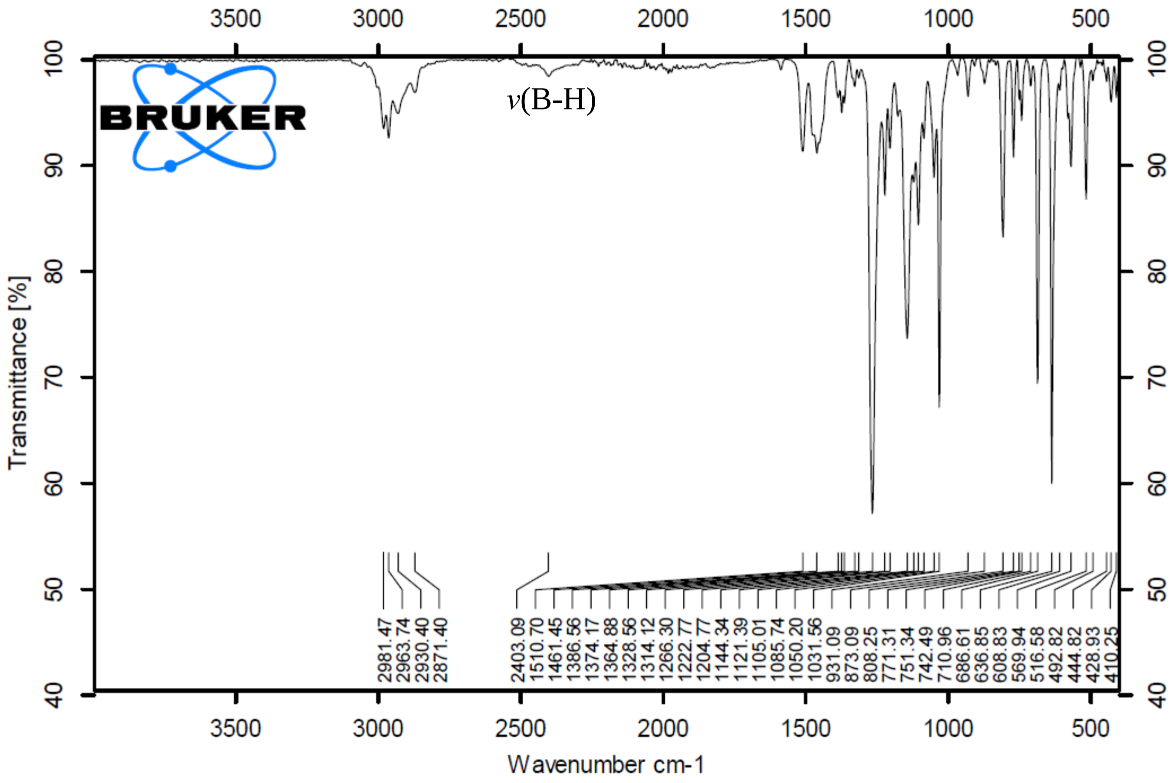

Figure S47. Solid state IR spectrum of **2-CAAC$^{\mathbf{Me}}$**.

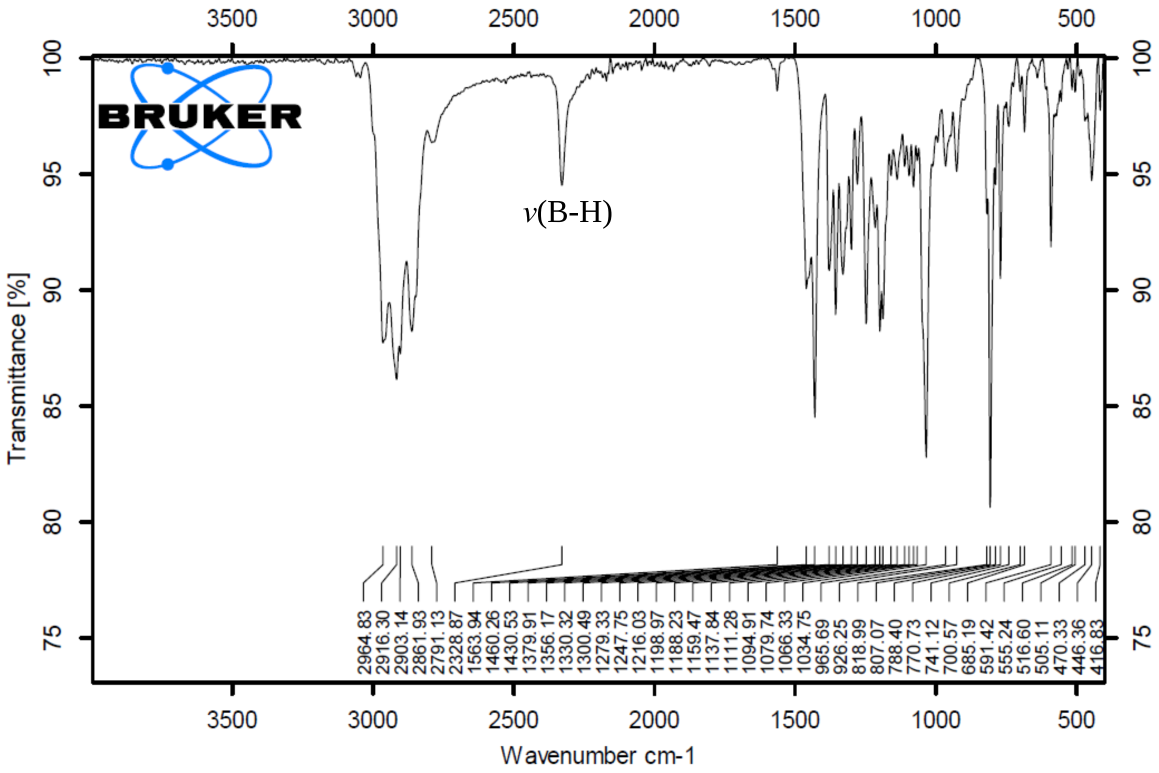

Figure S48. Solid state IR spectrum of **5**.

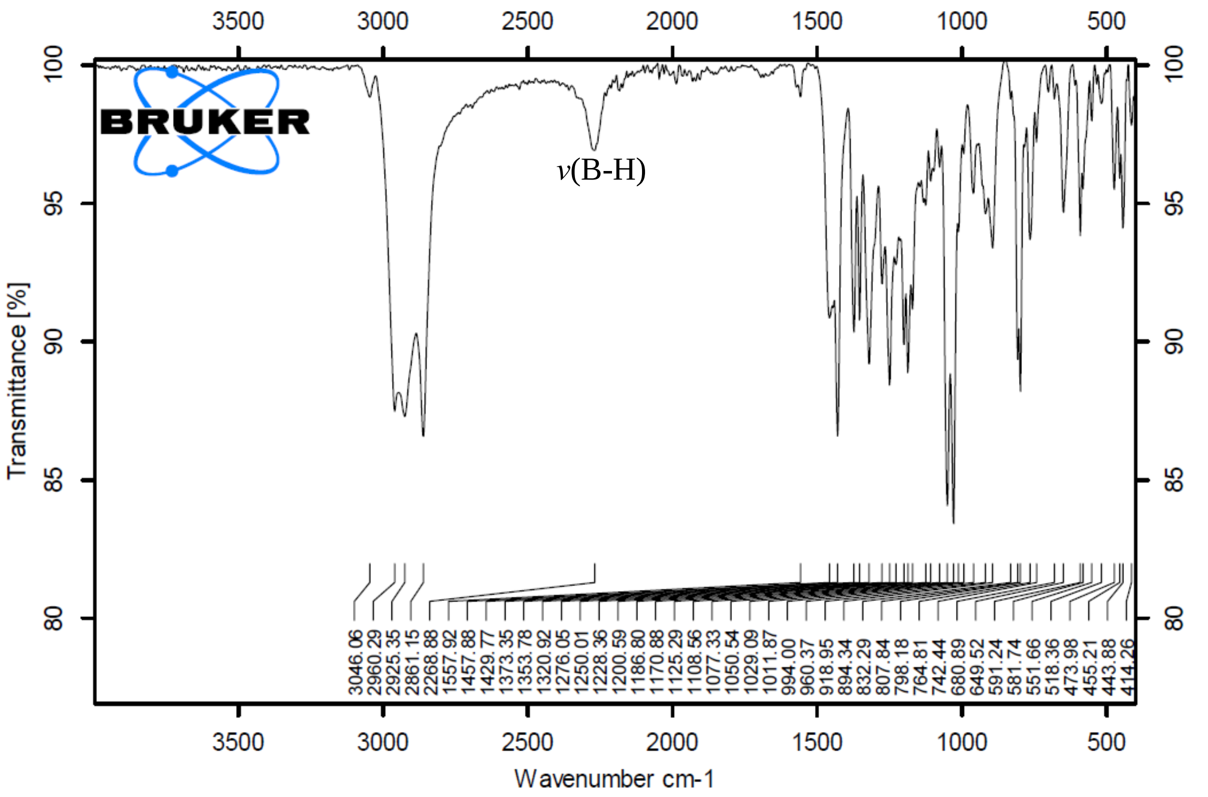

Figure S49. Solid state IR spectrum of **5-thf**.

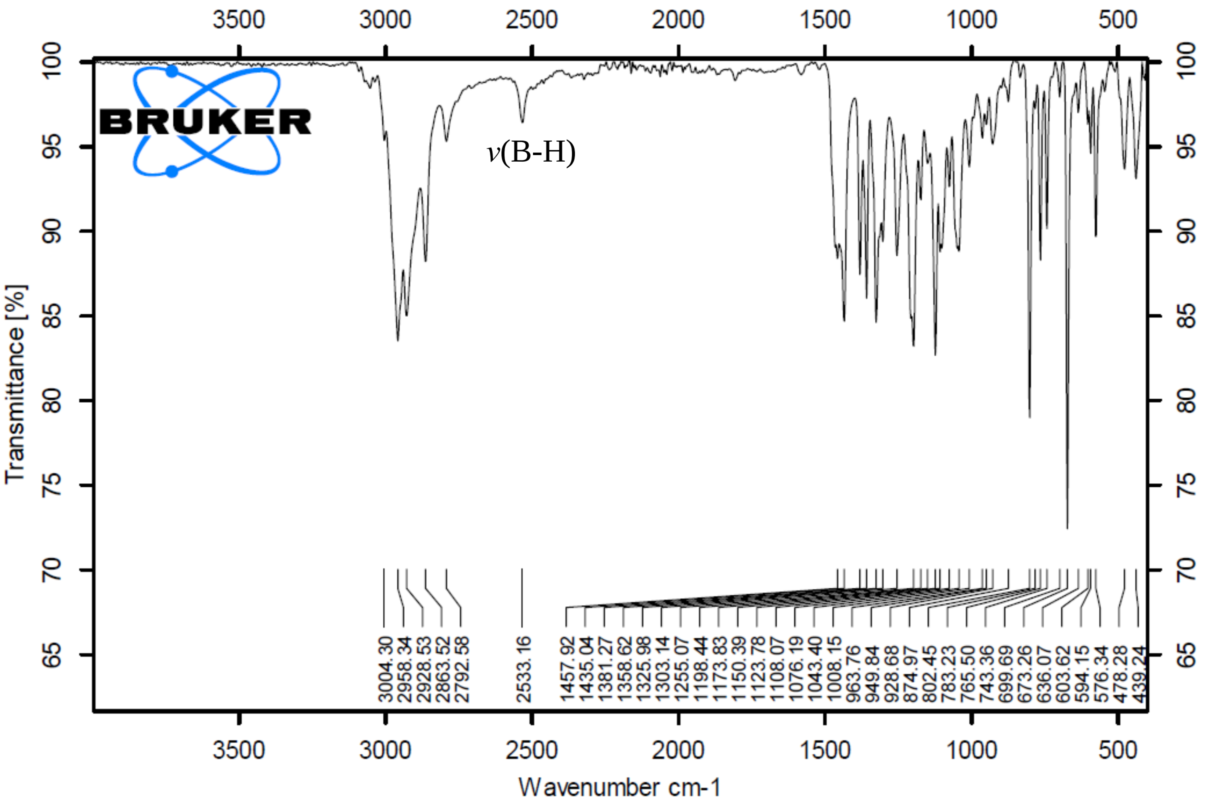

Figure S50. Solid state IR spectrum of **6**.

UV-vis spectra

UV-vis spectra were measured on a JASCO V-660 UV-vis spectrometer inside a glovebox.

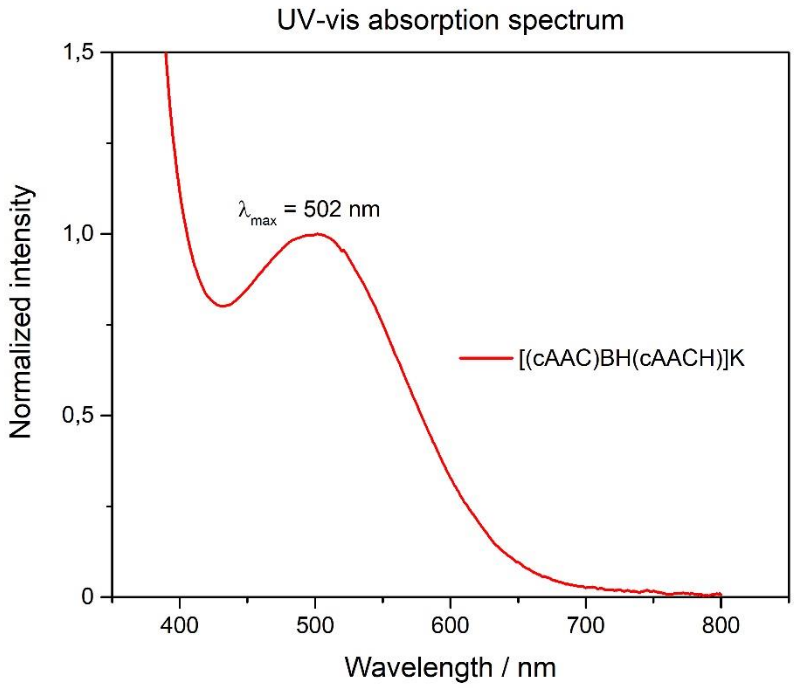

Figure S51. UV-vis absorption spectrum of the boryl anion **5-thf** in THF at 25 °C.

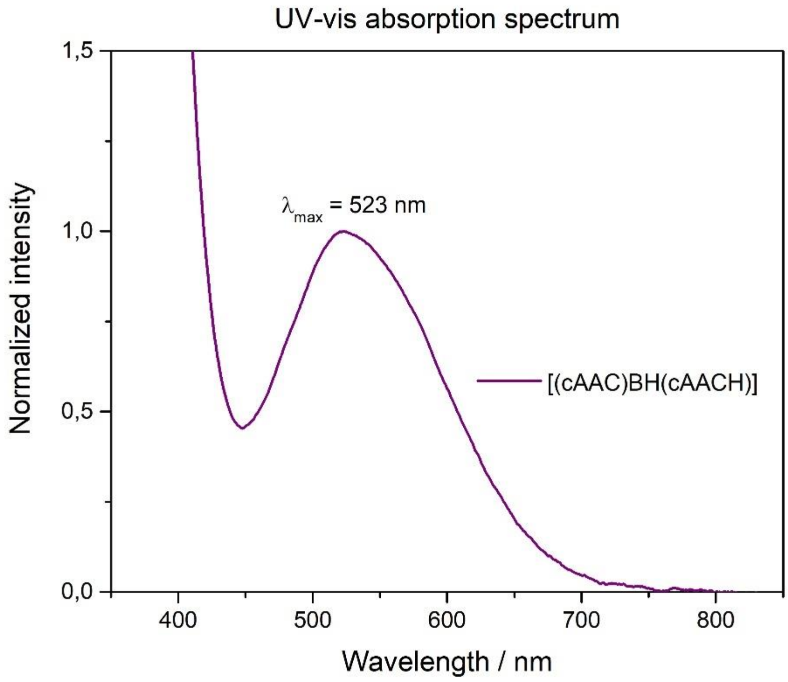

Figure S52. UV-vis absorption spectrum of the boryl radical **6** in benzene at 25 °C.

Cyclic voltammetry

Cyclic voltammetry experiments were performed using a Gamry Instruments Reference 600 potentiostat inside a glovebox. A standard three-electrode cell configuration was employed using a platinum disk working electrode, a platinum wire counter electrode, and a silver wire, separated by a *Vycor* tip, serving as the reference electrode. Formal redox potentials are referenced to the ferrocene/ferrocenium ($[Cp_2Fe]^{+/0}$) redox couple by using ferrocene as an internal standard. Tetra-*n*-butylammonium hexafluorophosphate ($[nBu_4N][PF_6]$) was employed as the supporting electrolyte. Compensation for resistive losses (*iR* drop) was employed for all measurements.

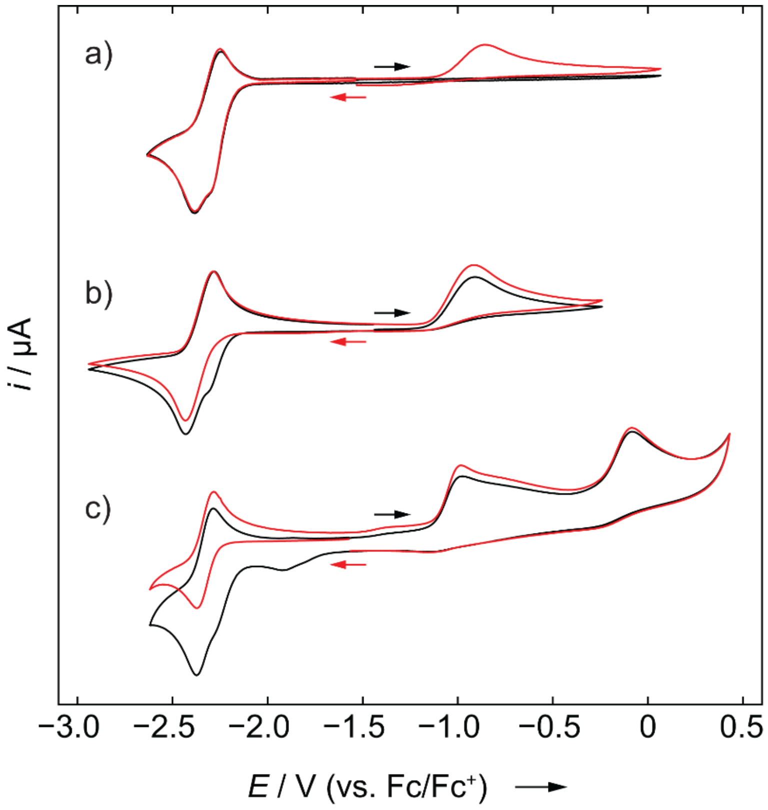

Figure S53. Cyclic voltammograms of a) **2-CAAC^{Me}**, b) the boryl radical **6** and c) the boryl anion **5-thf** in THF/0.1 M $[n\text{-}Bu_4N][PF_6]$ measured at 250 mV s^{-1}. The voltammetric response for the positive (black) and negative (red) scan direction is shown for each of the compounds. Formal potentials: $E_{1/2} = -2.31$ V (shoulder preceding the reduction: −2.28 V), E_{pa1} = ca. −0.90 V, and $E_{pa2} = -0.10$ V (corresponding reduction peak at −1.93 V; relative to the Fc/Fc^{+} couple).

EPR measurement

EPR measurements at X-band (9.86 GHz) were carried out at room temperature using a Bruker ELEXSYS E580 CW EPR spectrometer. CW EPR spectra were measured using 1 mW microwave power and 0.5 G field modulation at 100 kHz, with a conversion time of 20 ms. The spectral simulations were performed using MATLAB 8.6 and the EasySpin 5.2.25 toolbox.[4]

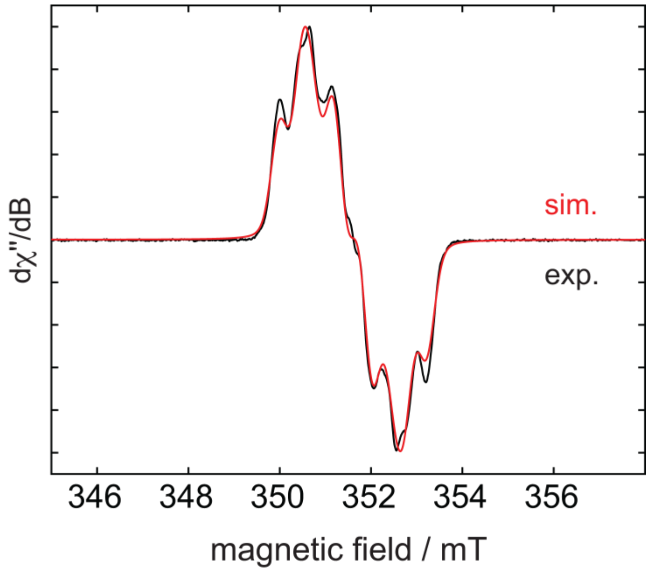

Figure S54. Experimental (black solid line) and simulated (red dashed line) continuous-wave X-band EPR spectra of **6** in hexane solution at room temperature. *Simulation parameters*: g_{iso} = 2.0027, $a(^{11}B)$ = 9.7 MHz (0.35 mT), $a(^{14}N)$ = 18.5 MHz (0.66 mT), $a(^{1}H)$ = 13.6 MHz (0.49 mT), and $a(^{1}H)$ = 4.8 MHz (0.17 mT).

X-ray crystallographic data

The crystal data of all compounds were collected on a Bruker X8-APEX II diffractometer with a CCD area detector (compounds **1**, **2-CAAC$^{\mathbf{Me}}$** and **3-DMAP**) or a Bruker D8 QUEST diffractometer with a CMOS area detector (compounds **2-Pyr**, **2-DMAP**, **5**, **5-thf** and **6**), each equipped with *m*-layer mirror monochromated $Mo_{K\alpha}$ radiation. The structures were solved using intrinsic phasing methods,[5] refined with the SHELXL program[6] and expanded using Fourier techniques. All non-hydrogen atoms were refined anisotropically. Hydrogen atoms were refined isotropically and assigned to idealised positions, except for boron-bound hydrogen atoms, which were located in the difference Fourier map and freely refined.

All crystallographic data have been submitted as cif files with the Cambridge Crystallographic Data Centre, CCDC numbers 1956847 (**2-Pyr**), 1956848 (**2-DMAP**), 1956849 (**2-CAAC$^{\mathbf{Me}}$**), 1956850 (**3-DMAP**), 1956851 (**5-thf**), 1956852 (**6**), 1956853 (**1**) and 1956854 (**5**). These data can be obtained free of charge from The Cambridge Crystallographic Data Centre *via* www.ccdc.cam.ac.uk/data_request/cif.

Crystal data for 1: Formula: $C_{21}H_{33}BF_3NO_3S$, $M_r = 447.35$, colourless block, 0.431×0.231×0.225 mm^3, monoclinic space group $P2_1/n$, $a = 8.2118(7)$ Å, $b = 16.9590(13)$ Å, $c = 16.6506(12)$ Å, $\beta = 95.196(3)°$, $V = 2309.3(3)$ $Å^3$, $Z = 4$, $\rho_{calcd} = 1.287$ $g{\cdot}cm^{-3}$, $\mu = 0.186$ mm^{-1}, $F(000) = 952$, $T = 102(2)$ K, $R_1 = 0.0523$, $wR^2 = 0.0889$, 4544 independent reflections [$2\theta \leq 52.034°$] and 287 parameters.

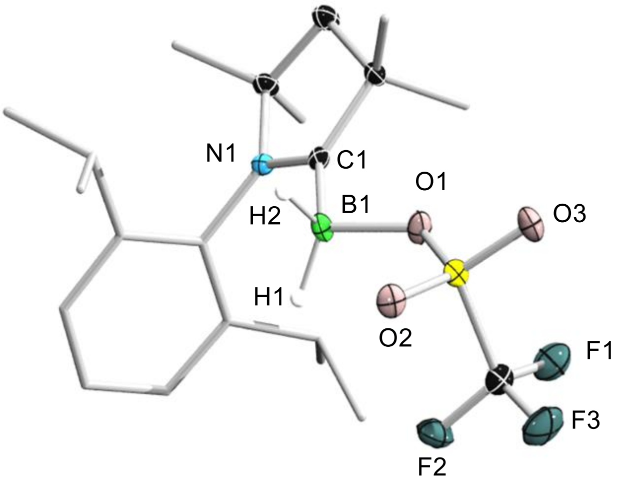

Figure S55. Crystallographically-derived molecular structure of compound **1**. Atomic displacement ellipsoids drawn at 50% probability level. Ellipsoids on the $CAAC^{Me}$ ligand periphery and hydrogen atoms omitted for clarity. Selected bond lengths (Å): N1–C1 1.300(2), C1–B1 1.598(2), B1–O1 1.562(2).

Refinement details for 2-CAAC^{Me}: The asymmetric unit contains two sets of the borate with its triflate anion and a chloroform solvent molecule. Only one triflate (RESI OTfl) and one chloroform residue (RESI CLF) are twofold disordered in a 4:1 ratio, which leads to a non-centrosymmetric space group. Distances within the parts were restrained with SAME, ADPs with SIMU 0.005. Two reflections affected by the beamstop were omitted.

Crystal data for 2-CAAC^{Me}: Formula: $C_{42}H_{65}BCl_3F_3N_2O_3S$, $M_r = 852.18$, colorless block, 0.376×0.155×0.127 mm^3, monoclinic space group $P2_1$, $a = 9.1335(4)$ Å, $b = 22.0651(8)$ Å, $c = 21.9639(8)$ Å, $\beta = 94.994(2)°$, $V = 4409.6(3)$ $Å^3$, $Z = 4$, $\rho_{calcd} = 1.284$ $g{\cdot}cm^{-3}$, $\mu = 0.307$ mm^{-1}, $F(000) = 1816$, $T = 102(2)$ K, $R_1 = 0.0893$, $wR^2 = 0.1403$, 18321 independent reflections [$2\theta \leq 54.206°$] and 1148 parameters.

Refinement details for 2-Pyr: The triflate counteranion was modelled as threefold disordered, once by rotation around the SC axis, once by centrosymmetry + rotation. The parts were refined with 3 FVAR summed to 1 in a 42:41:17 ratio. The bond distances in these parts were equalised with the SAME keyword and the ADPs restrained with SIMU 0.003.

Crystal data for 2-Pyr: Formula: $C_{26}H_{38}BF_3N_2O_3S$, $M_r = 526.45$, colourless plate, 0.337×0.14×0.071 mm^3, triclinic space group $P\bar{1}$, $a = 10.1404(6)$ Å, $b = 10.7992(7)$ Å, $c = 14.1434(10)$ Å, $\alpha = 109.794(2)°$, $\beta = 104.440(2)°$, $\gamma = 99.480(2)°$, $V = 1357.49(15)$ Å^3, $Z = 2$, $\rho_{calcd} = 1.288$ g·cm^{-3}, $\mu = 0.170$ mm^{-1}, $F(000) = 560$, $T = 101(2)$ K, $R_1 = 0.0786$, $wR^2 = 0.1212$, 5991 independent reflections [$2\theta \leq 54.276°$] and 488 parameters.

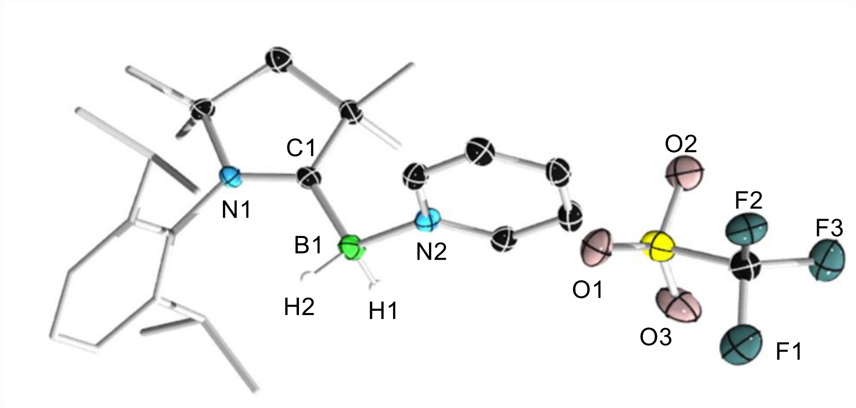

Figure S56. Crystallographically-derived molecular structure of **2-Pyr** (only one part of the disordered triflate anion shown). Atomic displacement ellipsoids drawn at 50% probability level. Ellipsoids on the CAAC^{Me} ligand periphery and hydrogen atoms except H1 and H2 omitted for clarity. Selected bond lengths (Å): N1–C1 1.302(2), C1–B1 1.608(3), B1–N2 1.589(2),

Refinement details for 2-DMAP: The triflate counteranion was modelled as twofold disordered by rotation around the C-S axis. The parts were refined with FVAR to a 56:44 ratio. The ADPs within these residues were restrained to the same value with similarity restraint SIMU 0.002 and the 1-2 and 1-3 distances to the same values with SAME.

Crystal data for 2-DMAP: Formula: $C_{28}H_{43}BF_3N_3O_3S$, $M_r = 569.52$, colorless block, 0.59×0.38×0.206 mm^3, triclinic space group $P\bar{1}$, $a = 10.6577(12)$ Å, $b = 10.6616(15)$ Å, $c = 13.7408(18)$ Å, $\alpha = 88.486(6)°$, $\beta = 79.875(5)°$, $\gamma = 81.424(6)°$, $V = 1519.8(3)$ Å^3, $Z = 2$,

ρ_{calcd} = 1.244 g·cm^{-3}, μ = 0.158 mm^{-1}, F(000) = 608, T = 101(2) K, R_1 = 0.0926, wR^2 = 0.1409, 7144 independent reflections [2θ≤55.722°] and 443 parameters.

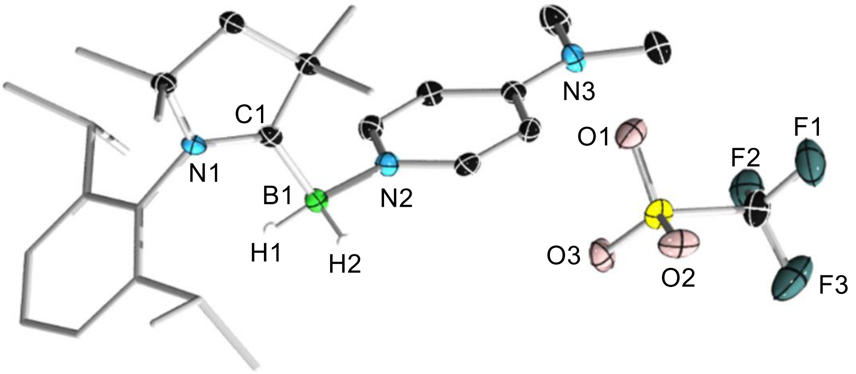

Figure S57. Crystallographically-derived molecular structure of **2-DMAP**. Atomic displacement ellipsoids drawn at 50% probability level. Ellipsoids on the CAAC^{Me} ligand periphery and hydrogen atoms except H1 and H2 omitted for clarity. Selected bond lengths (Å): N1–C1 1.308(3), C1–B1 1.616(3), B1–N2 1.574(3).

Refinement details for 3-DMAP: The asymmetric unit contains one undisordered THF and a second one which was manifold disordered and eluded modelling. The latter was removed using Squeeze/Platon,[7] treating it as a diffuse contribution to the overall scattering without specific atom positions. The number of electrons squeezed per unit cell is 74, which corresponds to 1.85 THF molecules (40 electrons), i.e. 0.93 THF per asymmetric unit.

Crystal data for 3-DMAP: Formula: $C_{43}H_{69}BF_3N_5O_5S$, M_r = 835.90, colorless block, 0.436×0.286×0.256 mm^3, triclinic space group $P\,\bar{1}$, a = 8.7125(7) Å, b = 12.5555(11) Å, c = 22.1845(19) Å, α = 78.804(3)°, β = 81.554(3)°, γ = 73.644(3)°, V = 2273.2(3) Å^3, Z = 2, ρ_{calcd} = 1.221 g·cm^{-3}, μ = 0.131 mm^{-1}, F(000) = 900, T = 102(2) K, R_1 = 0.0996, wR^2 = 0.1470, 9562 independent reflections [2θ≤52.044°] and 494 parameters.

Refinement details for 5: All Hydrogen atoms except the boron-bound H1 were assigned to idealised positions. H1 was detected in the Fourier difference map and freely refined. The structure is twofold disordered by a C_2 rotation around the axis passing through K1 and the

center of B1···B1'. The ensuing disorder in B1, the carbene C1A and the protonated C1B was modelled with part –1 and occupation factor 0.5 and the ADPs of B1, C1A and C1B were restrained using SIMU 0.002.

Crystal data for 5: Formula: $C_{52}H_{76}BKN_2$, M_r = 779.05, orange block, 0.277×0.23×0.098 mm^3, monoclinic space group *C*2/*c*, *a* = 22.753(9) Å, *b* = 9.854(5) Å, *c* = 20.638(7) Å, β = 93.074(15)°, *V* = 4621(4) Å^3, *Z* = 4, ρ_{calcd} = 1.120 g·cm^{-3}, μ = 0.151 mm^{-1}, *F*(000) = 1704, *T* = 102(2) K, R_1 = 0.0698, wR^2 = 0.1073, 4914 independent reflections [2θ≤53.506°] and 279 parameters.

Refinement details for 5-thf: All hydrogen atoms except the boron-bound H1 were assigned to idealised positions. H1 was detected in the Fourier difference map and freely refined. Two of the potassium-bound THF residues were modelled as twofold disordered in a 63:37 ratio. The displacement parameters of the atoms of these residues were restrained using SIMU 0.003.

Crystal data for 5-thf: Formula: $C_{52}H_{88}BKN_2O_3$, M_r = 839.15, red block, 0.338×0.196×0.08 mm^3, triclinic space group $P\bar{1}$, *a* = 10.337(2) Å, *b* = 12.573(3) Å, *c* = 20.686(6) Å, α = 106.736(9)°, β = 90.380(17)°, γ = 97.258(10)°, *V* = 2551.4(11) Å^3, *Z* = 2, ρ_{calcd} = 1.092 g·cm^{-3}, μ = 0.145 mm^{-1}, *F*(000) = 924, *T* = 102(2) K, R_1 = 0.0592, wR^2 = 0.1075, 10054 independent reflections [2θ≤52.042°] and 622 parameters.

Crystal data for 6: Formula: $C_{43}H_{67}BN_2$, M_r = 622.79, orange block, 0.449×0.418×0.326 mm^3, triclinic space group $P\bar{1}$, *a* = 9.1257(3) Å, *b* = 11.7040(3) Å, *c* = 18.4845(5) Å, α = 97.5860(10)°, β = 103.7760(10)°, γ = 91.7560(10)°, *V* = 1896.75(9) Å^3, *Z* = 2, ρ_{calcd} = 1.090 g·cm^{-3}, μ = 0.061 mm^{-1}, *F*(000) = 688, *T* = 100(2) K, R_1 = 0.0536, wR^2 = 0.1215, 8317 independent reflections [2θ≤54.288°] and 435 parameters.

Computational details

Initially, we performed geometry optimisation calculations on the boronium cation **2-CAAC^{Me}**, the (alkyl)hydroboryl anion **5** with potassium as a counterion, and the boryl radical **6** starting from the experimental crystallographic data. All structures were optimised using density functional theory (DFT) with the ωB97XD[8] functional and 6-31+G* basis set. Stability tests[9,10] on the single-determinant wave functions at the optimised geometries were performed for **2-CAAC^{Me}** and **5**. We did not observe internal instability for the singlet closed-shell solution of any of the systems, revealing that they do not present diradical character. The optimised structures were only slightly different from the X-ray diffraction data. We performed Hessian calculations and thermochemical analyses at the same level of theory. All geometries were characterised as minimum energy structures in their respective potential energy surfaces by vibrational frequency analysis, as all Hessian eigenvalues are positive. From the optimised structure of **6**, we obtained the electrostatic potential (ESP)[11] charges and built the respective ESP map. Single-point calculations at the ωB97XD/6-311++G** level of theory starting from the optimised structures were obtained for generating molecular orbital diagrams for **2-CAAC^{Me}**, **5**, and **6**. All calculations were carried out with Gaussian 16, RevB.01.[12] Pictures of molecular structures, orbitals and densities were generated with ADFView.[13]

Geometrical Parameters

Table S1. Comparison of the geometrical parameters of the X-ray and the computed equilibrium structures of **2-CAAC^{Me}**.

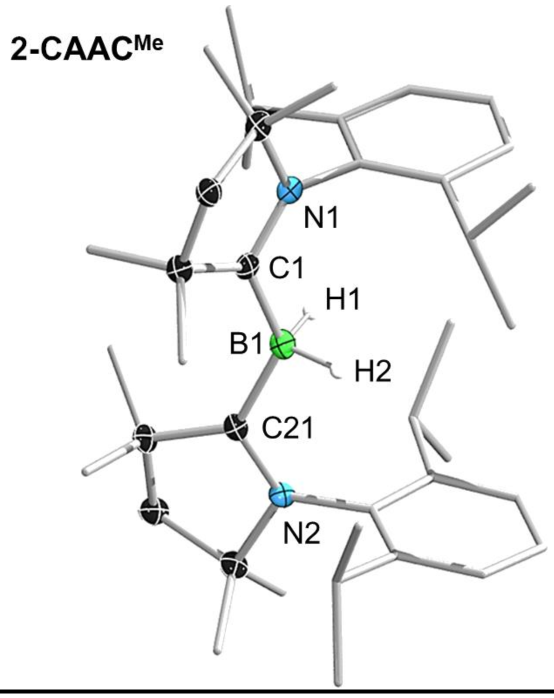

	**2-CAAC^{Me} (X-ray)**	**2-CAAC^{Me} (Calc)**
B1–C1	1.597(7)	1.604
B1–C21	1.607(7)	1.605
B1–H1	1.11(6)	1.205
B1–H2	1.16(6)	1.210
C1–N1	1.316(6)	1.311
C21-N2	1.310(6)	1.311

Table S2. Comparison of the geometrical parameters of the X-ray and the computed equilibrium structures of **5**.

5

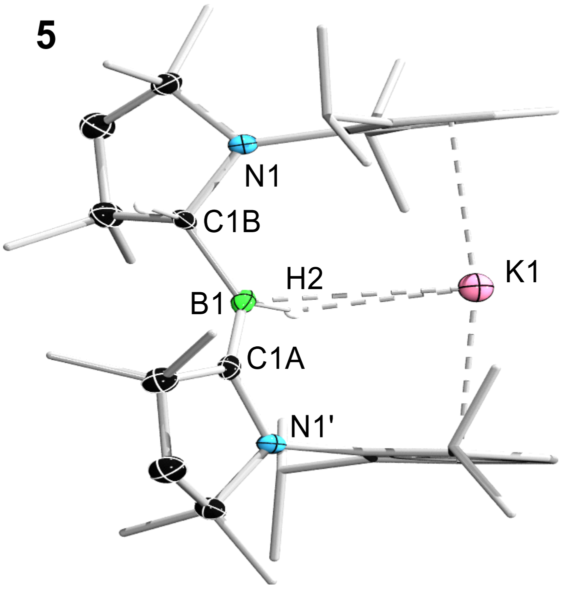

	5 (X-ray)	5 (Calc)
B1–C1A	1.439(11)	1.463
B1–C1B	1.633(9)	1.619
B1–H2	1.14(3)	1.222
C1A–N1'	1.450(7)	1.456
C1B–N1	1.520(8)	1.487
K1···H2	2.53(3)	2.504
K1···B1	3.141(4)	3.072
K1···Centroid	2.91	2.89
Σ∠B1	359.4(12)	359.4
Σ∠C1A	359.7(5)	359.9
B1–H2–K1	111.8(12)	105.8

Table S3. Comparison of the geometrical parameters of the X-ray and the computed equilibrium structures of **6**.

6

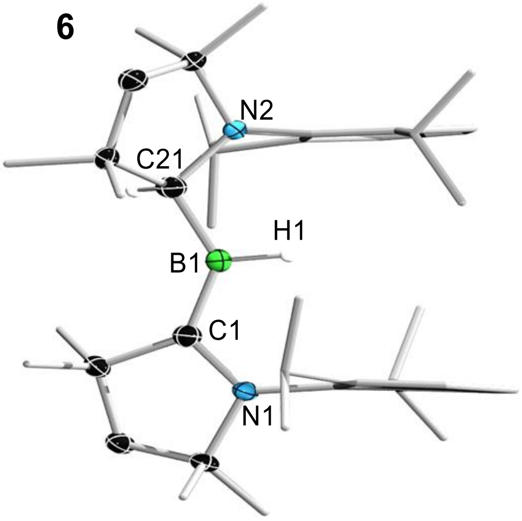

	6 (X-ray)	6 (Calc)
B1–C1	1.5174(18)	1.519
B1–C21	1.5817(18)	1.608
B1–H1	1.142(18)	1.194
C1–N1	1.3777(15)	1.377
C21–N2	1.4616(15)	1.475
Σ∠B1	359.5(6)	360.0
Σ∠C1	359.6(1)	360.0

Hessian Calculations and Thermochemical Analysis

Table S4. Harmonic frequencies (cm^{-1}), reduced masses (amu), force constants (mDyne $Å^{-1}$) and IR intensities (km mol^{-1}) for the nine lowest vibrational frequency modes of **2-CAAC^{Me}**, **5** and **6**.

Vibrational Mode	Frequencies	Reduced Masses	Force Constants	IR Intensities
**2-CAAC^{Me}**				
1	16.8774	3.1671	0.0005	0.2143
2	38.0054	4.1664	0.0035	0.0237
3	55.4737	3.0811	0.0056	0.2818
4	62.7797	3.6650	0.0085	0.0381
5	65.3563	3.6869	0.0093	0.0176
6	68.7469	3.2618	0.0091	0.5293
7	74.9632	3.5585	0.0118	0.0354
8	82.5177	3.9514	0.0159	0.1458
9	91.9349	3.4713	0.0173	0.0871
**5**				
1	28.2596	4.6444	0.0022	0.7828
2	45.0035	4.0902	0.0049	0.0493
3	52.2714	3.5702	0.0057	0.0539
4	60.4982	3.6057	0.0078	0.4352
5	68.4855	4.6503	0.0129	0.3683
6	72.4202	4.0898	0.0126	1.1930
7	81.0868	4.1441	0.0161	1.2788
8	81.6011	3.6233	0.0142	0.5029
9	90.7557	4.0427	0.0196	1.2940
**6**				
1	25.3932	3.4769	0.0013	0.0071
2	39.8043	4.1377	0.0039	0.0047
3	55.4558	3.4560	0.0063	0.0115
4	59.0113	3.9747	0.0082	0.0321
5	63.7202	3.7542	0.0090	0.0378
6	67.1481	3.6917	0.0098	0.1045
7	75.1371	3.4368	0.0114	0.2024
8	84.7436	4.2425	0.0180	0.2014
9	92.8438	3.5415	0.0180	0.3177

Table S5. Thermochemical analysis of **2-CAAC**$^{\mathbf{Me}}$, **5**, and **6**. All values are in Hartree.

	**2-CAAC**$^{\mathbf{Me}}$	**5**	**6**
**ωB97XD/6-31+G***			
Electronic energy	-1696.6010	-2296.6876	-1696.7552
Zero-point correction	0.9799	0.9782	0.9779
Thermal correction to energy	1.0260	1.0256	1.0239
Thermal correction to enthalpy	1.0270	1.0265	1.0249
Thermal correction to Gibbs free energy	0.9081	0.9055	0.9059
Sum of electronic and zero-point energies	-1695.6211	-2295.7094	-1695.7773
Sum of electronic and thermal energies	-1695.5750	-2295.6620	-1695.7313
Sum of electronic and thermal enthalpies	-1695.5741	-2295.6611	-1695.7303
Sum of electronic and thermal free energies	-1695.6929	-2295.7821	-1695.8493
**ωB97XD/6-311++G**//ωB97XD/6-31+G***			
Electronic energy	-1696.9732	-2297.0991	-1697.1292

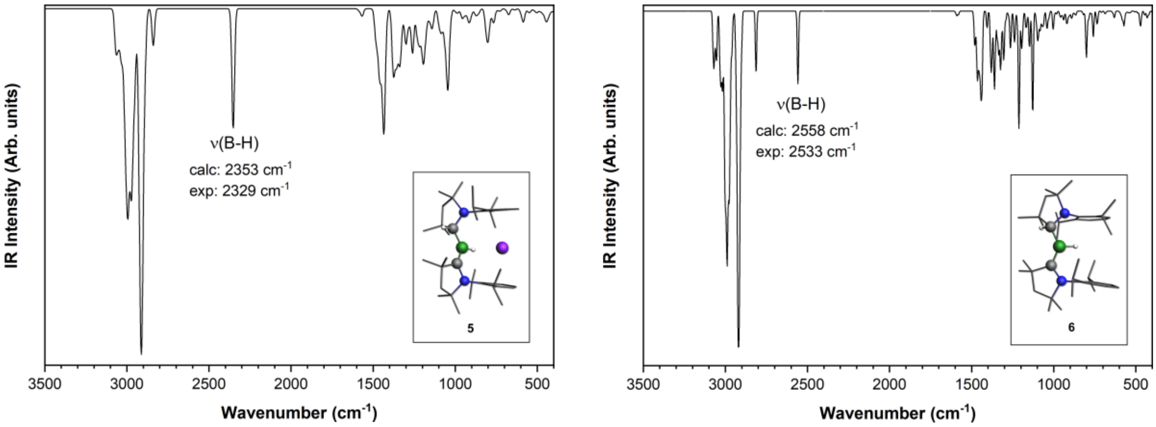

Figure S58. Computed IR spectrum of **5** and **6** at the ωB97XD/6-31+G* level of theory. A precomputed vibrational scaling factor of 0.952 was used for the frequencies.[14]

Table S6. Cartesian Coordinates (ωB97XD/6-31+G*)

2-CAAC^{Me}

B	-0.018245000	-0.641894000	-0.061621000
H	0.227921000	0.246554000	0.714313000
H	-0.323503000	-0.111498000	-1.105618000
N	-2.431279000	-0.516270000	0.593962000
N	2.448195000	-0.574222000	-0.529342000
C	-1.348725000	-1.255210000	0.593329000
C	-3.601549000	-1.079700000	1.377748000
C	-4.226442000	-0.049604000	2.312316000
H	-5.048574000	-0.533725000	2.849182000
H	-4.639090000	0.802184000	1.764692000
H	-3.513332000	0.310472000	3.055396000
C	-4.686937000	-1.578138000	0.421858000
H	-5.472801000	-2.067783000	1.004634000
H	-4.314532000	-2.294407000	-0.312980000
H	-5.141314000	-0.737901000	-0.109083000
C	-2.905691000	-2.195398000	2.165571000
H	-2.679547000	-1.839875000	3.176522000
H	-3.544748000	-3.076849000	2.265662000
C	-1.595405000	-2.509980000	1.417166000
C	-1.811838000	-3.718309000	0.484005000
H	-2.046490000	-4.597122000	1.093479000
H	-0.934825000	-3.954403000	-0.114249000
H	-2.646158000	-3.555931000	-0.203870000
C	-0.446247000	-2.763320000	2.399066000
H	-0.617041000	-3.701527000	2.937939000
H	-0.384234000	-1.957740000	3.135490000
H	0.523171000	-2.827261000	1.899265000
C	-2.590644000	0.740080000	-0.120979000
C	-3.007555000	0.711037000	-1.467446000
C	-3.042001000	-0.546695000	-2.328475000
H	-2.777509000	-1.406651000	-1.704684000

C	-1.985201000	-0.449141000	-3.443511000
H	-1.916615000	-1.400684000	-3.982675000
H	-0.998399000	-0.203681000	-3.042646000
H	-2.254212000	0.325512000	-4.169924000
C	-4.415360000	-0.814687000	-2.961822000
H	-4.395481000	-1.762768000	-3.510261000
H	-4.678273000	-0.029572000	-3.678670000
H	-5.217947000	-0.867695000	-2.223020000
C	-3.308814000	1.929733000	-2.078005000
H	-3.642517000	1.937683000	-3.111460000
C	-3.172539000	3.132594000	-1.401015000
H	-3.421491000	4.067416000	-1.894019000
C	-2.677598000	3.137705000	-0.105826000
H	-2.520163000	4.086484000	0.398255000
C	-2.361945000	1.952025000	0.561342000
C	-1.719528000	2.061175000	1.939718000
H	-1.574505000	1.050674000	2.338412000
C	-2.572439000	2.866606000	2.934251000
H	-2.151734000	2.781002000	3.942057000
H	-3.613701000	2.538093000	2.968093000
H	-2.573705000	3.930589000	2.674604000
C	-0.330672000	2.714550000	1.836600000
H	0.156728000	2.719060000	2.817781000
H	-0.410980000	3.754047000	1.499402000
H	0.320369000	2.187909000	1.137710000
C	1.364075000	-1.310536000	-0.526646000
C	3.651607000	-1.191958000	-1.216121000
C	4.344372000	-0.222574000	-2.168433000
H	5.184914000	-0.747994000	-2.633173000
H	4.741450000	0.653922000	-1.648954000
H	3.677873000	0.104458000	-2.967757000
C	4.669306000	-1.651361000	-0.169431000
H	5.489394000	-2.169036000	-0.675785000
H	4.243265000	-2.333353000	0.569395000

H	5.091367000	-0.790238000	0.355002000
C	2.982634000	-2.338705000	-1.980183000
H	3.617920000	-3.227949000	-2.009705000
H	2.805474000	-2.029670000	-3.016009000
C	1.635562000	-2.608155000	-1.278672000
C	0.527632000	-2.877466000	-2.305001000
H	0.730707000	-3.816021000	-2.831965000
H	0.483411000	-2.074175000	-3.046392000
H	-0.460041000	-2.949386000	-1.842962000
C	1.791443000	-3.794144000	-0.306467000
H	2.080507000	-4.681234000	-0.879309000
H	0.873696000	-4.032368000	0.226246000
H	2.570141000	-3.610814000	0.439382000
C	2.593979000	0.738473000	0.077554000
C	2.401303000	1.883882000	-0.718933000
C	1.767746000	1.864351000	-2.104414000
H	1.649420000	0.821385000	-2.419871000
C	0.361980000	2.486446000	-2.048244000
H	-0.129771000	2.399413000	-3.023562000
H	0.417064000	3.551346000	-1.795105000
H	-0.269451000	1.998496000	-1.304453000
C	2.606210000	2.602691000	-3.159965000
H	2.193148000	2.422402000	-4.158300000
H	3.654631000	2.293740000	-3.160688000
H	2.583736000	3.684839000	-2.993078000
C	2.730501000	3.120461000	-0.160565000
H	2.599123000	4.021069000	-0.752776000
C	3.206409000	3.225847000	1.138367000
H	3.468941000	4.197358000	1.545990000
C	3.300716000	2.089914000	1.928881000
H	3.611639000	2.188006000	2.964806000
C	2.981217000	0.826519000	1.427399000
C	2.934143000	-0.346439000	2.397112000
H	2.715912000	-1.259607000	1.834245000

C	1.777019000	-0.132234000	3.388027000
H	1.668395000	-1.005895000	4.040372000
H	0.832098000	0.036979000	2.863096000
H	1.967102000	0.738680000	4.025006000
C	4.242898000	-0.563962000	3.168211000
H	4.165591000	-1.462465000	3.789931000
H	4.455495000	0.276557000	3.837255000
H	5.103181000	-0.684388000	2.504954000

**5**			
B	-0.035383000	0.911024000	-0.141800000
H	-0.171558000	0.249876000	-1.160700000
C	-1.242027000	1.183319000	0.639490000
C	-1.474233000	2.037261000	1.918795000
C	-3.018609000	2.107263000	2.064078000
H	-3.362793000	1.397932000	2.828469000
H	-3.359624000	3.101816000	2.375259000
C	-3.595528000	1.688964000	0.700880000
C	-0.867654000	1.368265000	3.160074000
H	-1.123600000	1.921502000	4.075904000
H	0.223335000	1.331802000	3.077059000
H	-1.234386000	0.342743000	3.271985000
C	-0.913952000	3.466334000	1.859828000
H	-1.188243000	4.012911000	2.772858000
H	-1.307172000	4.023708000	1.005303000
H	0.175287000	3.467228000	1.789682000
C	-3.670018000	2.911365000	-0.240913000
H	-4.190685000	3.743965000	0.247655000
H	-4.211980000	2.676483000	-1.161347000
H	-2.664148000	3.246113000	-0.512990000
C	-4.989615000	1.077431000	0.822057000
H	-5.019129000	0.269549000	1.558462000
H	-5.337544000	0.676969000	-0.137366000
H	-5.700517000	1.847996000	1.140793000

C	-2.826983000	-0.493145000	-0.383158000
C	-3.045810000	-1.678324000	0.369655000
C	-3.222350000	-2.898345000	-0.292969000
H	-3.400753000	-3.803154000	0.284442000
C	-3.181030000	-2.974629000	-1.681900000
H	-3.323016000	-3.928289000	-2.184089000
C	-2.990110000	-1.812676000	-2.422266000
H	-2.986484000	-1.870893000	-3.507877000
C	-2.826203000	-0.570444000	-1.799247000
C	-3.059263000	-1.667840000	1.889479000
H	-3.047505000	-0.621292000	2.195974000
C	-4.313846000	-2.332705000	2.470062000
H	-4.365226000	-2.159716000	3.551021000
H	-4.311329000	-3.418097000	2.312091000
H	-5.226396000	-1.933429000	2.015827000
C	-1.793661000	-2.314984000	2.466081000
H	-1.813180000	-2.301565000	3.561825000
H	-0.902656000	-1.756235000	2.160029000
H	-1.695655000	-3.363769000	2.151320000
C	-2.725211000	0.681395000	-2.650639000
H	-2.404356000	1.486087000	-1.985559000
C	-1.698371000	0.585557000	-3.782398000
H	-0.700707000	0.387836000	-3.387243000
H	-1.655626000	1.537522000	-4.323853000
H	-1.951387000	-0.194094000	-4.511824000
C	-4.108821000	1.018526000	-3.230219000
H	-4.881985000	1.036932000	-2.455622000
H	-4.408447000	0.271776000	-3.976680000
H	-4.092117000	1.998778000	-3.720811000
N	-2.576217000	0.719424000	0.286537000
K	-0.164537000	-2.144625000	-0.427770000
C	1.441251000	1.544869000	0.057685000
H	1.451341000	2.047995000	1.039343000
C	1.834610000	2.658135000	-1.001067000

C	3.382907000	2.561369000	-1.109261000
H	3.672388000	2.211643000	-2.108917000
H	3.868425000	3.533518000	-0.960460000
C	3.834988000	1.526712000	-0.060833000
C	1.205771000	2.409263000	-2.377844000
H	1.599341000	3.128286000	-3.109340000
H	0.116746000	2.516346000	-2.336485000
H	1.424321000	1.402431000	-2.745151000
C	1.416378000	4.060592000	-0.553759000
H	1.754452000	4.808124000	-1.283138000
H	1.848910000	4.325423000	0.418720000
H	0.328334000	4.134656000	-0.475952000
C	4.134168000	2.252378000	1.273283000
H	4.907441000	3.013046000	1.113301000
H	4.502479000	1.563107000	2.036205000
H	3.252133000	2.762280000	1.670860000
C	5.103890000	0.784542000	-0.475388000
H	5.027673000	0.371413000	-1.482513000
H	5.337247000	-0.034088000	0.215813000
H	5.950497000	1.479780000	-0.468074000
C	2.668106000	-0.737140000	0.153613000
C	2.818854000	-1.578832000	-0.985054000
C	2.819470000	-2.970886000	-0.830358000
H	2.947887000	-3.605291000	-1.704601000
C	2.650604000	-3.561059000	0.416541000
H	2.649130000	-4.643260000	0.519904000
C	2.518712000	-2.746263000	1.535078000
H	2.425007000	-3.205753000	2.515404000
C	2.557304000	-1.350901000	1.430634000
C	2.943245000	-1.030694000	-2.397696000
H	3.034293000	0.052627000	-2.310708000
C	4.175176000	-1.575676000	-3.135089000
H	4.332131000	-1.018423000	-4.065560000
H	4.050597000	-2.631470000	-3.404831000

H	5.083473000	-1.495564000	-2.530091000
C	1.677894000	-1.316521000	-3.216602000
H	1.763407000	-0.893247000	-4.223865000
H	0.800643000	-0.859000000	-2.749836000
H	1.506936000	-2.396563000	-3.325985000
C	2.616464000	-0.538124000	2.710815000
H	2.418274000	0.503864000	2.447220000
C	1.599887000	-0.967438000	3.773130000
H	0.586082000	-0.985313000	3.373044000
H	1.613442000	-0.260040000	4.609628000
H	1.828881000	-1.958898000	4.182262000
C	4.035475000	-0.643858000	3.298182000
H	4.805773000	-0.437556000	2.549431000
H	4.214416000	-1.656476000	3.680828000
H	4.163249000	0.060238000	4.128630000
N	2.639961000	0.666517000	-0.004409000

**6**			
N	-2.430478000	0.449799000	-0.625852000
N	2.454081000	0.560869000	0.510385000
C	-1.305626000	1.243078000	-0.639247000
C	-3.585347000	0.971296000	-1.415323000
C	-4.716590000	1.459945000	-0.499362000
H	-5.510000000	1.907578000	-1.107537000
H	-4.383376000	2.205355000	0.224340000
H	-5.148501000	0.617148000	0.048995000
C	-4.186996000	-0.074597000	-2.357903000
H	-5.012700000	0.387300000	-2.910475000
H	-4.587430000	-0.932504000	-1.808767000
H	-3.457175000	-0.431126000	-3.087338000
C	-2.913407000	2.096775000	-2.211671000
H	-3.590769000	2.941192000	-2.377875000
H	-2.621595000	1.711755000	-3.196326000
C	-1.649244000	2.505238000	-1.432592000

C	-1.970415000	3.667873000	-0.472629000
H	-2.291240000	4.548796000	-1.042960000
H	-1.098114000	3.947608000	0.118292000
H	-2.767464000	3.411759000	0.230222000
C	-0.555847000	2.945890000	-2.410956000
H	-0.919731000	3.784587000	-3.017950000
H	-0.283055000	2.130010000	-3.086610000
H	0.350170000	3.275047000	-1.897703000
C	-2.587995000	-0.763634000	0.129546000
C	-3.012166000	-0.705083000	1.472564000
C	-3.029460000	0.582499000	2.282475000
H	-2.783292000	1.411173000	1.612946000
C	-1.927108000	0.514886000	3.351564000
H	-1.811754000	1.484896000	3.848602000
H	-0.969566000	0.236859000	2.904900000
H	-2.170623000	-0.232146000	4.116520000
C	-4.380475000	0.878249000	2.946699000
H	-4.338513000	1.843172000	3.465298000
H	-4.631143000	0.116646000	3.694034000
H	-5.200054000	0.916643000	2.224140000
C	-3.314664000	-1.901771000	2.125739000
H	-3.648634000	-1.874609000	3.159509000
C	-3.168984000	-3.127099000	1.491640000
H	-3.411007000	-4.046876000	2.016788000
C	-2.666734000	-3.171581000	0.199198000
H	-2.494240000	-4.135124000	-0.272255000
C	-2.356604000	-2.002900000	-0.499328000
C	-1.682183000	-2.125480000	-1.856104000
H	-1.605579000	-1.124719000	-2.291295000
C	-0.251995000	-2.642213000	-1.664756000
H	0.270231000	-2.696657000	-2.625703000
H	-0.248925000	-3.644663000	-1.221185000
H	0.319590000	-1.985984000	-1.005027000
C	-2.442487000	-3.029914000	-2.835873000

H	-1.984941000	-2.970069000	-3.830155000
H	-3.497396000	-2.754471000	-2.926834000
H	-2.398846000	-4.079369000	-2.522692000
C	1.412030000	1.533129000	0.129062000
H	1.637495000	1.975041000	-0.858479000
C	3.664007000	1.270893000	0.960259000
C	4.530790000	1.856294000	-0.173968000
H	5.347982000	2.451941000	0.250030000
H	3.959756000	2.510173000	-0.840532000
H	4.976861000	1.056408000	-0.773310000
C	4.562547000	0.388728000	1.825660000
H	5.420116000	0.971742000	2.180741000
H	4.945638000	-0.472044000	1.266453000
H	4.018695000	0.022536000	2.698852000
C	3.024976000	2.388400000	1.794874000
H	3.661545000	3.279994000	1.843803000
H	2.894369000	2.027127000	2.821553000
C	1.640013000	2.701407000	1.170628000
C	1.689747000	4.073731000	0.486278000
H	1.879615000	4.860727000	1.226754000
H	0.756788000	4.323850000	-0.023811000
H	2.492743000	4.116895000	-0.258230000
C	0.589097000	2.711354000	2.285432000
H	0.820103000	3.488621000	3.025776000
H	0.582729000	1.748493000	2.804398000
H	-0.423007000	2.893843000	1.906578000
C	2.566210000	-0.757603000	-0.032104000
C	2.373533000	-1.860329000	0.840065000
C	1.830202000	-1.709589000	2.253990000
H	1.733002000	-0.636351000	2.445680000
C	0.435551000	-2.348789000	2.369648000
H	0.012730000	-2.165371000	3.365042000
H	0.487399000	-3.435121000	2.225038000
H	-0.261363000	-1.950593000	1.628592000

C	2.752525000	-2.327082000	3.317238000
H	2.402268000	-2.061463000	4.321898000
H	3.788761000	-1.993204000	3.214946000
H	2.753480000	-3.421981000	3.251973000
C	2.632135000	-3.152812000	0.379014000
H	2.496081000	-3.995988000	1.051803000
C	3.041496000	-3.387245000	-0.925113000
H	3.243754000	-4.399388000	-1.265590000
C	3.150842000	-2.317745000	-1.800743000
H	3.417415000	-2.507930000	-2.837190000
C	2.908336000	-1.006609000	-1.383558000
C	2.919850000	0.084639000	-2.443913000
H	2.923834000	1.048398000	-1.940552000
C	4.149959000	0.047844000	-3.359404000
H	4.169858000	0.940570000	-3.995341000
H	4.141722000	-0.823821000	-4.023767000
H	5.080880000	0.018829000	-2.783813000
C	1.629767000	0.008855000	-3.274529000
H	1.587182000	0.832916000	-3.997398000
H	0.745476000	0.065394000	-2.629728000
H	1.579068000	-0.934796000	-3.831444000
B	-0.037075000	0.837093000	0.090746000
H	-0.122876000	-0.178364000	0.712920000

References

1 R. Jazzar, R. D. Dewhurst, J.-B. Bourg, B. Donnadieu, Y. Canac and G. Bertrand, *Angew. Chem. Int. Ed*. 2007, **46**, 2899.

2 D. Auerhammer, M. Arrowsmith, H. Braunschweig, R. D. Dewhurst, J. O. C. Jiménez-Halla and T. Kupfer, *Chem. Sci*. 2017, **8**, 7066.

3 N. Kuhn and T. Kratz, *Synthesis* 1993, **6**, 561.

4 S. Stoll, A. Schweiger, *J. Magn. Reson.* 2006, **178**, 42.

5 G. Sheldrick, *Acta Cryst*. 2015, **A71**, 3.

6 G. Sheldrick, *Acta Cryst*. 2008, **A64**, 112.

7 A. L. Spek, *Acta Cryst*., 2015, **C71**, 9.

8 J.-D. Chai and M. Head-Gordon, M. *Phys. Chem. Chem. Phys*. 2008, **10**, 6615.

9 R. Seeger and J. A. Pople, *J. Chem. Phys.* 1977, **66**, 3045.

10 R. Bauernschmitt and R. Ahlrichs, *J. Chem. Phys.* 1996, **104**, 9047.

11 C. M. Brememan and K. B. Wiberg, *J. Comp. Chem.* 1990, **11**, 361.

12 M. J. Frisch, G. W. Trucks, H. B. Schlegel, G. E. Scuseria, M. A. Robb, J. R. Cheeseman, G. Scalmani, V. Barone, B. Mennucci, G. A. Petersson, H. Nakatsuji, M. Caricato, X. Li, H. P. Hratchian, A. F. Izmaylov, J. Bloino, G. Zheng, J. L. Sonnenberg, M. Hada, M. Ehara, K. Toyota, R. Fukuda, J. Hasegawa, M. Ishida, T. Nakajima, Y. Honda, O. Kitao, H. Nakai, T. Vreven, J. A. Montgomery, Jr., J. E. Peralta, F. Ogliaro, M. Bearpark, J. J. Heyd, E. Brothers, K. N. Kudin, V. N. Staroverov, R. Kobayashi, J. Normand, K. Raghavachari, A. Rendell, J. C. Burant, S. S. Iyengar, J. Tomasi, M. Cossi, N. Rega, J. M. Millam, M. Klene, J. E. Knox, J. B. Cross, V. Bakken, C. Adamo, J. Jaramillo, R. Gomperts, R. E. Stratmann, O. Yazyev, A. J. Austin, R. Cammi, C. Pomelli, J. W. Ochterski, R. L. Martin, K. Morokuma, V. G. Zakrzewski, G. A. Voth, P. Salvador, J. J. Dannenberg, S. Dapprich, A. D. Daniels, O. Farkas, J. B. Foresman, J. V. Ortiz, J. Cioslowski and D. J. Fox, Gaussian 16, Revision B.01; Gaussian, Inc.: Wallingford CT, 2016.

13 ADF-GUI, version 2018.105; *Scientific Computing and Modeling*, Amsterdam, The Netherlands, 2018.

14 R. D. Johnson III, *NIST Computational Chemistry Comparison and Benchmark Database. NIST Standard Reference Database Number 101*. Release 20, August 2019.
